# Pharyngeal electrical stimulation for neurogenic dysphagia following stroke, traumatic brain injury or other causes: Main results from the PHADER cohort study

**DOI:** 10.1016/j.eclinm.2020.100608

**Published:** 2020-11-10

**Authors:** Philip M. Bath, Lisa J. Woodhouse, Sonja Suntrup-Krueger, Rudolf Likar, Markus Koestenberger, Anushka Warusevitane, Juergen Herzog, Michael Schuttler, Suzanne Ragab, Lisa Everton, Christian Ledl, Ernst Walther, Leopold Saltuari, Elke Pucks-Faes, Christof Bocksrucker, Milan Vosko, Johanna de Broux, Claus G. Haase, Alicja Raginis-Zborowska, Satish Mistry, Shaheen Hamdy, Rainer Dziewas, for PHADER Investigators

**Affiliations:** a Stroke Trials Unit, Division of Clinical Neuroscience, University of Nottingham, Nottingham NG5 1PB, United Kingdom; b Stroke, Nottingham University Hospital NHS Trust, Nottingham NG5 1PB, United Kingdom; c Department of Neurology, University Hospital Münster, Building A1, Albert-Schweitzer-Campus 1, 48149 Münster, Germany; d Department of Anaesthesiology and Intensive Care Medicine, Klinikum Klagenfurt am Wörthersee, Klagenfurt am Wörthersee, Austria; e Stroke Research, Royal Stoke University Hospital, University Hospitals of North Midlands NHS Trust, Parish Building, 1st Floor, Newcastle Road, Stoke-on-Trent, Staffordshire ST4 6QG, United Kingdom; f Clinic for Neurological Rehabilitation and Early Rehabilitation, Schön Klinik München-Schwabing, Parzivalplatz 4, 80804 Munich, Germany; g Centre of Neurology, Schön Klinik Bad Staffelstein, Am Kurpark 11, 96231 Bad Staffelstein, Germany; h Department of Stroke, Philip Arnold Unit Ground Floor, Poole Hospital NHS Foundation Trust, Longfleet road, Poole BH15 2JB, United Kingdom; i Speech and Language Therapy, Nottinghamshire Healthcare NHS Foundation Trust, Nottingham NG3 6AA, United Kingdom; j Specialist Clinic for Neurology, Neurological Rehabilitation and Alzheimer's Therapy, Schön Klinik Bad Aibling, Kolbermoorer Strasse 72, 83043 Bad Aibling, Germany; k Clinic for Neurology and Neurorehabilitation, Schön Klinik Hamburg Eilbek, Hamburg, Germany; l Department of Neurology, Ö. Landeskrankenhaus Hochzirl-Natters, Tiroler landesrankenanstalten GmbH. LkH Hochzirl, 6170 Zirl/Hochzirl, Austria; m Department of Neurology, Konventhospital Barmherzige Brúder Linz, Seilerstätte 2, 4021 Linz, Austria; n Department of Neurology 2, Kepler Universitätsklinikum, Med Campus III, Krankenhausstrasse 9, 4020 Linz, Austria; o Clinic for Neurology, Alexianer Krefeld GmbH, Dießemer Bruch 81, 47805 Krefeld, Germany; p Clinic for Neurology and Neurophysiology, Evangelische Kliniken Gelsenkirchen, Lehrkrankenhaus der Universität Essen-Duisburg, Munckelstr. 27, 45879 Gelsenkirchen, Germany; q Department for Clinical Research, Phagenesis Limited, Manchester M15 6SE, United Kingdom; r Centre for Gastrointestinal Sciences, Faculty of Biology, Medicine and Health, University of Manchester and the Manchester Academic Health Sciences Centre, Manchester M6 8HD, United Kingdom

**Keywords:** Decannulation, Dysphagia, Pharyngeal electrical stimulation, Stroke, Traumatic brain injury, Ventilation

## Abstract

**Background:**

Neurogenic dysphagia is common and has no definitive treatment. We assessed whether pharyngeal electrical stimulation (PES) is associated with reduced dysphagia.

**Methods:**

The PHAryngeal electrical stimulation for treatment of neurogenic Dysphagia European Registry (PHADER) was a prospective single-arm observational cohort study. Participants were recruited with neurogenic dysphagia (comprising five groups – stroke not needing ventilation; stroke needing ventilation; ventilation acquired; traumatic brain injury; other neurological causes). PES was administered once daily for three days. The primary outcome was the validated dysphagia severity rating scale (DSRS, score best-worst 0–12) at 3 months.

**Findings:**

Of 255 enrolled patients from 14 centres in Austria, Germany and UK, 10 failed screening. At baseline, mean (standard deviation) or median [interquartile range]: age 68 (14) years, male 71%, DSRS 11·4 (1·7), time from onset to treatment 32 [44] days; age, time and DSRS differed between diagnostic groups. Insertion of PES catheters was successfully inserted in 239/245 (98%) participants, and was typically easy taking 11·8 min. 9 participants withdrew before the end of treatment. DSRS improved significantly in all dysphagia groups, difference in means (95% confidence intervals, CI) from 0 to 3 months: stroke (*n* = 79) –6·7 (–7·8, –5·5), ventilated stroke (*n* = 98) –6·5 (–7·6, –5·5); ventilation acquired (*n* = 35) –6·6 (–8·4, –4·8); traumatic brain injury (*n* = 24) -4·5 (–6·6, –2·4). The results for DSRS were mirrored for instrumentally assessed penetration aspiration scale scores. DSRS improved in both supratentorial and infratentorial stroke, with no difference between them (*p* = 0·32). In previously ventilated participants with tracheotomy, DSRS improved more in participants who could be decannulated (*n* = 66) –7·5 (–8·6, –6·5) versus not decannulated (*n* = 33) –2·1 (–3·2, –1·0) (*p*<0·001). 74 serious adverse events (SAE) occurred in 60 participants with pneumonia (9·2%) the most frequent SAE.

**Interpretation:**

In patients with neurogenic dysphagia, PES was safe and associated with reduced measures of dysphagia and penetration/aspiration.

**Funding:**

Phagenesis Ltd.

**Research in context**

**Evidence before this study**

Pharyngeal electrical stimulation (PES) is a potential treatment for neurogenic dysphagia and was associated with less dysphagia (assessed using the dysphagia severity rating scale, DSRS) and instrumentally-assessed penetration/aspiration (penetration aspiration scale, PAS) in an individual-patient data meta-analysis of 3 pilot trials in stroke patients. The phase III STEPS trial of PES for post-stroke dysphagia was neutral on PAS and DSRS, probably because dysphagia was mild at baseline, the active group were undertreated and the sham group received some treatment. In the PHAST-TRAC trial involving a more severe group of patients with post-stroke dysphagia who had required ventilation and could not then have their tracheotomy cannula removed due to persistent dysphagia, PES facilitated decannulation as compared with sham treatment, both in phase II and III trials. A phase II trial in multiple sclerosis showed improvements in PAS score. PES has a CE mark for neurogenic dysphagia.

**Added value of this study**

This observational cohort study included 245 adults with neurogenic dysphagia related to stroke, traumatic brain injury or following mechanical ventilation and tracheotomy. PES was safe and associated with a significant improvement in oropharyngeal dysphagia and reduced penetration/aspiration risk both overall and in each diagnostic group.

**Implications of all the available evidence**

In neurogenic dysphagia, pharyngeal electrical stimulation is associated with less dysphagia and penetration/aspiration.

Alt-text: Unlabelled box

## 1 Introduction

Neurogenic dysphagia is common in conditions such as stroke and traumatic brain injury (TBI) and is associated with a poor outcome [1]. Although there is no proven treatment, potential efficacious interventions for post-stroke dysphagia (PSD) include acupuncture and behavioural therapies [2]. Pharyngeal electrical stimulation (PES) is a potential treatment for neurogenic dysphagia based on physiological studies [3]. PES has been studied in several phase II trials in patients with PSD [4,5] and was associated with less dysphagia (assessed using the dysphagia severity rating scale, DSRS [4,6]) and instrumentally-assessed penetration/aspiration (penetration aspiration scale, PAS [7]) in an individual-patient data meta-analysis [8]. However, a phase III trial of PES for PSD was neutral on PAS and DSRS, possibly because dysphagia was mild at baseline, the active group were undertreated and the sham group received some treatment [9]. In a more severe group of patients with PSD, specifically those who had required ventilation and could not then have their tracheotomy cannula removed due to persistent dysphagia, PES facilitated decannulation as compared with sham treatment, both in phase II and III trials [10,11]. Moreover, PES has been studied in other neurogenic causes of dysphagia and a phase II trial in multiple sclerosis showed improvements in PAS score [12].

PES has a European *Conformité Européenne* (CE) mark for the treatment of neurogenic dysphagia and US Food & Drug Administration breakthrough designation. Here we report the results of a prospective observational cohort study designed to assess the real-world clinical outcome and safety of PES for reducing neurogenic dysphagia.

## 2 Methods

### 2.1 Objectives

Sensorimotor pathways associated with swallowing are susceptible to damage from a variety of neurological insults, broadly categorisable as either non-progressive (e.g. stroke, TBI, critical illness polyneuropathy, Guillain-Barre syndrome) or progressive (e.g. dementia, multiple sclerosis, Parkinson's disease). Since it is the same pathways being damaged, PES targets the resulting dysphagia rather than the initial causative disease. Hence, the primary objective of the study was to assess the real-world effect of PES on dysphagia severity (assessed using the validated dysphagia severity rating scale, DSRS [4,6]) in patients with neurogenic dysphagia. Secondary objectives assessed the effect of PES on penetration/aspiration (PAS [7]) determined using instrumental-testing; feasibility, tolerability and safety of PES; and its ease of use.

### 2.2 Study design

PHADER was a prospective single-arm observational clinical cohort study. The study was performed in secondary and tertiary hospitals caring for patients with stroke, TBI or other neurological conditions, and who had dysphagia. The study protocol is available at http://www.phagenesis.com/wp-content/uploads/2020/06/AHE02-PHADER_CIP.pdf. This report follows The Strengthening the Reporting of Observational Studies in Epidemiology (STROBE) Statement: guidelines for reporting observational studies.

### 2.3 Setting

Recruitment and follow-up took place between March 2015 and September 2018 at 14 secondary/tertiary care centres in Austria, Germany and UK. Analyses were completed in April 2020.

### 2.4 Study population

Patients were eligible for the study if they were adults, had oropharyngeal dysphagia with a DSRS score of 6 or higher, and belonged to one of the following diagnostic groups: dysphagia related to (A) stroke not requiring mechanical ventilation; (B) stroke requiring mechanical ventilation and tracheotomy; (C) mechanical ventilation in non-stroke, non-TBI; (D) TBI with or without the need for mechanical ventilation and tracheotomy; and (E) any other neurological cause not needing mechanical ventilation and tracheotomy. Key exclusion criteria were: non-neurogenic dysphagia (e.g. cancer), presence of an implanted cardiac pacemaker or cardioverter defibrillator, pregnancy or a nursing mother. Full inclusion and exclusion criteria are listed in the Supplement 1 (page 4) and given in the uploaded statistical analysis plan.

### 2.5 Approvals and training

The study was funded and sponsored by Phagenesis Ltd (Manchester UK) and approved by National/Local Research Ethics Committees. Patients signed a standard Research Ethics Committee approved Informed Consent Form explaining the conditions of study participation; where allowed, a legal representative of patients lacking capacity gave proxy consent. All sites received face-to-face training in the study protocol and delivery of PES.

### 2.6 Intervention

The device used was the CE-marked Phagenyx Base Station® and Phagenyx Catheter® (Phagenesis Ltd, Manchester UK); the CE-mark covers the treatment of neurogenic dysphagia and devices were used as marketed and were not investigational. The treatment catheter is a nasogastric feeding tube with built-in stimulation electrodes, with stimulation provided at 5 Hz for 10 min on each of three consecutive days [4,9,11]. Stimulation was optimised for each treatment by the Base Station software and operator, and intensity set at 75% of the tolerable limit above sensory threshold. The catheter houses a microchip that allows the application of the therapy on three occasions on consecutive days.

### 2.7 Outcomes

The primary outcome measure was the validated 13-level DSRS score [4,6] at 3 months post-treatment. Secondary outcomes comprised dysphagia severity assessed using the functional oral intake scale (FOIS) [13], and penetration-aspiration assessed with the PAS [7] measured instrumentally (using videofluoroscopy (VFS) or fibreoptic endoscopic evaluation of swallowing (FEES)). Assessments were made at baseline and then at days 5 (range 4–6) and 9 (range 7–21), and 3 months (range 2–4) after catheter insertion. Ease of catheter insertion and time required for insertion was determined after enrolment. Treatment optimisation parameters (threshold, tolerance and stimulation intensity) were recorded on each of the three treatment days. The protocol for decannulation followed that used in the PHAST-TRAC trial [11]; time-to-decannulation was determined during follow-up, and feeding status [14], serious adverse events (SAEs) and deaths measured at month 3.

### 2.8 Statistical analyses

Sample size was set at 60 participants per diagnostic groups so that the presence of a device deficiency in 5% of the population could be ruled out with confidence of 80%. With 5 groups, the intended total sample size was 300. Group E (other neurogenic dysphagia) was expected to recruit at about half the ideal rate; with redistribution of group E patients the total sample would remain at 300. A statistical analysis plan was developed prior to completion of data collection and lock (Supplement 2; first draft 10 April 2015, updated 12 May 2019, finalised 30 Aug 2019; data lock 30 Oct 2019). Analyses are by intention to treat and results are presented for all participants, for each of the diagnostic groups A-E, by stroke location (supratentorial, infratentorial for groups A, B) and by whether tracheotomised patients were decannulated or not (groups B-D).

A substudy compared the effect of PES on DSRS in non-ventilated stroke patients (PHADER group A) with the control group comprising patients in the STEPS trial [9] who had been randomised to receive sham treatment.

Data are shown as number (%), median [interquartile range, IQR] or mean (standard deviation, SD); difference in means (DIM), mean difference (MD), odds ratio (OR) and 95% confidence intervals (95% CI). Analyses used Fisher's exact test (baseline data), Chi-square test (baseline data, discharge disposition, cannulation status), paired *t*-test (DSRS, FOIS, PAS; to focus on participants not lost to follow-up and so reduce bias), unpaired *t*-test (unpooled, DSRS, FOIS, PAS), Kruskal-Wallis test (baseline data, days), one-way analysis of variance (baseline data, times, ease of use, stimulation levels), analysis of covariance (ANCOVA), ordinal logistic regression (OLR) and multiple linear regression (MLR). The proportional odds assumption was tested using the likelihood ratio test; in each case, the assumption of proportional odds was not violated (all *p*>0.05). MLR assumptions were tested for evidence of linear relationships, multivariate normality and absence of multicollinearity; similarly, these assumptions were not violated. Regression analyses were adjusted for age, sex, NIHSS, mRS, stroke type, time from stroke onset to treatment and baseline value (adjustment variables are all prognostic for recovery after stroke). The primary outcome was examined in the pre-specified diagnostic groups, stroke location (supra/infra-tentorial), and decannulation status. A cumulative plot of time to hospital discharge and/or re-start of oral feeding is given. No imputation was performed for missing data, and no adjustment was made for multiplicity of testing. *P*<0·05 is considered significant; analyses were performed using SAS (version 9.4, SAS Institute).

### 2.9 Role of the funder/sponsor

The funder was involved in the design and conduct of the study and data management, and compensated sites for data collection. A clinical research organisation (FAKKEL, Belgium) performed study management and source data verification. Most analyses were performed by Cytel Corp as specified by Phagenesis Ltd and the Study Steering Committee. The funder reviewed and approved the manuscript. All authors had full access to all data. The corresponding author had final responsibility for the decision to submit for publication.

## 3 Results

**Fig. 1:**
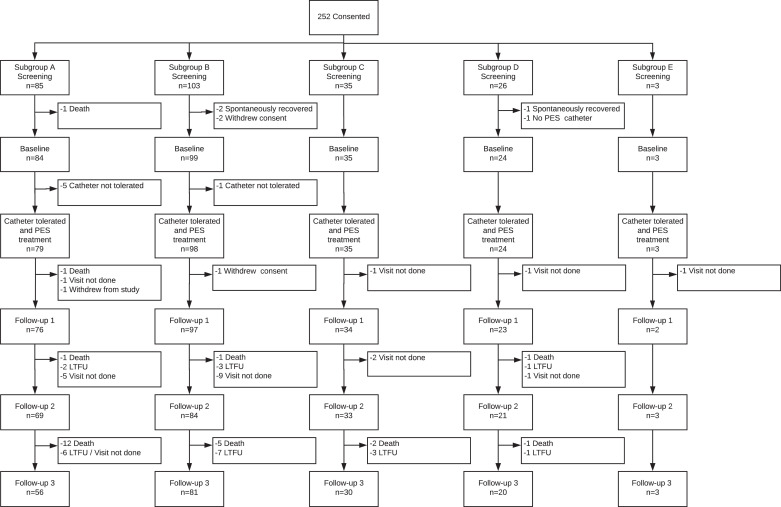
Study population.

Due to a limited population of patients fulfilling the criteria for Group E (as anticipated above), and to a lesser extent non-stroke ventilator-related and TBI (Groups C, D), the trial was stopped after recruitment of 252 patients. Of these, 7 were excluded from analysis due to lack or withdrawal of consent, spontaneous recovery or unavailability of a catheter or death (Fig. 1). 6 participants had a failed attempt at catheter insertion but are included in the analyses (intention-to-treat). Although recommended in the protocol, not all recruiting sites kept screening logs and so the total number of patients screened for the study is not known. By diagnostic group, 84 had an index stroke not requiring mechanical ventilation (group A); 99 had an index stroke requiring mechanical ventilation and tracheotomy (group B); 35 had dysphagia related to a non-stroke/non-TBI cause (group C) with 15 of these due to critical illness polyneuropathy (Supplement 1, Table I); 24 had a TBI (group D); and 3 had another cause for their dysphagia (group E, Supplement 1, Table I). Abbreviated results on 15 participants in group C who presented to one centre have been published previously [15]. Due to the limited number of patients in Group E (*N* = 3) it was deemed permissible that these participants should not be included in most analyses. Overall, the average age was 68 (14) years although this varied significantly between groups with TBI patients the youngest (63 years) and non-ventilated stroke the oldest (74 years) (Table 1). The majority of patients were male (71%) and 17% of stroke participants had an infratentorial lesion. Time from onset to treatment averaged 32 [44] days and differed between the groups being shortest in stroke patients 16 [24] days and longest in TBI 73 [154] days. In patients enrolled with a stroke, approximately one-third received thrombolysis; a similar proportion received mechanical thrombectomy (with an overlap in these).

**Table 1:** Baseline characteristics by diagnostic group in participants where catheterization was attempted or succeeded. Data are number (%), median [interquartile range] or mean (standard deviation); comparison of groups by Chi-square test, Kruskal–Wallis test or one-way analysis of variance.

	N	All	Stroke, not ventilated	Stroke, ventilated	Ventilator-related^a^	TBI	Other	p-value
N		245	84	99	35	24	3	
Age	245	68·2 (14·2)	73·7 (12·7)	66·4 (13·1)	64·7 (14·7)	62·2 (16·4)	61·0 (21·5)	<0·001
Sex, male (%)	245	173 (70·6)	58 (69·0)	73 (73·7)	22 (62·9)	19 (79·2)	1 (33·3)	0.49
OTT (days)	237	32·0 [44·0]	16·0 [24·0]	30·5 [35·0]	43·5 [42·0]	73·0 [153·5]	169·0 [224·0]	<0·001
Feeding status	245							0·004
Oral, normal		0 (0·0)	0 (0·0)	0 (0·0)	0 (0·0)	0 (0·0)	0 (0·0)	
Oral, supervision		5 (2·0)	4 (4·8)	1 (1·0)	0 (0·0)	0 (0·0)	0 (0·0)	
Oral, with support		4 (1·6)	4 (4·8)	0 (0·0)	0 (0·0)	0 (0·0)	0 (0·0)	
NGT or NJT		151 (61·6)	50 (59·5)	71 (71·7)	22 (62·9)	8 (33·3)	0 (0·0)	
PEG or RIG		76 (31·0)	21 (25·0)	25 (25·3)	12 (34·3)	15 (62·5)	3 (100·0)	
Other route		9 (3·7)	5 (6·0)	2 (2·0)	1 (2·9)	1 (4·2)	0 (0·0)	
GCS (/15)	164	12·9 (2·7)	14·0 (1·8)	12·8 (2·6)	12·9 (3·1)	10·5 (4·0)	14·0 (-)	<0·001
NIHSS (/42)	151	11·9 (7·4)	10·6 (8·5)	13·3 (5·8)	–	–	–	0·024
mRS (/6)	170	5·0 [1·0]	4·5 [1·0]	5·0 [1·0]	–	–	–	<0·001
Stroke, ischaemic	183	153 (83·6)	78 (92·9)	74 (75·5)	–	–	–	0·002
Lesion location	183							0·44
Right		59 (32·2)	26 (31·0)	33 (33·7)	–	–	–	
Left		75 (41·0)	37 (44·0)	38 (38·8)	–	–	–	
Bilateral		18 (9·8)	5 (6·0)	12 (12·2)	–	–	–	
Infratentorial		31 (16·9)	16 (19·0)	15 (15·3)	–	–	–	
Tracheal cannula	245	99 (40·4)	–	60 (60·6)	23 (65·7)	16 (66·7)	–	0·79
Oxygen use	237	85 (35·9)	15 (18·1)	48 (50·5)	18 (52·9)	4 (18·2)	0 (0·0)	0·016
Dysphagia assessment	244							<0·001
Bedside		46 (18·9)	33 (39·8)	8 (8·1)	3 (8·6)	2 (8·3)	0 (0·0)	
VFS		4 (1·6)	3 (3·6)	0 (0·0)	1 (2·9)	0 (0·0)	0 (0·0)	
FEES		186 (76·2)	41 (49·4)	90 (90·9)	30 (85·7)	22 (91·7)	3 (100·0)	
VFS + FEES		9 (3·3)	6 (7·2)	1 (1·0)	1 (2·9)	0 (0·0)	0 (0·0)	
Ventilation (days)	129	22·0 [18·0]	–	19·0 [18·0]	25·0 [22·0]	30·5 [16·0]	–	0·065

GCS: Glasgow coma scale; mRS: modified Ranking Scale; NGT: nasogastric tube; NIHSS: National Institute Health Stroke Scale; NJT: nasojejunal tube; OTT: onset to treatment; PEG: percutaneous endoscopic gastrostomy tube; RIG: radiographically inserted gastrostomy tube; TBI: traumatic brain injury.

a Not stroke or TBI (see Supplement 1, Table I).

On average it took just under 12 min to insert the catheter (Supplement 1, Table II) and this was reported to be easy with mean scores of >5 out of 7. Overall, 1·1 catheters were used per participant. There were no differences between the diagnostic groups with respect to user experience. Although threshold levels did not differ between the groups, tolerance and therefore stimulation levels were highest in stroke patients who had been ventilated (30·9 mA on day 1) and lowest in non-ventilated stroke patients (23·8 mA) (Supplement 1, Table III). Stimulation levels did not change over the three days of treatment.

### 3.1 Primary outcome

**Fig. 2:**
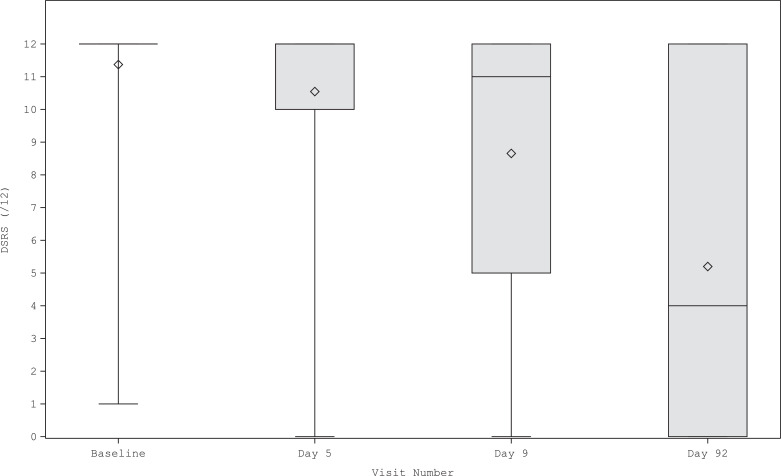
Box and whisker plot of dysphagia severity rating scale across all patient groups. Figure shows 5th centile, 25th centile, box containing median (horizontal line) and mean (diamond), 75th centile and 95th centile at each timepoint.

**Fig. 3:**
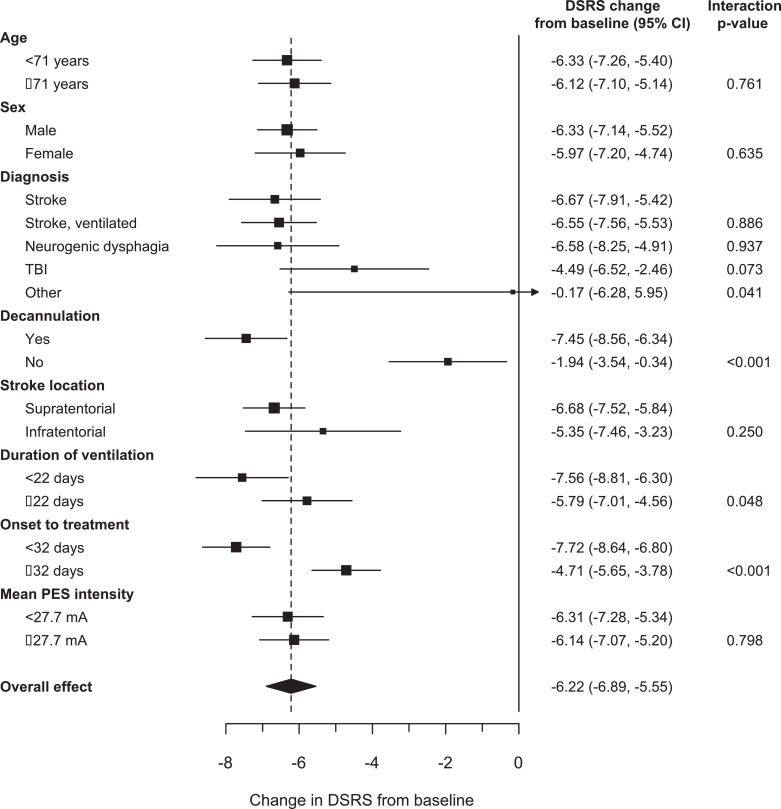
Forest plot of change in dysphagia severity rating scale (DSRS) from baseline to three months in pre-specified subgroups: age (below/above median 71 years), sex (male/female), diagnosis (groups A-E), decannulation (yes/no), stroke location (supratentorial/infratentorial), duration of ventilation (below/above median 22 days), onset to treatment (below/above median 32 days) and mean stimulation intensity (over 3 days, below/above median 27·7 mA). The dotted line gives the overall effect; if a square and horizontal line do not overlap the dotted line then they differ significantly from the overall effect size.

Participants were severely dysphagic at baseline (mean DSRS 11·4 of total score 12); however, severity varied between the groups and was highest in participants who received mechanical ventilation and lowest in non-ventilated stroke (Table 2). DSRS fell significantly over 3 months of observation (Fig. 2) by more than 6 points, both overall and in three of the four diagnostic groups (A, B and C) over the 3 months of follow-up; less decline (4·5 points), albeit still statistically significant, was seen in TBI (Table 2). In those participants who had DSRS scores at three months as well as at baseline, the reduction in DSRS was 6·3 points. Improvement was seen in all three DSRS categories of fluids, diet and supervision, again both overall and in each diagnostic group (Supplement 1, Table IV). When assessed in pre-defined subgroups, the reduction in DSRS was greater in participants with shorter times from onset to treatment and duration of ventilation than those with longer times (Fig. 3).

**Table 2:** Dysphagia severity rating scale score (primary outcome), functional oral intake scale score, penetration aspiration scale score, length of stay in hospital, time from treatment to discharge, discharge destination and death by diagnostic group. Data are number of participants, mean (standard deviation), difference in means and mean difference (95% confidence interval); comparison of groups by analysis of variance, and day 92 versus baseline by paired and unpaired *t*-tests.

	All	Stroke, not ventilated	Stroke, ventilated	Ventilator-related^a^	TBI	*p*
N		79	98	35	24	
DSRS (/12)^b^						
Baseline	236, 11·4 (1·7)	79, 10·9 (2·4)	98, 11·7 (1·2)	35, 11·9 (0·5)	24, 11·3 (1·8)	0·003
Day 5	229, 10·5 (2·6)	74, 9·9 (2·9)	97, 10·8 (2·4)	35, 10·8 (2·5)	23, 11·0 (2·5)	
Day 9	224, 8·6 (3·9)	70, 7·7 (4·1)	97, 8·9 (3·8)	35, 8·5 (4·1)	22, 10·4 (3·1)	
Day 92	174, 5·1 (4·9)	46, 4·2 (4·2)	78, 5·2 (5·0)	30, 5·3 (5·4)	20, 6·8 (4·8)	0·26
DIM (unpaired)	−6·3 (−7·0, −5·6)^c^	−6·7 (−7·8, −5·5)^c^	−6·5 (−7·6, −5·5)^c^	−6·6 (−8·4, −4·8)^c^	−4·5 (−6·6, −2·4)^c^	0·31
MD (paired)	174, −6·3 (−7·0, −5·6)^c^	46, −6·5 (−7·9, −5·2)^c^	78, −6·5 (−7·6, −5·3)^c^	30, −6·6 (−8·5, −4·6)^c^	20, −4·7 (−6·8, −2·5)^c^	0·033
FOIS (/7)						
Baseline	220, 1·4 (0·9)	65, 1·7 (1·3)	97, 1·2 (0·6)	34, 1·1 (0·3)	24, 1·4 (0·7)	<0·001
Day 5	214, 1·8 (1·4)	63, 2·2 (1·5)	96, 1·8 (1·3)	32, 1·8 (1·4)	23, 1·5 (1·0)	
Day 9	213, 2·7 (1·9)	61, 3·2 (1·9)	96, 2·5 (1·9)	34, 3·0 (2·1)	22, 1·9 (1·5)	
Day 92	172, 4·3 (2·5)	42, 4·5 (2·3)	79, 4·3 (2·6)	31, 4·4 (2·7)	20, 3·4 (2·4)	0·38
DIM	2·9 (2·5, 3·3)^c^	2·8 (2·1, 3·5)^c^	3·1 (2·5, 3·6)^c^	3·3 (2·4, 4·3)^c^	2·0 (1·0, 3·0)	0·20
MD (paired)	170, 2·9 (2·5, 3·3)^c^	40, 2·8 (2·0, 3·5)^c^	79, 3·1 (2·5, 3·7)^c^	31, 3·3 (2·3, 4·3)^c^	20, 2·0 (0·9, 3·0)	0·042
PAS (/8)						
Baseline	144, 6·7 (1·7)	42, 6·2 (1·7)	53, 7·2 (1·2)	27, 6·8 (1·6)	22, 6·5 (2·4)	0·031
Day 5	89, 5·2 (2·5)	19, 4·3 (2·5)	39, 5·4 (2·4)	18, 4·9 (2·8)	13, 6·1 (2·4)	
Day 9	100, 4·4 (2·7)	21, 3·8 (2·6)	44, 4·3 (2·7)	20, 3·6 (2·7)	15, 6·7 (1·9)	
Day 92	68, 3·2 (2·6)	10, 2·8 (2·1)	31, 3·0 (2·6)	15, 2·2 (2·0)	12, 5·3 (2·7)	0·011
DIM	−3·5 (−4·1, −2·9)^c^	−3·4 (−4·7, −2·1)^c^	−4·2 (−5·0, −3·3)^c^	−4·6 (−5·8, −3·5)^c^	−1·2 (−3·0, 0·6)	0·003
MD (paired)	68, −4·1 (−4·8, −3·3)^c^	10, −3·8 (−6·3, −1·3)	31, −4·5 (−5·5, −3·4)^c^	15, −5·3 (−6·5, −4·1)^c^	12, −1·7 (−3·6, 0·3)	
Time intervals (days)						
Hospital stay	38·5 [53·0]	34·0 [42·0]	40·5 [62·0]	38·0 [51·0]	49·0 [59·0]	0·38
PES-discharge	36·5 [53·5]	32·0 [42·0]	38·0 [63·0]	36·0 [46·0]	47·0 [59·0]	0·49
Discharge disposition (%)						0·001
Acute care	16 (11·2)	3 (5·0)	10 (18·9)	1 (5·9)	2 (18·2)	
Sub-acute care	40 (28·0)	9 (15·0)	26 (49·1)	4 (23·5)	1 (9·1)	
Assisted care	6 (4·2)	5 (8·3)	0 (0·0)	0 (0·0)	1 (9·1)	
Full-nursing care	11 (7·7)	6 (10·0)	3 (5·7)	1 (5·9)	1 (9·1)	
Home care	44 (30·8)	22 (36·7)	7 (13·2)	9 (52·9)	4 (36·4)	
Death	26 (18·2)	15 (25·0)	7 (13·2)	2 (11·8)	2 (18·2)	

DIM: difference in means between month 3 and 0 (unpaired); DSRS: dysphagia severity rating scale; FOIS: functional oral intake scale; MD: mean difference between month 3 and 0 (paired); NGT: nasogastric tube; NJT: nasojejunal tube; OTT: onset to treatment; PAS; penetration aspiration scale; PEG: percutaneous endoscopic gastrostomy tube; RIG: radiographically inserted gastrostomy tube; TBI: traumatic brain injury.

a Not stroke or TBI.

b DSRS scored as sum of: • Fluids: 0 Normal fluids, 1 Syrup consistency, 2 Custard consistency, 3 Pudding consistency, 4 No oral fluids • Diet: 0 Normal diet, 1 Selected textures, 2 Soft/moist diet, 3 Puree, 4 Non-oral feeding • Supervision: 0 Eating independently, 1 Eating with supervision, 2 feeding by third party (untrained), 3 therapeutic feeding (trained), 4 no oral feeding.

c *p*<0·001.

#### 3.1.1 By stroke location

Participants with stroke came from Groups A and B. When compared with patients with supratentorial stroke, those with infratentorial stroke were of comparable age, sex, premorbid mRS and conscious level (GCS) at baseline but had less severe stroke (NIHSS) and penetration/aspiration (PAS), and required higher levels of stimulation current (Supplement 1, Table V, VI). Dysphagia severity (DSRS, FOIS) was comparable at baseline and PES was associated with improvements in DSRS and FOIS in both groups; the final DSRS score did not differ between participants with supratentorial and infratentorial stroke (Supplement 1, Table VI). In participants who were cannulated at baseline, decannulation was feasible in both supratentorial and infratentorial stroke, and rates did not differ between the two groups.

#### 3.1.2 By cannulation status in participants with a tracheotomy

Participants who were ventilated and required tracheotomy came from groups B and C and some of D. Participants who could be decannulated had a shorter onset time to treatment and were less likely to have a haemorrhagic stroke (Supplement 1, Table VII). Following treatment (PES stimulation levels did not differ between the groups), two-thirds of participants could be decannulated (Supplement 1, Table VII). Although the DSRS improved in both groups, the magnitude of improvement at three months was greater (7·5 vs 2·1 points) and the final DSRS lower in the decannulated than non-decannulated group (Supplement 1, Table VIII).

### 3.2 Secondary outcomes

Similar recovery to DSRS was seen for clinical dysphagia when assessed using the FOIS (which increased significantly by 2·9 points across the cohort) and for instrumentally-assessed penetration/aspiration (with the PAS falling significantly by 4·1 units) across all participants (Table 2, Supplement 1, Fig. I). Significant improvements in FOIS and PAS were present across all diagnostic groups although the magnitude of change was smaller for PAS in TBI participants. As with DSRS, FOIS and PAS improved in both supratentorial and infratentorial stroke (Supplement 1, Table VI). Although participants who could not be decannulated showed significant improvements in FOIS and PAS, the magnitude was smaller than that seen in those who could be decannulated (Supplement 1, Table VIII).

Length of stay in hospital did not differ between the diagnostic groups (range 34–49 days across the four groups) (Table 2), supratentorial versus infratentorial stroke (Supplement 1, Table VI) or participants who could or could not be decannulated (Supplement 1, Table VIII). 50% of participants had been discharged from hospital or had at least re-started oral feeding by 30 days (Supplement 1, Fig. III). Discharge disposition, including in-hospital death, varied between the groups (Table 2) with death higher in non-ventilated stroke participants (25·0%) than in the other groups (range 11·8–18·2%). Discharge disposition, including in-hospital death, did not differ between participants with supratentorial and infratentorial stroke (Supplement 1, Table VI) or those who could or could not be decannulated (Supplement 1, Table VIII).

### 3.3 Serious adverse events (SAEs)

Altogether, 74 SAEs occurred in 60 participants (1·2 SAE per participant, Supplement 1, Table IX) with 29 being fatal. Most SAEs occurred within the first 30 days after start of PES (Supplement 1, Fig. IV). The commonest SAE was pneumonia (27, 11·0%), most of which occurred in participants with a stroke that did not need ventilation (18%, Group A). The next most common SAEs were cardiac arrest (5, 2·0%, Supplement 1, page 5), respiratory failure (4, 1·6%) and recurrent stroke (3, 1·2%). Only one of the 74 SAEs was considered as "possibly" related to catheter insertion which was followed by chest sepsis. There were no differences in the risk of individual SAEs between baseline groups.

### 3.4 Comparison of PHADER and STEPS (for non-ventilated stroke participants)

Non-ventilated stroke patients who received active treatment in PHADER were compared with those randomised to sham treatment in the STEPS trial [9]. Although participants had similar ages, sex distribution and time from stroke to stimulation, those in PHADER had far more severe dysphagia at baseline (by 3·8 points on the 12 point DSRS scale), were more likely to have an ischaemic stroke, and received a higher treatment stimulation current (by 9·1 mA) than those in STEPS (Supplement 1, Table X). Although the SAP specified a parametric analysis, models were unstable and an ordinal analysis was performed. Following treatment, DSRS fell significantly in both groups but more so with active than sham treatment with a non-significant difference at 9–14 days of 1·3 units (*p* = 0.46) and a significant difference at three months of 3·1 units (*p* = 0.008, Supplement 1, Table X). In a *post hoc* analysis, an adjusted ordinal repeated measures analysis showed that PES was associated with improved (lower) DSRS scores, OR 0·22 (95% CI 0·13, 0·38; *p*<0·001).

## 4 Discussion

We assessed real-world usage of PES in 245 patients with neurogenic dysphagia from 14 hospitals in three European countries. The average age was 68 years and treatment was started at an average of 32 days after ictus. As compared with baseline, DSRS (and its three component subscales), FOIS (another measure of dysphagia severity) and instrumentally-assessed penetration/aspiration (PAS) all improved in each of the diagnostic groups as well as in supratentorial and infratentorial stroke, and in participants who could be decannulated as compared with those who could not following ventilation and tracheotomy. PES appeared to be most effective if started early and with short ventilation periods. Treatment was safe, user experience was positive and an average of only 1·1 catheters were used per participant.

Although PES has been shown to improve dysphagia after stroke [8,10,11], PHADER provides the first evidence that it may work in non-stroke causes of neurogenic dysphagia, including TBI and ventilator-related dysphagia such as critical illness polyneuropathy. Interestingly, the magnitude of improvement in DSRS, FOIS and PAS was less in TBI than other diagnostic groups and there are several possible explanations for this. First, the diffuse brain-damage present in TBI may mean that more of the swallowing circuitry is damaged and so is less amenable to recovery. Second, the same diffusivity of disease may mean that a single cycle of PES treatment is less likely to be effective. In the PHAST-TRAC trial in cannulated stroke patients, a second cycle of PES increased the number of participants who could be decannulated. Therefore, a second cycle with three more daily treatments might be important in TBI. Last, most TBI participants in PHADER were treated well beyond a month (median 73 days) whilst PHAST-TRAC suggested that delayed treatment (>28 days) with PES might be less effective than earlier treatment [11]. Hence, future studies may need to increase the number of PES treatments applied in those with more established dysphagia or with more diffuse neurological injury. A previously published phase II trial found that PES appeared to be beneficial in multiple sclerosis [12]. Treatment of ventilator-associated dysphagia is of relevance in patients with COVID-19 [16] and although patients with SARS-CoV-2 infection were not included in PHADER, PES has been used to treat dysphagia following ventilation for COVID-19 (personal communication: Marianna Traugott, Vienna Austria). Together, these data suggest that PES may be effective across a wide spectrum of causes of neurogenic dysphagia.

Two subgroup analyses were performed and are illuminating. In the first, DSRS fell in stroke patients irrespective of whether the lesion was supra- or infra-tentorial. The physiology of swallowing differs by anatomical region and so apparent benefit, irrespective of lesion location, suggests that PES works through multiple mechanisms. Stimulation of sensory afferents in the naso- and oropharyngeal mucosa, which feed the glossopharyngeal and vagus nerves, excite the nucleus tractus solitarius, other brainstem nuclei and onto subcortical and cortical areas [17]. Effects of this may be to increase corticobulbar and swallowing sensorimotor excitability. Additionally, there may be peripheral effects of PES, as seen with increases in salivary substance P in the period immediately after PES in stroke patients [18]. In ventilated patients requiring a tracheotomy, DSRS fell more in those who could be decannulated as compared with those who could not be over the three months of follow-up.

When comparing non-ventilated stroke patients in PHADER with sham-treated patients in the STEPS trial [9], DSRS at three months had improved more with PES than sham by a magnitude of 2·3 points. The difference between PHADER and STEPS appeared to be developing by the second week after treatment with a difference of over 1·0 point. Both differences equal or exceed the minimum clinical important difference for DSRS, which is 1 [6], and so can be considered to be clinically relevant. Two explanations may be relevant; first, clinical measures such as DSRS may lag in detecting improvements in dysphagia (see limitations below for an expanded discussion of this issue) and so assessment within two weeks may be too early. Second, the two studies assessed DSRS at different early timepoints, namely at 9 days in PHADER and 14 days in STEPS; hence, the longer time for natural recovery in STEPS will have benefitted these sham patients. In respect of the STEPS trial itself, patients had milder dysphagia and received a lower treatment current whilst sham patients received partial treatment, so future studies will need to focus on more severe dysphagia and with treatment involving higher treatment currents [9].

Our study has a number of strengths. First, it is the largest study of PES for the treatment of neurogenic dysphagia and is more than twice the size of the earlier STEPS (*n* = 126 [9]) and PHAST-TRAC (*n* = 69 [11]) phase III trials. Second, it provides a solid overview of PES treatment in the real-world treatment of patients with neurogenic dysphagia. Although most participants presented with a stroke, TBI or critical illness polyneuropathy, other diagnoses were also represented. Third, dysphagia severity was reduced in all groups suggesting that PES is effective in multiple different causes of neurogenic dysphagia. Last, sufficient patients were recruited to allow subgroups analyses, in particular allowing assessment of the effect of treatment in pre-defined subgroups including supra- and infra-tentorial stroke, and in post-ventilation participants who could, or not, be decannulated.

This study has several limitations. Most importantly, this was a single-arm study with no control/sham group. Following treatment, dysphagia severity improved as compared with baseline and this may have reflected, at least in part, natural recovery. In the STEPS trial, which involved stroke patients without ventilation, the mean DSRS in the sham group fell from 7.0 to 3.9 by 12 weeks, i.e. a total reduction of 3.1 points. This contrasts with a reduction of 6.7 points in the analogous group in PHADER over the same time period; hence, similar amounts of improvement may relate to each of natural improvement and PES. Further, natural recovery is unlikely to be the only explanation since treatment was typically started several weeks (months in the TBI group) after lesion/disease onset suggesting that dysphagia was relatively fixed at baseline; in spite of this, PES treatment was followed by a rapid improvement in DSRS, FOIS and PAS over a matter of days and weeks. Further, the reduction in DSRS seen in non-ventilated stroke participants at three months was greater than that seen in the sham group in STEPS. Comparisons of actively treated patients with a historical control group are difficult since patient characteristics and background treatment typically differ, as seen here where baseline dysphagia severity differed between PHADER and STEPS by almost 4 points. Nevertheless, regression analysis with adjustment for baseline detected a significant treatment benefit at three months (although baseline adjustment can inflate differences due to regression to the mean).

Second, although DSRS and FOIS are easy to measure, these dysphagia assessments may not be optimal when assessing rapid changes in swallowing performance, i.e. over the first few days. A primary reason is that DSRS is based on feeding routes, dietary and fluid consistency and supervision, and these may not be assessed frequently or changed by healthcare staff immediately on improvement. Hence, it is possible that faster rates of improvement would have been detected if dysphagia had been assessed more frequently, say daily. This potential delay contrasts with other outcome measures such as decannulation which responds rapidly, as seen in PHAST-TRAC [11]. Third, the size of non-stroke groups were relatively small for ventilator-related dysphagia and TBI (groups C and D), and especially other neurogenic dysphagia (group E); we removed the latter group since only 3 patients were recruited. The small size of this group reflects that the commonest causes of neurogenic dysphagia are stroke, ventilator-related and TBI, hence the predominance of these groups of patients. Fourth, much outcome data were incomplete reflecting the real-world registry design, hence not all secondary end-points could be adequately addressed. This is particularly relevant for the VFS and FEES examinations (and so the measurement of PAS) which were not mandated. Last, the rate of SAEs was relatively low which may be explained by known under-reporting of SAEs in open label studies. Nevertheless, the pattern of SAEs appears reasonable and consistent with previous PES trials and for the populations studied.

In patients with neurogenic dysphagia, PES was safe and associated with reduced dysphagia especially if treatment was started in the first month after ictus. In participants having VFS, PES was associated with less penetration/aspiration. These findings provide empirical support for using PES in patients with neurogenic dysphagia.

## Author contributions

Dr Bath had full access to all of the data in the study and takes responsibility for the integrity of the data and the accuracy of the data analysis.

*Concept and design*: Bath, Dziewas, Hamdy, Likar, Mistry, Saltuari

*Acquisition and interpretation of data*: Bath, Bocksrucker, de Broux, Dziewas, Everton, Haase, Hamdy, Herzog, Köstenberger, Ledl, Likar, Pucks-Faes, Ragab, Saltuari, Schüttler,

Suntrup-Krüger, Vosko, Walther, Warusevitane

*Drafting of the manuscript*: Bath, Dziewas, Hamdy

*Critical revision of the manuscript for important intellectual content*: Bocksrucker, de Broux, Everton, Haase, Herzog, Köstenberger, Ledl, Likar, Pucks-Faes, Ragab, Saltuari, Schüttler,

Suntrup-Krüger, Vosko, Walther, Warusevitane

*Statistical analyses (in addition to Cytel)*: Woodhouse

*Funding*: Phagenesis Ltd

*Administrative, technical, or material support*: Mistry, Raginis-Zborowska

*Supervision*: Bath, Hamdy, Dziewas

## Declaration of Competing Interest

Dr Bath is Stroke Association Professor of Stroke Medicine and is a National Institute for Health Research (NIHR) Senior Investigator; he reports receiving grant funding from the British Heart Foundation and Medical Research Council (MRC), was a co-Chief Investigator of PHADER and reports personal fees from Phagenesis, Diamedica, Moleac, Sanofi and Nestle.

Dr Suntrup-Krueger reports receiving grants from Else Kröner-Fresenius-Stiftung and German Research Foundation (DFG).

Dr Dziewas was a co-Chief Investigator of PHADER and reports receiving honoraria/fees from Bayer, Boehringer Ingelheim, Daiichi Sankyo, Nestle, Olympus, Sanofi and Pfizer.

Dr Hamdy is Chief Scientific Officer of Phagenesis Ltd; he is a board director, holds shares in Phagenesis Ltd; he reports receiving grant funding from the MRC, NIHR and Wellcome Trust, in addition to receiving honoraria from Allergan Pharmaceuticals and Dr Falk; he is also a NICE MTAC committee member and reviews medical technologies for potential guidance for use in the NHS, UK.

Dr. Vosko reports receiving honoraria from Boehringer Ingelheim, Daiichi Sankyo and Ever Pharma.

Dr Raginis-Zborowska and Dr Mistry are employees of Phagenesis Ltd.

The remaining authors have no declarations.

## Funding

This work was supported by Phagenesis Ltd. Two employees managed much of the study and are authors of this article.

## Appendix Supplementary materials

Image, application 1

Image, application 2

Image, application 3

Image, application 4

## References

[1] Cohen D., Roffe C., Beavan J. (2016). Post-stroke dysphagia: a review and design considerations for future trials. Int J Stroke.

[2] Bath P.M., Lee H.S., Everton L.F (2018). Swallowing therapy for dysphagia in acute and subacute stroke. Cochrane Database Syst Rev.

[3] Hamdy S., Aziz Q., Rothwell J.C., Hobson A., Thompson D.G (1998). Sensorimotor modulation of human cortical swallowing pathways. J Physiol.

[4] Jayasekeran V., Singh S., Tyrrell P. (2010). Adjunctive functional pharyngeal electrical stimulation reverses swallowing disability after brain lesions. Gastroenterology.

[5] Vasant D.H., Michou E., O'Leary N. (2016). Pharyngeal electrical stimulation in dysphagia poststroke: a prospective, randomized single-blinded interventional study. Neurorehabil Neural Repair.

[6] Everton L.F., Benfield J.K., Hedstrom A. (2020). Psychometric assessment and validation of the dysphagia severity rating scale in stroke patients. Sci Rep.

[7] Rosenbek J., Robbins J., Roecker E., Coyle J., Wood J (1996). A penetration-aspiration scale. Dysphagia.

[8] Scutt P., Lee H.S., Hamdy S., Bath P.M (2015). Pharyngeal electrical stimulation for treatment of poststroke dysphagia: individual patient data meta-analysis of randomised controlled trials. Stroke Res Treat.

[9] Bath P., Scutt P., Love J. (2016). Pharyngeal electrical stimulation for treatment of dysphagia in subacute stroke: a randomized controlled trial. Stroke.

[10] Suntrup S., Marian T., Schröder J.B. (2015). Electrical pharyngeal stimulation for dysphagia treatment in tracheotomized stroke patients: a randomized controlled trial. Intens Care Med.

[11] Dziewas R., Stellato R., van der Tweel I. (2018). Pharyngeal electrical stimulation for early decannulation in tracheotomised patients with neurogenic dysphagia after stroke (PHAST-TRAC): a prospective, single-blinded, randomised trial. Lancet Neurol.

[12] Restivo D., Casabona A., Centonze D., Marchese-Ragona R., Maimone D., Pavone A (2013). Pharyngeal electrical stimulation for dysphagia associated with multiple sclerosis: a pilot study. Brain Stimul.

[13] Crary M.A., Mann G.D., Groher M.E (2005). Initial psychometric assessment of a functional oral intake scale for dysphagia in stroke patients. Arch Phys Med Rehabil.

[14] Woodhouse L.J., Scutt P., Hamdy S. (2017). Route of feeding as a proxy for dysphagia after stroke and the effect of transdermal glyceryl trinitrate: data from the efficacy of nitric oxide in stroke randomised controlled Trial. Transl Stroke Res.

[15] Koestenberger M., Neuwersch S., Hoefner E. (2019). A Pilot study of pharyngeal electrical stimulation for orally intubated ICU patients with dysphagia. Neurocrit Care.

[16] Dziewas R., Warnecke T., Zürcher P., Schefold J.C (2020). Dysphagia in COVID-19 -multilevel damage to the swallowing network?. Eur J Neurol: Off J Eur Federat Neurol Soc.

[17] Restivo D.A., Hamdy S. (2018). Pharyngeal electrical stimulation device for the treatment of neurogenic dysphagia: technology update. Med Dev.

[18] Muhle P., Suntrup-Krueger S., Bittner S. (2017). Increase of substance P concentration in saliva after pharyngeal electrical stimulation in severely dysphagic stroke patients - an indicator of decannulation success?. Neurosignals.

