# Supplementary material for: Pharyngeal electrical stimulation for neurogenic dysphagia following stroke, traumatic brain injury or other causes: Main results from the PHADER cohort study
Source: eClinicalMedicine. 2020 Nov 10;28:100608. doi: 10.1016/j.eclinm.2020.100608 (PMC7700977; doi:10.1016/j.eclinm.2020.100608)

SUPPLEMENT

This appendix has been provided by the authors to give readers additional information about their work.

Pharyngeal electrical stimulation for neurogenic dysphagia following stroke, traumatic brain injury or other causes: main results from the PHADER cohort study

Philip M Bath,[1,2] DSc FMedSci; Lisa J Woodhouse,[1] MSc; Sonja Suntrup-Krueger,[3] MD PhD; Rudolf Likar,[4] MD PhD; Markus Koestenberger,[4] MD; Anushka Warusevitane,[5] MD PhD; Juergen Herzog,[6] MD; Michael Schuttler,[7] MD; Suzanne Ragab,[8] FRCP; Lisa Everton,[1,9] MPhil; Christian Ledl,[10] MA; Ernst Walther,[11] MD; Leopold Saltuari,[12] MD PhD; Elke Pucks-Faes,[12] MD; Christof Bocksrucker,[13] MD; Milan Vosko,[14] MD PhD; Johanna de Broux,[15] MD; Claus G. Haase,[16] MD PhD; Alicja Raginis-Zborowska,[17] PhD; Satish Mistry,[17] PhD; Shaheen Hamdy,‡[18] MD PhD; Rainer Dziewas,‡[3] MD PhD

1. Stroke Trials Unit, Division of Clinical Neuroscience, University of Nottingham, Nottingham NG5 1PB, United Kingdom
2. Stroke, Nottingham University Hospital NHS Trust, Nottingham NG5 1PB, United Kingdom
3. Department of Neurology, University Hospital Münster, Building A1, Albert-Schweitzer-Campus 1, 48149 Münster, Germany
4. Department of Anaesthesiology and Intensive Care Medicine, Klinikum Klagenfurt am Wörthersee, Klagenfurt am Wörthersee, Austria
5. Stroke Research, Royal Stoke University Hospital, University Hospitals of North Midlands NHS Trust, Parish Building, 1st Floor, Newcastle Road, Stoke-on-Trent, Staffordshire, ST4 6QG, United Kingdom
6. Clinic for Neurological rehabilitation and early rehabilitation, Schön Klinik München-Schwabing, Parzivalplatz 4, 80804 Munich, Germany
7. Centre of Neurology, Schön Klinik Bad Staffelstein, Am Kurpark 11, 96231 Bad Staffelstein, Germany
8. Department of Stroke, Philip Arnold Unit Ground Floor, Poole Hospital NHS Foundation Trust, Longfleet road, Poole, BH15 2JB, United Kingdom
9. Speech and Language Therapy, Nottinghamshire Healthcare NHS Foundation Trust, Nottingham NG3 6AA, United Kingdom
10. Specialist Clinic for Neurology, Neurological Rehabilitation and Alzheimer's Therapy, Schön Klinik Bad Aibling, Kolbermoorer Strasse 72, 83043 Bad Aibling, Germany
11. Clinic for Neurology and Neurorehabilitation, Schön Klinik Hamburg Eilbek, Hamburg, Germany
12. Department of Neurology, Ö. Landeskrankenhaus Hochzirl-Natters, Tiroler landesrankenanstalten GmbH. LkH Hochzirl, 6170 Zirl / Hochzirl, Austria
13. Department of Neurology, Konventhospital Barmherzige Brúder Linz, Seilerstätte 2, 4021 Linz, Austria
14. Department of Neurology 2, Kepler Universitätsklinikum, Med Campus III, Krankenhausstrasse 9, 4020 Linz, Austria
15. Clinic for Neurology, Alexianer Krefeld GmbH, Dießemer Bruch 81, 47805 Krefeld, Germany
16. Clinic for Neurology and Neurophysiology, Evangelische Kliniken Gelsenkirchen, Lehrkrankenhaus der Universität Essen-Duisburg Munckelstr. 27, 45879 Gelsenkirchen, Germany
17. Department for Clinical Research, Phagenesis Limited, Manchester M15 6SE, United Kingdom
18. Centre for Gastrointestinal Sciences, Faculty of Biology, Medicine and Health, University of Manchester and the Manchester Academic Health Sciences Centre, Manchester M6 8HD, United Kingdom

‡ Joint senior authors

Table of contents

Sections	Page
**Committees and Principal Investigators of the PHADER Study**	
Scientific, Design and Publication Committee	3
PHADER Investigators	3
Clinical Research Organisation	3
Statistical analysis	3
**Supplementary Text**	
Inclusion criteria	4
Exclusion criteria	4
Approvals	4
Intervention	4
Outcomes	4
Statistical analysis plan	5
Serious adverse events	5
References	5
Supplementary Tables	6
Supplementary Figures	17

Committees and Principal Investigators

Scientific, Design and Publication Committee
- Philip Bath, University of Nottingham, Nottingham, United Kingdom
- Rainer Dziewas, University Hospital Münster, Münster, Germany
- Rudolf Likar, Department of Anaesthesiology and Intensive Care Medicine, Klinikum Klagenfurt am Wörthersee, Klagenfurt am Wörthersee, Austria
- Shaheen Hamdy (Chair), University of Manchester and the Manchester Academic Health Sciences Centre (MAHSC), Manchester, United Kingdom
- Satish Mistry, Department for Clinical Research, Phagenesis Limited, Manchester, United Kingdom

PHADER Investigators
Arranged by Country then number of recruitments: Investigator, Site (Number of patients consented), Study Team

Austria:
- Rudolf Likar, Klagenfurt (57), Markus Koestenberger, Elmar Hoefner, Petra Jan, Helmut Weissmann, Verena Gassner, Marlies Janz, Brigitte Trummer, Susanne Demschar, Alexandra Kusej
- Leopold Saltuari, Hochzirl (11), Elke Pucks-Faes, Heinrich Matzak, Gregor Nawratil
- Christof Bocksrucker, Konventhospital Barmherzige Brúder Linz (10), Michaela Bachleitner, Berta Waldenberger, Sandra Grüllenberger
- Milan Vosko, Kepler Universitätsklinikum Linz (8), Jorg Balzar, Cornelia Brunner, Sandra Rathmaier

Germany:
- Rainer Dziewas, Münster (60), Dorothee Schulte-To-Bühne, Sonja Suntrup-Krueger, N. Gaubys, Illona Bredebusch
- Juergen Herzog, Munich-Schwabing (15), Christoph B. Lücking, Sina Peter, Edith Wagner-Sonntag, Sophia Fuss
- Michael Schuttler, Bad Staffelstein (14), Friedrich von Roasen, Jelena Pusica, Marion Mandl, Ari Ali, Christine Kasimir, Sina Mansoorian
- Christian Ledl, Bad Aibling (12), Friedemann Muller, Barbara Schäpers, Julia Zäch, Dizona Begu Musliu
- Ernst Walther, Hamburg (11), Larissa Fournier-Kouser, Caroline Köhn, Marco Gerschke, Annette Förster
- Johanna de Broux, Krefeld (7), Friedrich von Giesen, Nicole Hermanns, Bettina Dohmen, Rebecca Hillen Julia Schroers
- Claus G. Haase, Gelsenkirchen (6), Christoph Meyer, Erich Sigges, Nina Anton, Andrea Schindler, Brigitte Sundermann

United Kingdom
- Anushka Warusevitane, Royal Stoke (15), Holly Maguire, Kay Finney, Racquel Carpio, Stephanie Stevens, Joanne Hiden, Adrian Butler, Susan Lyjko, Adrian Barry, Jeanette Grocott, Jennifer Bee, Alda Remegoso, Nenette Abano, Resti Varquez, Jane Guga, Chelsea Causley, Andrew Moores, Hayley Denic, Francis Alipo, Leanne Dean
- Philip Bath, Nottingham (13), Amanda Buck, Carla Richardson, Ashana Tittle, Rekha Keshvara, Judith Clarke, Nicola Gilzeane, Lisa Everton, Caroline Appleby, Janet Tomlinson, Gwen Wilkes, Lauren Ryan, Lisa Vaughton, Jason Appleton, Zhe Kang Law
- Suzanne Ragab, Poole (12), Deborah Broadbent, Alice Gregan, Judith Dube, Jacqui Leggett, Beverley Wadams, Natasha Ross, Laura Gleave, Eleanie Chalmers

Clinical Research Organisation
- Fakkel CRO (Landen, Belgium): Jaak Minten (Director), Pellenstraat 7, 3400 Landen, Belgium

Statistical Analysis
- Cytel Inc. (Cambridge, USA): Global Headquarters, 675 Massachusetts Ave, Cambridge, MA 02139 USA

SUPPLEMENTARY TEXT

Inclusion criteria

Patients were eligible for study participation if they:

1. Adults of 18 years or older;
2. Suspected to have oropharyngeal dysphagia with a DSRS score of 6 or higher; or (when eating independency would be jeopardised by partial or total paralysis of upper extremities and when DSRS-assessment becomes invalidated) have a FOIS-score equal to or lower than 5; or (when no oral food intake is possible and DSRS score is 12/FOIS score is 1) have a PAS-score of 4 or higher;
3. Have neurogenic dysphagia comprising one of 5 groups:
    a) As a result of stroke but not requiring mechanical ventilation or tracheotomy;
    b) As a result of stroke but requiring mechanical ventilation or tracheotomy;
    c) Associated with mechanical ventilation but not related to stroke or traumatic brain (TBI);
    d) As a result of TBI or spinal cord injury with or without the need for mechanical ventilation or tracheotomy;
    e) Any other cause not associated with any element of groups a-d with or without the need for mechanical ventilation or a tracheotomy
4. Have given written consent, or proxy consent given by a legal representative;
5. No exclusion criteria present.

Exclusion Criteria

Patients are excluded from study participation if they;

1. Have an undefined date of medical event causing the dysphagia; or
2. Suffer from non-neurogenic dysphagia (e.g. cancer); or
3. Participate in any other study potentially influencing the outcome of PES, both medicinal or medical device product related and for which the patient signed a consent form for his/her study participation; or
4. Receive or have received within one month prior to the intended PES treatment any other type of standard cranial or percutaneous electrical stimulation therapy to treat dysphagia; or
5. Have a cardiac pacemaker or a cardioverter defibrillator implanted unless the device can be switched off completely at the time of treatment delivery; or
6. Have experienced an oesophageal perforation, or have an oesophageal stricture or pouch; or
7. Have an unstable cardiopulmonary status; or
8. Receive continuous oxygen treatment or have the equipment for such treatment permanently in place preventing the positioning of the Phagenyx Catheter (this does not exclude patients who are intubated or have a tracheotomy where an inflated balloon creates a firm barrier between the space where oxygen might be present (trachea/lungs) and the space where the electrical stimuli are delivered (oropharynx), or patients that can have the oxygen treatment temporarily stopped and equipment removed during PES<treatment); or
9. Are pregnant or nursing women; or
10. Require emergency treatment, preventing appropriate conduct of the subject informed consent process; or
11. Have a life expectancy less than the duration of the patient's follow up period, i.e. less than three months.

Approvals

In the UK, the study was also approved by hospital Research & Innovation Departments and study procedures were delivered by National Institute of Health Research (NIHR) Clinical Research network nurses/coordinators. Research Ethics Committee approval was obtained in all jurisdictions.

Intervention

During the course of this study, an upgraded version of the treatment catheter was introduced, this being more pliable and so reducing time to insertion; this catheter had exactly the same stimulation properties as the original catheter.

In patients not able to communicate verbally, sensory and tolerance thresholds were assessed by carefully observing the patient's reaction and vegetative response (especially heart rate and blood pressure) to increases in stimulation intensity.

Outcomes

The DSRS [1-3] is a validated measure of the severity of dysphagia impairment and comprises three subscales (fluids, diet, supervision) which are then summed; subscale scores range from 0 (normal fluids and diet, eating independently) to 4 (no oral fluids, diet or feeding) thereby giving a total score that ranges from 0 (best) to 12 (worst).

DSRS was chosen as the primary outcome since it is quick and easy to measure in all patients in contrast to videofluoroscopic PAS which is not used routinely in most patients and is not readily available in many centres.

Statistical analysis plan
Some intentions in the SAP will be reported later, e.g. health economics; others will not be performed as per section 5 of the SAP.

Serious adverse events
In the five patients with cardiac arrest, one event occurred after the first treatment but was thought to be unrelated due to its timing in the early morning. The other four cardiac arrests occurred at 4, 37, 47 and 61 days after treatment commenced.

References
1. Jayasekeran V, Singh S, Tyrrell P, et al. Adjunctive functional pharyngeal electrical stimulation reverses swallowing disability after brain lesions. *Gastroenterology* 2010; **138**(5): 1737-46.
2. Scutt P, Lee HS, Hamdy S, Bath PM. Pharyngeal Electrical Stimulation for Treatment of Poststroke Dysphagia: Individual Patient Data Meta-Analysis of Randomised Controlled Trials. *Stroke Res Treat* 2015; **2015**: 429053.
3. Everton LF, Benfield J, Hedstrom A, et al. Psychometric assessment and validation of the dysphagia severity rating scale in stroke patients *Scientific Reports* 2020; **In press**.
4. Dziewas R, Stellato R, van der Tweel I, et al. Pharyngeal electrical stimulation for early decannulation in tracheotomised patients with neurogenic dysphagia after stroke (PHAST-TRAC): a prospective, single-blinded, randomised trial. *Lancet Neurol* 2018.
5. Bath P, Scutt P, Love J, et al. Pharyngeal Electrical Stimulation for Treatment of Dysphagia in Subacute Stroke: A Randomized Controlled Trial. *Stroke* 2016; **47**(6): 1562-70.

Supplementary Table I. Events causing dysphagia in neurogenic dysphagia ordered by frequency in groups C and E (*post hoc* addition).

Group	Event	N
C. Ventilated	Critical illness polyneuropathy	15
	Hypoxia	3
	Seizures	3
	Encephalitis	2
	Guillain-Barre	2
	Meningitis	2
	Tumour	2
	Brain abscess	1
	Cavernoma	1
	Cerebral oedema	1
	Encephalopathy	1
	Multiple sclerosis	1
	Neurosarcoidosis	1
E. Non-ventilated	Tumour	2
	Spinal fusion surgery	1

Supplementary Table II. User experience of pharyngeal catheter. Data are number, mean (standard deviation); comparisons by one-way analysis of variance.

	N	All	Stroke, not ventilated	Stroke, ventilated	Ventilator-related [a]	TBI	Other	p-value
No of patients		239	79	98	35	24	3	
Catheter insertion time (min)	213	11·6 (8·8)	11·6 (9·8)	11·6 (9·1)	11·2 (6·9)	12·5 (7·2)	10·0 (5·0)	0·96
Ease of [b]								
Insertion of Catheter	209	5·1 (1·9)	5·4 (1·8)	4·9 (2·0)	5·1 (1·7)	4·7 (2·0)	6·0 (1·0)	0·30
Use of the guidewire	35	6·5 (0·7)	6·8 (0·4)	6·3 (0·7)	6·4 (0·9)	6·3 (1·0)	6·0 (-)	0·32
Use of the oral guide	36	5·7 (1·9)	6·3 (0·8)	5·8 (1·9)	5·0 (2·5)	6·5 (0·6)	1·0 (-)	0·38
Number of catheters used	91	1·1 (0·2)	1·0 (0·2)	1·1 (0·2)	1·0 (0·0)	1·1 (0·3)	1·0 (0·0)	0·57

[a] Not stroke or TBI
[b] Ease of use scored as: 1 = very difficult, to 7 = very easy [4]

Supplementary Table III. Pharyngeal electrical stimulation levels. Data are mean (standard deviation); comparison of groups by one-way analysis of variance.

Current (mA)	Day	All	Stroke, not ventilated	Stroke, ventilated	Ventilator-related [a]	TBI	Other	p-value
N		239	79	98	35	24	3	
Threshold	1	15·1 (8·0)	14·0 (9·3)	15·9 (7·9)	15·3 (6·5)	15·1 (5·2)	13·7 (7·0)	0·48
	2	15·3 (7·7)	14·2 (8·1)	15·8 (8·0)	15·7 (7·0)	15·9 (6·3)	15·7 (3·1)	0·52
	3	15·3 (7·9)	14·0 (7·6)	16·2 (8·0)	16·7 (8·6)	14·3 (6·6)	12·0 (2·0)	0·19
Tolerance	1	32·8 (11·5)	27·3 (11·0)	36·3 (11·2)	33·9 (10·9)	34·6 (9·4)	34·3 (14·0)	<0·001
	2	33·2 (11·5)	27·9 (12·0)	36·1 (10·7)	34·2 (10·1)	35·5 (9·6)	34·3 (13·4)	<0·001
	3	33·6 (11·4)	28·6 (11·8)	37·3 (10·1)	33·1 (11·0)	33·9 (9·7)	39·3 (18·5)	<0·001
Stimulation	1	28·1 (10·2)	23·8 (10·0)	30·9 (10·2)	28·8 (9·5)	29·5 (7·9)	28·7 (13·1)	<0·001
	2	28·6 (10·2)	24·4 (10·6)	31·2 (9·6)	28·8 (9·9)	30·8 (8·1)	28·7 (12·1)	<0·001
	3	28·8 (9·9)	24·5 (10·2)	31·9 (8·9)	29·4 (9·8)	28·5 (8·1)	32·7 (14·4)	<0·001

TBI: traumatic brain injury
[a] Not stroke or TBI

Supplementary Table IV. Dysphagia severity rating scale (DSRS) subscales (fluids, diet, supervision) by index group and timing. Data are number of participants, mean (standard deviation) or mean difference (95% confidence intervals); comparison of groups by one-way analysis of variance, and day 92 versus baseline by unpaired t-test.

Group	All	Stroke, not ventilated	Stroke, ventilated	Ventilator-related [a]	TBI	P-value
N	239	79	98	35	24	
DSRS Fluids						
Baseline	236, 3·8 (0·7)	79, 3·6 (0·9)	98, 3·9 (0·5)	35, 4·0 (0·2)	24, 3·8 (0·7)	0·014
Day 5	229,3·5 (1·1)	74, 3·3 (1·3)	97, 3·5 (1·1)	35, 3·5 (1·0)	23, 3·6 (1·1)	
Day 9	224, 2·7 (1·6)	70, 2·5 (1·7)	97, 2·8 (1·6)	35, 2·7 (1·5)	22, 3·3 (1·4)	
Day 92	174, 1·6 (1·7)	46, 1·2 (1·5)	78, 1·6 (1·8)	30, 1·7 (1·8)	20, 2·1 (1·8)	0·29
DIM (unpaired)	-2·2 (-2·5, -2) [b]	-2·4 (-2·9, -1·9) [b]	-2·3 (-2·7, -1·9) [b]	-2·2 (-2·9, -1·6) [b]	-1·7 (-2·6, -0·8) [b]	0·53
DSRS Diet						
Baseline	236, 3·8 (0·5)	79, 3·7 (0·7)	98, 3·9 (0·3)	35, 4·0 (0·2)	24, 3·8 (0·6)	0·006
Day 5	229, 3·6 (0·8)	74, 3·5 (0·8)	97, 3·7 (0·7)	35, 3·6 (0·9)	23, 3·8 (0·6)	
Day 9	224, 3·1 (1·2)	70, 2·9 (1·2)	97, 3·1 (1·2)	35, 2·9 (1·4)	22, 3·6 (0·8)	
Day 92	174, 1·8 (1·7)	46, 1·6 (1·5)	78, 1·8 (1·7)	30, 1·8 (1·8)	20, 2·5 (1·6)	0·33
DIM (unpaired)	-2·0 (-2·3, -1·8) [b]	-2·1 (-2·5, -1·6) [b]	-2·2 (-2·6, -1·8) [b]	-2·2 (-2·9, -1·5) [b]	-1·3 (-2·1, -0·5) [b]	0·20
DSRS Supervision						
Baseline	236, 3·8 (0·7)	79, 3·6 (1·0)	98, 3·9 (0·4)	35, 4·0 (0·2)	24, 3·8 (0·7)	0·003
Day 5	229, 3·5 (1·0)	74, 3·2 (1·1)	97, 3·6 (0·9)	35, 3·7 (0·8)	23, 3·7 (0·9)	
Day 9	224, 2·9 (1·4)	70, 2·4 (1·5)	97, 3·1 (1·3)	35, 2·8 (1·4)	22, 3·4 (1·0)	
Day 92	174, 1·8 (1·7)	46, 1·3 (1·4)	78, 1·8 (1·7)	30, 1·8 (1·8)	20, 2·3 (1·6)	0·17
DIM (unpaired)	-2·0 (-2·3, -1·8) [b]	-2·2 (-2·7, -1·7) [b]	-2·1 (-2·5, -1·7) [b]	-2·2 (-2·9, -1·5) [b]	-1·5 (-2·2, -0·7) [b]	0·29

DIM: difference in means; TBI: traumatic brain injury
[a] Not stroke or TBI
[b] p<0·001

Supplementary Table V. Baseline characteristics by location of stroke (groups A and B): supratentorial or infratentorial. Data are number (%), median [interquartile range] or mean (standard deviation); comparison of groups by Chi-square test, Kruskal-Wallis test or one-way analysis of variance.

Stroke location	N	All	Supratentorial	Infratentorial	p-value
N		182	151	31	
Age	182	69·7 (13·4)	70·3 (13·5)	66·8 (12·9)	0·20
Sex, male (%)	182	130 (71·4)	107 (70·9)	23 (74·2)	0·71
OTT (days)	175	23·0 [31·0]	24·0 [32·0]	14·0 [32·5]	0·077
Ventilated (group B, %)	182	98 (53·8)	83 (55·0)	15 (48·4)	0·50
Feeding status	182				0·65
Oral, normal		0 (0·0)	0 (0·0)	0 (0·0)	
Oral, supervised		5 (2·7)	4 (2·6)	1 (3·2)	
Oral, supported		4 (2·2)	3 (2·0)	1 (3·2)	
NGT or NJT		120 (65·9)	103 (68·2)	17 (54·8)	
PEG or RIG		46 (25·3)	35 (23·2)	11 (35·5)	
Other		7 (3·8)	6 (4·0)	1 (3·2)	
GCS (/15)	136	13·2 (2·4)	13·1 (2·5)	13·8 (1·7)	0·22
NIHSS (/42)	150	12·0 (7·3)	12·5 (7·4)	9·4 (6·6)	0·052
mRS (/6)	168	5·0 [1·0]	5·0 [1·0]	5·0 [1·0]	0·79
Stroke, ischaemic	182	152 (83·5)	125 (82·8)	27 (87·1)	0·56
Lesion location	182				<0·001
Right		59 (32·4)	59 (39·1)	0 (0·0)	
Left		75 (41·2)	75 (49·7)	0 (0·0)	
Bilateral		17 (9·3)	17 (11·3)	0 (0·0)	
Infratentorial		31 (17·0)	0 (0·0)	31 (100)	
Acute treatments					
Thrombolysis	153	50 (32·7)	43 (34·1)	7 (25·9)	0·41
Mechanical thrombectomy	66	21 (31·8)	18 (33·3)	3 (25·0)	0·58
Surgery	62	21 (33·9)	19 (38·0)	2 (16·7)	0·16
Tracheal cannula	182	59 (32·4)	53 (35·1)	6 (19·4)	0·088
Oxygen use in ICU	177	63 (35·6)	50 (34·2)	13 (41·9)	0·42
Ventilation (days)	86	18·5 [17·0]	19·0 [16·0]	14·5 [25·0]	0·43
Dysphagia assessment	181				0·71
Bedside		40 (22·1)	33 (21·9)	7 (23·3)	
VFS		3 (1·7)	3 (2·0)	0 (0·0)	
FEES		131 (72·4)	110 (72·8)	21 (70·0)	
VFS + FEES		7 (3·9)	5 (3·3)	2 (6·7)	
Stimulation, 3 day mean (mA)					
Threshold	175	15·1 (7·8)	14·1 (6·0)	20·4 (12·4)	<.001
Tolerance	175	32·6 (11·0)	31·9 (10·8)	36·1 (11·5)	0·059
Stimulation	176	28·1 (9·8)	27·3 (9·3)	32·3 (11·0)	0·011

NGT: nasogastric tube; NJT: nasojejunal tube; OTT: onset to treatment; PEG: percutaneous gastrostomy tube; PES: pharyngeal electrical stimulation; RIG: radiographically inserted gastrostomy tube

Supplementary Table VI. Dysphagia severity rating scale (primary outcome), functional oral intake scale, penetration aspiration scale, and length of stay in hospital by location of stroke (groups A and B): supratentorial or infratentorial. Data are number (%), mean (standard deviation) or mean difference (95% confidence intervals); comparison of groups using multiple linear regression (adjusted for baseline value, age and time since ictus), Chi-Square test or Kruskal-Wallis test, and day 92 versus baseline by unpaired t-test.

Outcome	All	Supratentorial	Infratentorial	p
N	176	147	29	
DSRS (/12)				
Baseline	176, 11·3 (1·9)	147, 11·3 (1·8)	29, 11·4 (2·3)	0·79
Day 5	170, 10·4 (2·7)	143, 10·3 (2·6)	27, 10·7 (3·1)	
Day 9	166, 8·4 (3·9)	140, 8·4 (3·9)	26, 8·7 (4·2)	
Day 92	124, 4·8 (4·8)	109, 4·6 (4·7)	15, 6·1 (5·4)	0·28
DIM (unpaired)	-7·1 (-7·8, -6·4)[c]	-7·3 (-8·1, -6·6)[c]	-5·9 (-7·6, -4·2)[c]	*0·32*
FOIS (/7)				
Baseline	161, 1·4 (1·0)	135, 1·4 (0·9)	26, 1·5 (1·3)	0·77
Day 5	158, 1·9 (1·4)	133, 1·9 (1·4)	25, 1·8 (1·6)	
Day 9	156, 2·8 (1·9)	131, 2·8 (1·9)	25, 2·5 (1·9)	
Day 92	121, 4·4 (2·5)	106, 4·4 (2·5)	15, 3·8 (2·6)	0·36
DIM (unpaired)	3·3 (2·9, 3·6)[c]	3·5 (3·0, 3·9)[c]	2·5 (1·5, 3·5)[c]	0·25
PAS (/8)				
Baseline	95, 6·8 (1·6)	76, 7·0 (1·3)	19, 5·8 (2·1)	0·004
Day 5	58, 5·0 (2·4)	45, 5·2 (2·3)	13, 4·5 (2·8)	
Day 9	65, 4·2 (2·7)	54, 4·0 (2·7)	11, 4·7 (2·8)	
Day 92	41, 3·0 (2·5)	35, 2·9 (2·5)	6, 3·3 (2·5)	0·71
DIM (unpaired)	-4·4 (-5·2, -3·6)[c]	-4·8 (-5·7, -3·9)[c]	-2·9 (-5·0, -0·8)	0·49
Still cannulated (Group B only)				
Baseline	59 (100·0)	53 (100·0)	6 (100·0)	-
Day 5	41 (70·7)	37 (71·2)	4 (66·7)	0·82
Day 9	33 (56·9)	29 (55·8)	4 (66·7)	0·61
Day 92	24 (41·4)	20 (38·5)	4 (66·7)	0·18
Time intervals (days)				
Hospital stay	38·0 [54·5]	38·0 [54·0]	34·0 [56·0]	0·94
PES-discharge	36·0 [54·5]	36·0 [55·0]	32·0 [56·0]	0·94
Discharge disposition (%) (*post hoc*)				0·41
Home care	13 (11·6)	12 (12·5)	1 (6·3)	
Full-nursing care	35 (31·3)	30 (31·3)	5 (31·3)	
Assisted care	5 (4·5)	3 (3·1)	2 (12·5)	
Sub-acute care	9 (8·0)	9 (9·4)	0 (0·0)	
Acute care	29 (25·9)	25 (26·0)	4 (25·0)	
Death	21 (18·8)	17 (17·7)	4 (25·0)	

DIM: difference in means; DSRS: dysphagia severity rating scale; FOIS: functional oral intake scale; PAS; penetration aspiration scale; PES: pharyngeal electrical stimulation

[a] p<0·001

Supplementary Table VII. Baseline characteristics in ventilated/tracheostomised participants (groups B and C, and D if ventilated) who could be decannulated after treatment. Data are number (%), median [interquartile range] or mean (standard deviation); comparison of groups by Fisher's exact test, Kruskal-Wallis test or one-way analysis of variance.

	N	All	Decannulated	Not decannulated	p-value
N (%)		99	66 (66·7)	33 (33·3)	
Age	99	64·6 (13·6)	63·1 (14·9)	67·8 (10·0)	0·10
Sex, male (%)	99	72 (72·7)	48 (72·7)	24 (72·7)	1·00
OTT (days)	98	40·0 [44·0]	37·5 [33·0]	55·5 [80·0]	0·013
GCS (/15)	76	12·4 (3·2)	12·5 (3·4)	12·2 (2·7)	0·71
Feeding status (%)	99				0·16
Oral, normal		0 (0·0)	0 (0·0)	0 (0·0)	
Oral, supervised		0 (0·0)	0 (0·0)	0 (0·0)	
Oral, supported		0 (0·0)	0 (0·0)	0 (0·0)	
NGT or NJT		65 (65·7)	47 (71·2)	18 (54·5)	
PEG or RIG		33 (33·3)	18 (27·3)	15 (45·5)	
Other		1 (1·0)	1 (1·5)	0 (0·0)	
Duration of ventilation (days)	85	26·0 [20·0]	26·0 [19·5]	27·0 [22·0]	0·70
Baseline dysphagia assessment	99				0·10
Bedside		7 (7·1)	7 (10·6)	0 (0·0)	
VFS		1 (1·0)	0 (0·0)	1 (3·0)	
FEES		90 (90·9)	58 (87·9)	32 (97·0)	
VFS + FEES		1 (1·0)	1 (1·5)	0 (0·0)	
Stimulation, mean of 3 days (mA)	99				
Threshold		14·9 (6·6)	14·0 (5·8)	16·8 (7·8)	0·049
Tolerance		35·1 (9·8)	34·5 (9·8)	36·1 (9·7)	0·44
Stimulation		29·9 (8·8)	29·2 (8·8)	31·2 (8·9)	0·29
Stroke (group B) only					
NIHSS (/42)	48	13·2 (5·3)	12·6 (4·9)	14·4 (6·1)	0·26
mRS (/6)	61	5·0 [0·0]	5·0 [1·0]	5·0 [0·0]	0·12
Stroke, ischaemic (%)	60	47 (78·3)	34 (89·5)	13 (59·1)	0·006
Side of Lesion (%)	60				0·85
Right		22 (36·7)	15 (39·5)	7 (31·8)	
Left		25 (41·7)	16 (42·1)	9 (40·9)	
Bilateral		7 (11·7)	4 (10·5)	3 (13·6)	
Infratentorial		6 (10·0)	3 (7·9)	3 (13·6)	
Oxygen use (%)	93	39 (41·9)	24 (39·3)	15 (46·9)	0·48
Acute treatments (%)					
Thrombolysis	53	22 (41·5)	16 (50·0)	6 (28·6)	0·12
Mechanical thrombectomy	30	14 (46·7)	10 (52·6)	4 (36·4)	0·39
Surgery	29	16 (55·2)	10 (52·6)	6 (60·0)	0·71

NGT: nasogastric tube; NJT: nasojejunal tube; OTT: onset to treatment; mRS: modified Rankin Scale; PEG: percutaneous gastrostomy tube; RIG: radiographically inserted gastrostomy tube

Supplementary Table VIII. Dysphagia severity rating scale (primary outcome), functional oral intake scale, penetration aspiration score, and length of stay in hospital by decannulation status. Data are number of participants, mean (standard deviation) or mean difference. Comparison of groups by multiple linear regression (adjusted for baseline value, age and time since ictus), Chi-Square test or Kruskal-Wallis test, and day 92 versus baseline by unpaired t-test.

	All	Decannulated	Not decannulated	p
N (%)	99	66 (66·7)	33 (33·3)	
DSRS (/12), Primary Outcome				
Baseline	99, 11·7 (1·0)	66, 11·6 (1·2)	33, 11·9 (0·3)	0·163
Day 5	97, 11·1 (2·0)	66, 10·8 (2·3)	31, 11·8 (0·7)	
Day 9	97, 9·3 (3·7)	66, 8·3 (3·9)	31, 11·5 (1·5)	
Day 92	85, 6·0 (5·2)	58, 4·2 (4·7)	27, 10·0 (4·0)	<0·001
DIM (unpaired)	-5·9 (-6·8, -5·0)[a]	-7·5 (-8·6, -6·5)[a]	-2·1 (-3·2, -1·0)[a]	
FOIS (/7)				
Baseline	99, 1·2 (0·5)	66, 1·2 (0·6)	33, 1·1 (0·3)	0·253
Day 5	96, 1·5 (1·0)	66, 1·7 (1·2)	30, 1·3 (0·5)	
Day 9	97, 2·3 (1·7)	66, 2·8 (1·9)	31, 1·3 (0·5)	
Day 92	87, 3·9 (2·7)	59, 4·9 (2·5)	28, 2·0 (1·9)	<0·001
DIM (unpaired)	2·8 (2·4, 3·3)[a]	3·7 (3·1, 4·2)[a]	0·9 (0·4, 1·4)[a]	
PAS (/8)				
Baseline	52, 6·8 (1·7)	31, 6·6 (2·0)	21, 7·1 (1·1)	0·268
Day 5	28, 5·4 (2·4)	22, 5·1 (2·5)	6, 6·3 (1·9)	
Day 9	37, 4·7 (2·8)	24, 4·0 (2·9)	13, 5·9 (2·4)	
Day 92	29, 3·7 (2·8)	20, 2·8 (2·4)	9, 5·8 (2·7)	0·006
DIM (unpaired)	-3·5 (-4·4, -2·5)[a]	-4·2 (-5·4, -3·1)[a]	-1·6 (-3·0, -0·2)[b]	
Cannula present				
Baseline	99 (100·0)	66 (100·0)	33 (100·0)	-
Day 5	74 (75·5)	42 (63·6)	32 (100·0)	<0·001
Day 9	55 (56·7)	24 (36·4)	31 (100·0)	<0·001
Day 92	37 (38·1)	6 (9·1)	31 (100·0)	<0·001
Time intervals (days)				
Hospital stay	35·0 [55·0]	33·5 [50·0]	35·0 [56·0]	0·99
PES-discharge	32·0 [56·0]	31·5 [49·0]	32·0 [56·0]	0·40
Discharge Destination (%) (*post hoc*)				0·21
Home care	13 (22·4)	7 (17·9)	6 (31·6)	
Full-nursing care	23 (39·7)	18 (46·2)	5 (26·3)	
Assisted care	0 (0·0)	0 (0·0)	0 (0·0)	
Sub-acute care	4 (6·9)	1 (2·6)	3 (15·8)	
Acute care	12 (20·7)	9 (23·1)	3 (15·8)	
Death	6 (10·3)	4 (10·3)	2 (10·5)	

DIM: difference in means; DSRS: dysphagia severity rating scale; FOIS: functional oral intake scale; PAS; penetration aspiration scale; PES: pharyngeal electrical stimulation

[a] p<0·001
[b] p<0·05

Supplementary Table IX. Serious adverse events ordered by system. Data are number (%); comparison of groups by Chi-square test.

System	SAE Term	All	TTE [days]	Stroke, not ventilated	Stroke, ventilated	Ventilator-related [a]	TBI	Other	p
N		245		84	99	35	24	3	
Participants									
With SAE		60 (24·5)	25 [44]	31 (36·9)	21 (21·2)	6 (17·1)	2 (8·3)	0 (0·0)	0·008
Fatal SAE		29 (11·8)	34 [33]	16 (19·0)	9 (9·1)	2 (5·7)	2 (8·3)	0 (0·0)	0·095
Events									
SAE		74		37	28	7	2	0	-
Fatal SAE		30 (40·5)		17 (45·9)	9 (32·1)	2 (28·6)	2 (100·0)	0	-
Cardiac	Atrial fibrillation	2 (0·8)	36 [45]	2 (2·4)	0 (0·0)	0 (0·0)	0 (0·0)	0 (0·0)	0·29
	Cardiac arrest	5 (2·0)	38 [43]	2 (2·4)	1 (1·0)	2 (5·7)	0 (0·0)	0 (0·0)	0·34
	Cardiac failure	1 (0·4)	74 [0]	1 (1·2)	0 (0·0)	0 (0·0)	0 (0·0)	0 (0·0)	0·60
Gastrointestinal	Liver cancer	1 (0·4)	51 [0]	1 (1·2)	0 (0·0)	0 (0·0)	0 (0·0)	0 (0·0)	0·60
	Liver insufficiency	1 (0·4)	16 [0]	0 (0·0)	1 (1·0)	0 (0·0)	0 (0·0)	0 (0·0)	0·60
	Parotitis	1 (0·4)	-9 [0] [c]	1 (1·2)	0 (0·0)	0 (0·0)	0 (0·0)	0 (0·0)	0·60
	Peritonitis	1 (0·4)	22 [0]	1 (1·2)	0 (0·0)	0 (0·0)	0 (0·0)	0 (0·0)	0·60
Neurological	Brain Abscess	1 (0·4)	4 [0]	0 (0·0)	0 (0·0)	1 (1·0)	0 (0·0)	0 (0·0)	0·12
	Encephalomyelitis	1 (0·4)	93 [0]	0 (0·0)	1 (1·0)	0 (0·0)	0 (0·0)	0 (0·0)	0·69
	Hydrocephalus	1 (0·4)	64 [0]	0 (0·0)	1 (1·0)	0 (0·0)	0 (0·0)	0 (0·0)	0·69
	PRES	1 (0·4)	41 [0]	0 (0·0)	1 (1·0)	0 (0·0)	0 (0·0)	0 (0·0)	0·69
	Reduced consciousness	1 (0·4)	7 [0]	0 (0·0)	1 (1·0)	0 (0·0)	0 (0·0)	0 (0·0)	0·69
	Seizures	2 (0·8)	103 [39]	2 (2·4)	0 (0·0)	0 (0·0)	0 (0·0)	0 (0·0)	0·29
	Stroke	3 (1·2)	18 [30]	3 (3·6)	0 (0·0)	0 (0·0)	0 (0·0)	0 (0·0)	0·13
Other	Death, cause unknown	2 (0·8)	68 [97]	1 (1·2)	1 (1·0)	0 (0·0)	0 (0·0)	0 (0·0)	0·88
	Dehydration	1 (0·4)	66 [0]	0 (0·0)	1 (1·0)	0 (0·0)	0 (0·0)	0 (0·0)	0·69
	Infection/sepsis, other	3 (1·2)	21 [11]	1 (1·2)	2 (2·0)	0 (0·0)	0 (0·0)	0 (0·0)	0·75
	Multiple organ failure	1 (0·4)	60 [0]	0 (0·0)	0 (0·0)	1 (2·9)	0 (0·0)	0 (0·0)	0·12
	Wound healing disorder	1 (0·4)	51 [0]	0 (0·0)	1 (1·0)	0 (0·0)	0 (0·0)	0 (0·0)	0·69
Renal	Acute kidney injury	1 (0·4)	38 [0]	0 (0·0)	1 (1·0)	0 (0·0)	0 (0·0)	0 (0·0)	0·69
	Haematuria	1 (0·4)	78 [0]	1 (1·2)	0 (0·0)	0 (0·0)	0 (0·0)	0 (0·0)	0·60
	Urosepsis	2 (0·8)	54 [44]	0 [0·0]	2 [2·0]	0 [0·0]	0 [0·0]	0 [0·0]	0·41
Respiratory	Lung cancer	1 (0·4)	37 [0]	1 (1·2)	0 (0·0)	0 (0·0)	0 (0·0)	0 (0·0)	0·60
	Pneumonia/RTI	26 (10·6)	22 [35]	15 (17·9)	7 (7·1)	2 (5·7)	2 (8·3)	0 (0·0)	0·075
	Pneumonia/RTI [b]	1 (0·4)	2 [0]	0 (0·0)	1 (1·0)	0 (0·0)	0 (0·0)	0 (0·0)	0·69
	Respiratory failure	4 (1·6)	12 [33]	1 (1·2)	3 (3·0)	0 (0·0)	0 (0·0)	0 (0·0)	0·52
	Severe bronchitis	1 (0·4)	30 [0]	0 (0·0)	1 (1·0)	0 (0·0)	0 (0·0)	0 (0·0)	0·70
	Tracheal stenosis	1 (0·4)	34 [0]	0 (0·0)	1 (1·0)	0 (0·0)	0 (0·0)	0 (0·0)	0·70

Vascular	Fainting	1 (0·4)	87 [0]	1 (1·2)	0 (0·0)	0 (0·0)	0 (0·0)	0 (0·0)	0·60
	Peripheral vascular disease	1 (0·4)	15 [0]	1 (1·2)	0 (0·0)	0 (0·0)	0 (0·0)	0 (0·0)	0·60
	Pulmonary embolism	2 (0·8)	16 [2]	1 (1·2)	1 (1·0)	0 (0·0)	0 (0·0)	0 (0·0)	0·88

GCS: Glasgow coma scale; LRTI: lower respiratory tract infection; PRES: posterior reversible encephalopathy syndrome; RTI: respiratory tract infection/chest infection; SAE: serious adverse event; TBI: traumatic brain injury; TTE: time to event

[a] Not stroke or TBI [b] 1 case of chest sepsis "possibly-related" to catheter insertion [c] Started after consent/before treatment

Supplementary Table X. Baseline characteristics and comparison of 13-level dysphagia severity rating scale (primary outcome) in PHADER stroke (non-ventilated, Group A) versus STEPS sham [5] participants. Data are given for STEPS active group for information. Data are number, median [interquartile range], mean (standard deviation), difference in means, and 95% confidence intervals. Comparison of groups by Chi-square test, t-test (unpaired) or ordinal logistic regression adjusted for age, sex, NIHSS, mRS, stroke type and time from stroke onset to treatment.

	STEPS PES *ƒ* [5]	STEPS sham *ƒ* [5]	PHADER PES (A)	Difference	p
Number of participants	87	75	79		
Baseline					
Age (years)	74·0 (10·1) [n=85]	75·3 (12·5) [n=74]	73·6 (12·9) [n=79]	1·7 (-2·4, 5·8)	0·41
Sex, male (%)	47 (55·3) [n=85]	45 (60·8) [n=74]	54 (68·4) [n=79]	-7·5 (-22·7, 7·6)	0·33
Onset-treatment (days)	14·0 [10·5] [n=80]	16·0 [15·0] [n=66]	15·0 [24·0] [n=78]	-1·0 (-5·0, 3·0)	0·55
Ischaemic stroke (%)	64 (73·6) [n=87]	60 (80·0) [n=75]	74 (93·7) [n=79]	-13·7 (-24·2, -3·1)	0·012
mRS [/6]	4·0 [1·0] [n=82]	4·0 [1·0] [n=71]	4·0 [1·0] [n=69]	0·0 (0·0, 0·0)	0·99
NIHSS (/42)	9·6 (6·5) [n=81]	10·2 (6·2) [n=71]	10·6 (8·6) [n=73]	-0·4 (-2·9, 2·0)	0·73
NG/PEG tube fed (%)	55 (63·2) [n=87]	39 (52·0) [n=75]	66 (83·5) [n=79]	-31·5 (-45·5, -17·6)	<0·001
Stimulation current (mA)	14·5 (6·8) [n=77]	15·2 (7·6) [n=63]	24·3 (9·7) [n=79]	-9·1 (-12·1, -6·2)	<0·001
DSRS (/12)					
Baseline	8·0 [8·0] [n=84]	6·5 [7·0] [n=70]	12·0 [0·0] [n=79]	-5·0 (-5·0, -3·0)	<0·001
Adjusted OLR					
Day 9/14	4·0 [6·0] [n=72]	4·0 [4·0] [n=59]	9·0 [8·0] [n=70]	0·2 (0·1, 0·4)	<0·001
Day 92	1·0 [4·0] [n=60]	0·5 [3·0] [n=46]	2·5 [7·0] [n=46]	0·4 (0·2, 1·0)	0·056
Difference, Day 9/14 - 0	-1·5 [4·0] [n=72]	-2·0 [3·0] [n=59]	-1·0 [6·0] [n=70]	1·3 (0·7, 2·6)	0·46
Difference, Month 3 - 0	-4·0 [6·5] [n=60]	-5·0 [6·0] [n=46]	-6·0 [8·0] [n=46]	3·1 (1·4, 7·3)	0·008

DSRS: dysphagia severity rating scale; NG: nasogastric tube; NIHSS: National Institutes of Health stroke scale; PEG: percutaneous endoscopic gastrostomy: PES: pharyngeal electrical stimulation

ƒ Baseline characteristics, dysphagia severity and change in severity did not differ between STEPS active and sham groups

† Timing of first follow-up: PHADER day 9, STEPS day 14

‡ Threshold and tolerability were assessed in sham patients but 10 minutes of treatment was not delivered

A parallel analysis comparing PHADER PES group B with PHAST-TRAC (sham) was not performed since the design of the latter provided delayed/open-label PES to many sham patients.

Supplementary Figure I. Box and whisker plot by time of i) FOIS (left panel) and ii) PAS (right panel). Figures show 5th centile, 25th centile, box containing median (horizontal line) and mean (diamond), 75th centile and 95th centile at each timepoint.

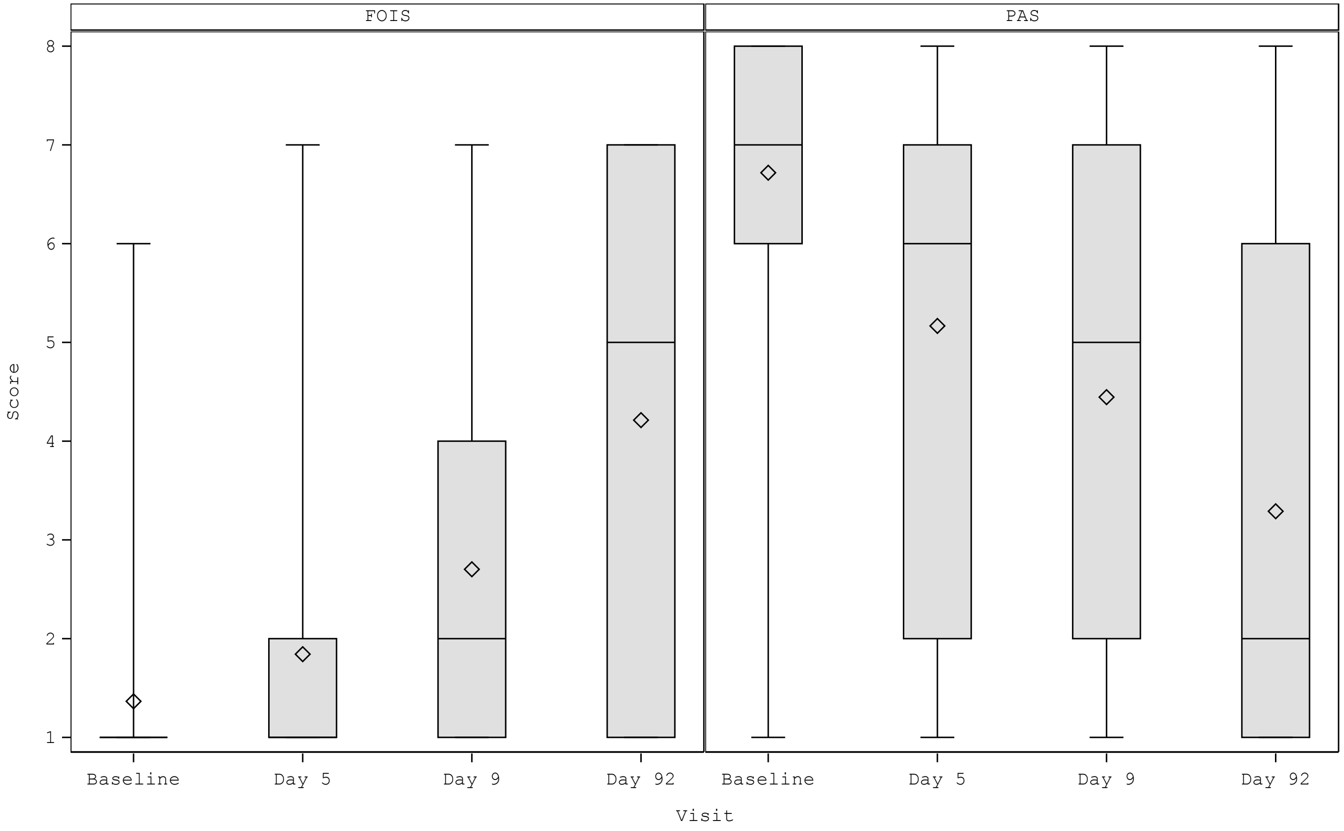

Supplementary Figure II. Line plot of DSRS and FOIS in i) Tracheotomised patients (groups B, C and some of D) and ii) Non-tracheotomised patients (groups A, some of D)

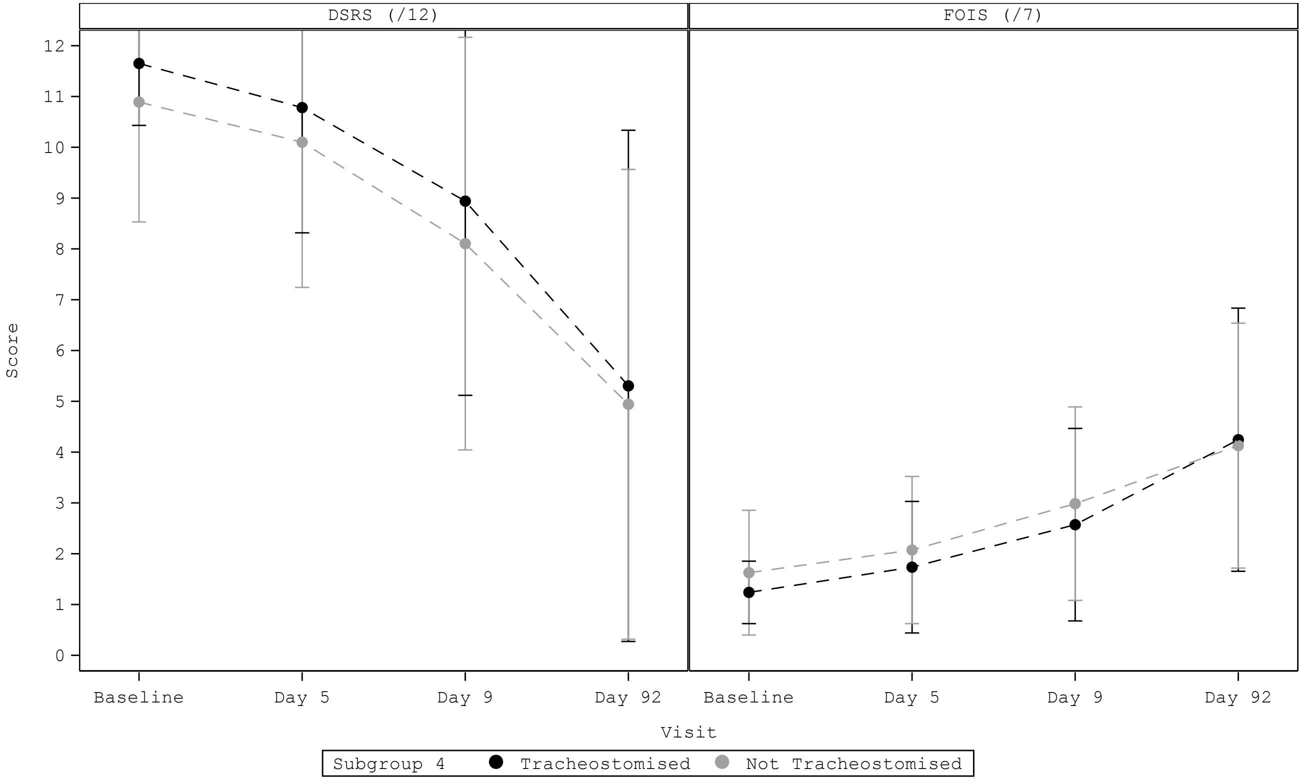

Supplementary Figure III. Cumulative plot of time to hospital discharge and/or re-start of oral feeding

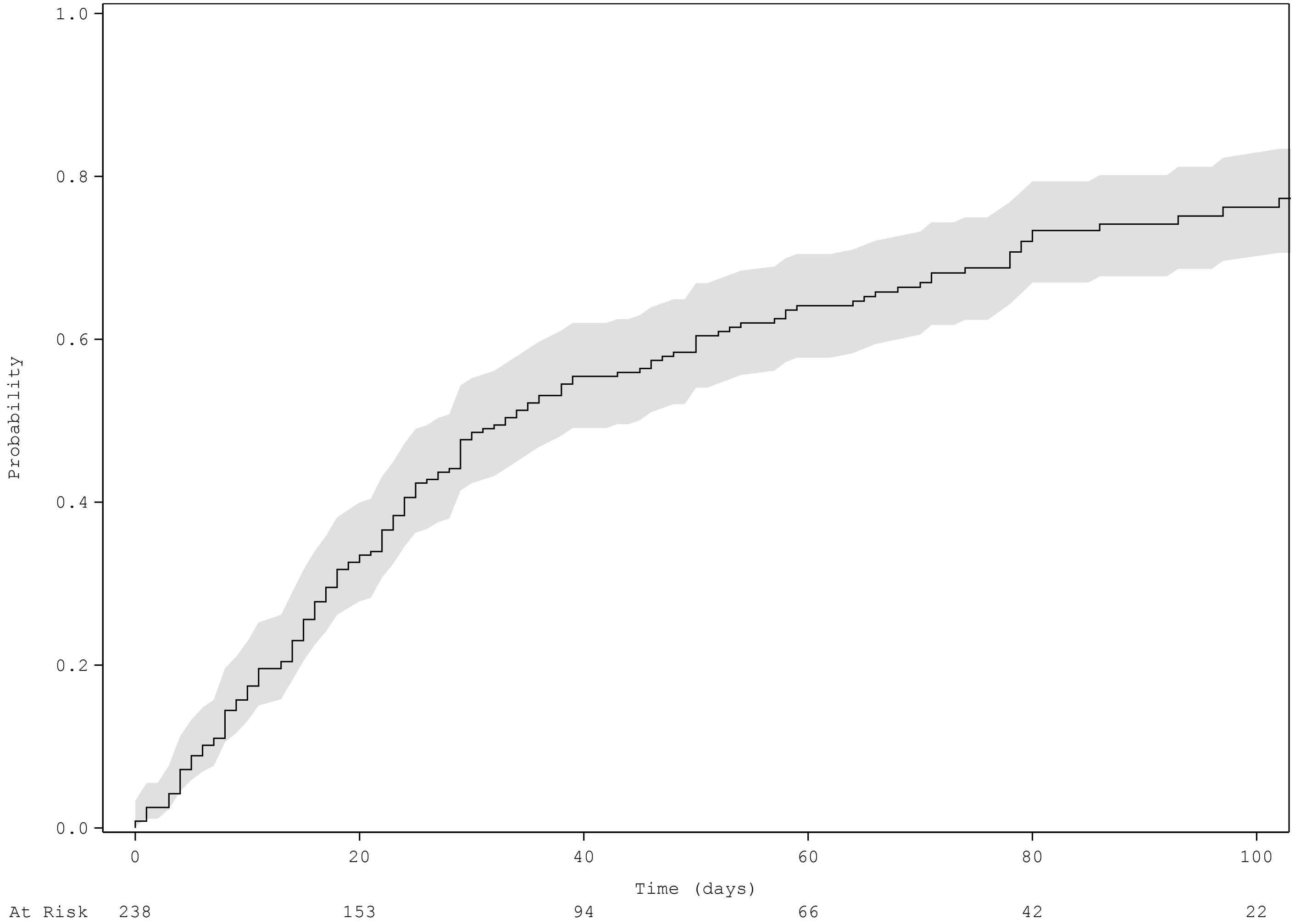

Supplementary Figure IV. Occurrence of serious adverse events by time (*post hoc*).

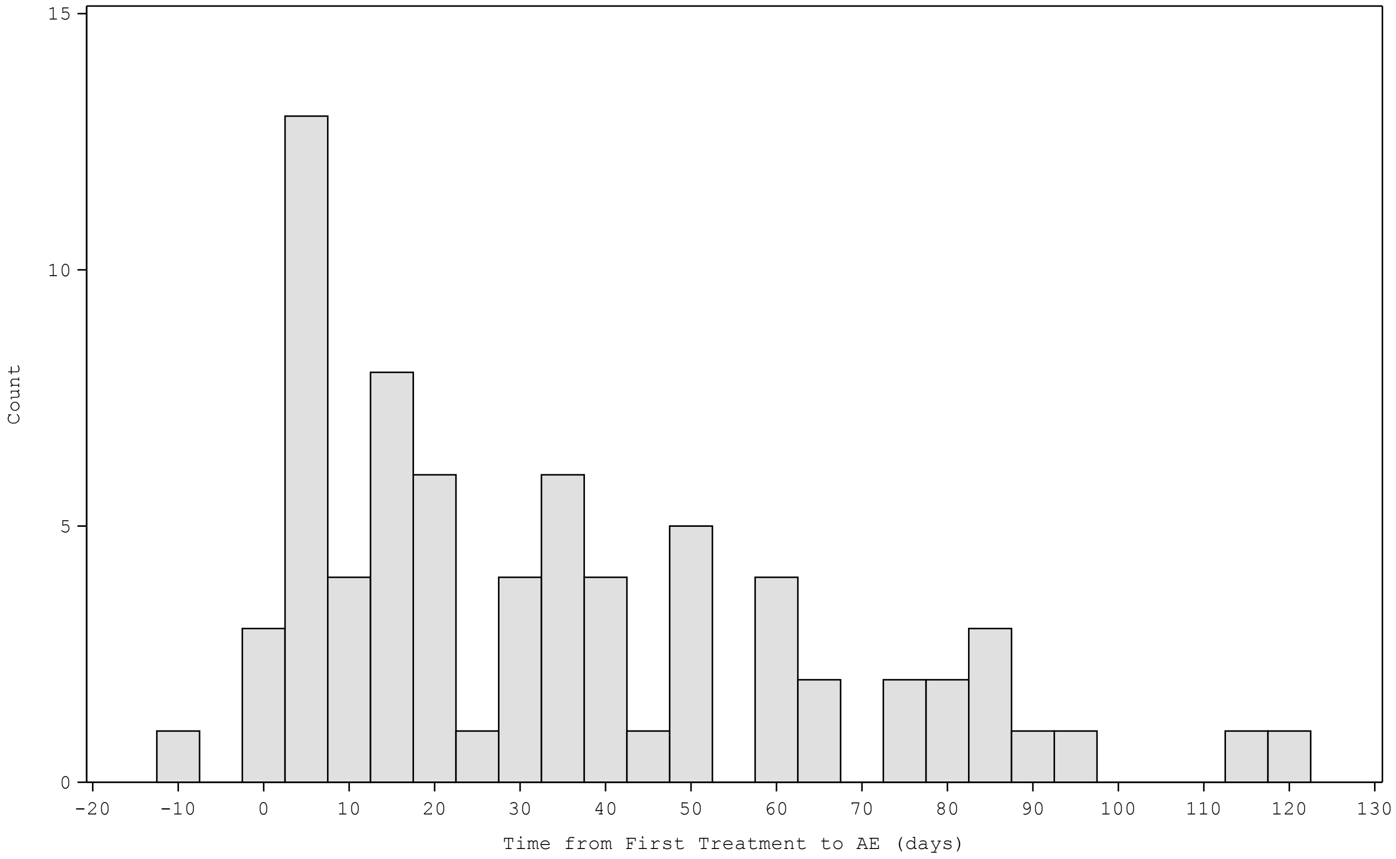

Supplement: Supplementary file 2 [file mmc2.pdf]
