# Supplementary material for: Pharyngeal electrical stimulation for neurogenic dysphagia following stroke, traumatic brain injury or other causes: Main results from the PHADER cohort study
Source: eClinicalMedicine. 2020 Nov 10;28:100608. doi: 10.1016/j.eclinm.2020.100608 (PMC7700977; doi:10.1016/j.eclinm.2020.100608)

**PHADER**
European Registry

**Strictly Confidential**

# A prospective, single-arm, observational clinical follow-up study on the application of PHAryngeal electrical stimulation for treatment of neurogenic Dysphagia: a European Registry (PHADER)

| | |
|---|---|
| **Study Number:** | AHE-02 |
| **CIP Version:** | 10 April 2015 |

**This clinical Investigation Plan has been authored by:**

| | |
|---|---|
| **Dr Jaak Minten, Ph.D.**<br>Clinical Research Director,<br>Phagenesis Limited<br>Date: | 10 Apr 2015 |
| **Dr Satish Mistry, Ph.D.**<br>Clinical Project Manager,<br>Phagenesis Limited<br>Date: | 10 APR 2015 |

**This clinical Investigation Plan has been read and approved by:**

| | |
|---|---|
| **Professor Shaheen Hamdy**<br>Co ordinating/Chief Investigator<br>Date: | 10/4/15 |
| **Mr Kieran Conlon**<br>Quality Assurance and Regulatory Affairs Manager,<br>Phagenesis Limited<br>Date: | 10/4/15 |

**This Clinical Investigation is being sponsored by:**

Phagenesis Limited, Unit 18, Enterprise House, Pencroft Way, Manchester Science Park, Manchester, United Kingdom. M15 6SE

PHAGENESIS®

## Principal Investigator Study Approval Page

**A prospective, single-arm, observational clinical follow-up study on the application of PHAryngeal electrical stimulation for treatment of neurogenic Dysphagia: a European Registry (PHADER)**

I, the undersigned, have read and understood the Clinical Investigation Plan specified above, and agree on the contents. The Clinical Investigation Plan and the Clinical Investigator Agreement will serve as a basis for cooperation in the Clinical Investigation.

| | INVESTIGATOR | CO-INVESTIGATOR |
|---|---|---|
| **Name:** | | |
| **Title:** | | |
| **Address:** | | |
| **Tel No.:** | | |
| **Fax No.:** | | |
| **E-mail:** | | |

| | | |
|---|---|---|
| **Signature:** | | |
| **Date of Signature:** | | |

# Table of Contents

1. List of Abbreviations and Definitions ..... 6
2. Statement of Conformity ..... 8
3. Synopsis ..... 9
4. Background and Rationale ..... 13
5. Device Used in the Clinical Investigation ..... 15
5.1. The Base Station, it's Stimulation Catheters and Electrical Stimulation ..... 15
5.2. Device Accountability ..... 16
5.3. Risks and Benefits of the Product and Clinical Investigation ..... 16
6. Objectives ..... 16
6.1. Objectives and Endpoints of the Post-Market Release Follow-Up Study. ..... 16
6.2. Hypothesis ..... 16
6.3. Primary Objective ..... 17
6.4. Secondary Objectives ..... 17
6.5. Primary Endpoint ..... 18
6.6. Secondary Endpoints ..... 18
7. Patient Selection ..... 19
7.1. Target Population ..... 19
7.2. Inclusion Criteria ..... 20
7.3. Exclusion Criteria ..... 20
7.5. Patient Enrolment and Withdrawal Criteria ..... 21
8. Study Design ..... 22
8.1. Investigational Sites ..... 22
8.2. Identification and Selection ..... 23
8.3. Ethics Committee Approval ..... 23
8.4. Site Activation ..... 24
8.5. Patient Discharge to Another Treatment Unit or to Home ..... 24
9. Investigation Procedures ..... 25
9.1. Informed Consent ..... 26
9.2. Patient Eligibility ..... 26
9.3. Assessment Prior to Treatment ..... 26

9.4. Treatment Procedure ..... 29
9.5. Follow-up Evaluations ..... 31
10. Adverse Events (AEs) ..... 33
11. Data Management ..... 34
12. Suspension, Termination and Close-out of Clinical Investigation ..... 36
13. Statistics and Data Analysis ..... 37
14. Reports and Publications ..... 38
14.1. Interim Report ..... 39
14.2. Final Report ..... 39
14.3. Publications and Publication Policy ..... 39
15. Ethical Considerations ..... 40
15.1. Declaration of Helsinki ..... 40
15.2. Ethics Committee Approval ..... 40
15.3. Informed Consent and Patient Information ..... 40
15.5. Patient Insurance – Indemnity / Patient Pay-Outs ..... 40
16. Good Clinical Practice (GCP) Compliance ..... 40
16.1. Professional Conduct of the Clinical Study ..... 40
16.2. Training Provided by Sponsor ..... 41
16.3. ISO 14155 (version 2011) ..... 41
17. References ..... 41
Appendices: ..... 42
A.1: Adverse Event (AE) definitions and handling ..... 43
A.2: Patient (Personal/Nominated Consultee) Information Sheet and Informed Consent (Declaration) Forms ..... 48
A.3: Investigator Agreement: Standard Template ..... 67
A.4: Evidence of Clinical Investigation Insurance ..... 68
A.5: Structure of Data Forms/Database ..... 69
A.6: Case Report Form Construction ..... 70
A.7: Patient Data Collection Flow Chart ..... 78
A.8: Patient Card ..... 79
A.9: Speech and Language Therapy Management ..... 80
B.1: Dysphagia Severity Rating Scale (DSRS) ..... 83

B.2: Functional Oral Intake Scale (FOIS) ........ 84
B.3: Eating Assessment Tool (EAT-10) ........ 85
B.4: Penetration and Aspiration Scale (PAS) ........ 86
B.5: Secretion Severity Rating Scale (SSS) ........ 87
B.6: Barthel Index (BI) ........ 88
B.7: Euro-Qol-5D (5 level answers) ........ 90
B.8: Dysphagia Handicap Index (DHI) ........ 92
B.9: Modified Ranking Scale ........ 94
B.10: National Institute Of Health Stroke Scale (NIHSS) ........ 95
B.11: Glasgow Coma Scale (GCS) ........ 102
B.12: Fiberoptic Endoscopic Evaluation of Swallowing Protocol for Tracheostomy Decannulation ........ 103

# 1. List of Abbreviations and Definitions

| Abbreviation | Definition |
|---|---|
| ADE | Adverse Device Effect. |
| AE | Adverse Event. |
| CA | Clinical Agreement also referred to as the Investigator Agreement. |
| CDA | Confidentiality Disclosure Agreement. |
| CIP | Clinical Investigation Plan (may also be referred to as a protocol). |
| CRF | Case Report Form (may be in paper or electronic format). |
| DHI | Dysphagia Handicap Index. |
| DSRS | Dysphagia Severity Rating Scale. A modified version of the Dysphagia Outcome and Severity Scale (O'Neil et al. 1999) designed to rate the severity of dysphagia in patients. |
| EAT-10 | The Eating Assessment Tool. |
| ECG | Electro-encephalogram. |
| EEG | Electrocardiogram. |
| EMEAC | Europe, Middle-East, Africa and Canada. |
| EPS1 | Phagenyx Base Station Model name. |
| EQ5D | Euro-QoL-5D. |
| FEES | Fiberoptic Endoscopic Evaluation of Swallowing. |
| FOIS | Functional Oral Intake Scale. |
| GCP | Good Clinical Practice. |
| GCS | Glasgow Coma Scale. |
| IB | Investigator Brochure. |
| ICU | Intensive Care Unit. |
| IFU | Instruction For Use. |

| | |
|---|---|
| LTFU | Lost-to-follow-up. |
| LOC | Level of Consciousness. |
| MDD | Medical Device Directive (93/42/EEC as amended by 2007/47/EC). |
| NMES | Neuromuscular Electrical Stimulation. A technique for electrical stimulation of the neuromuscular junction. |
| NGT | Nasogastric tube. |
| PAS | Penetration-Aspiration Scale (Rosenbek et al. 1996). A scale for measuring the degree of aspiration during a videofluoroscopy swallowing assessment. |
| PEG | Percutaneous Endoscopic Gastrostomy. |
| PES | Pharyngeal Electrical Stimulation. |
| Phagenyx | The investigational device name (base station and catheter). |
| PI | Principal Investigator. |
| QoL | Quality of Life. |
| RIG | Radiologically Inserted Gastrostomy. |
| Rx | Abbreviation for Therapy. |
| SADE | Serious Adverse Device Effect. |
| SAE | Serious Adverse Event. |
| SLT | Speech And Language Therapist. |
| SSS | Secretion Severity Scale. |
| STEPS | A Study of Swallowing Treatment using Electrical Pharyngeal Stimulations. |
| TBI | Traumatic brain injury. |
| UADE | Unexpected Adverse Device Effect. |
| VAS | Visual Analogue Scale. |
| VFS | Videofluoroscopy. |

## 2. Statement of Conformity

The undersigned hereby declares that the medical device specified for the Clinical Investigation **(AHE02: A prospective, single-arm, observational clinical follow-up study on the application of PHAryngeal electrical stimulation for treatment of neurogenic Dysphagia: a European Registry (PHADER))** is a CE marked device that conforms with the essential requirements of the Medical Device Directive 93/42/EEC (MDD).

Signed by responsible representative:

| | |
|---|---|
| **Name:** | Dr Conor Mulrooney |
| **Title:** | Chief Operations Officer |
| **Company:** | Phagenesis Limited<br>Unit 18<br>Enterprise House<br>Pencroft Way<br>Manchester Science Park<br>M15 6SE |

| | |
|---|---|
| **Signature:** | |
| **Date of Signature** | 10 APRIL 2015 |

# 3. Synopsis

| | |
|---|---|
| **Title:** | A prospective, single-arm, observational clinical follow-up study on the application of **PHA**ryngeal electrical stimulation for treatment of neurogenic **D**ysphagia: a **E**uropean **R**egistry (**PHADER**). |
| | |
| **Competent Authority:** | Not applicable |
| | |
| **Objectives:** | The primary objective of this observational, prospective, post-market follow-up clinical study is to demonstrate the real world clinical outcome of Pharyngeal Electrical Stimulation (PES) for the treatment of dysphagia resulting from different neurogenic disorders.<br><br>The secondary objectives identify the strengths and weaknesses of the treatment for neurogenic dysphagia (see secondary end-points); these characteristics are assessed via standard tools. |
| | |
| **Endpoints:** | The primary end-point is to assess the severity of dysphagia after PES-treatment at the time of pre-hospital discharge, by means of relevant standard assessment scales (DSRS[1], FOIS[2], PAS[3]) and to compare this with the patient's baseline condition.<br><br>The secondary end-points are related to the application, indication and general outcome status of the patient. They are descriptive in nature and should help to further understand standard practice:<br>- Demographics, symptoms and description of underlying dysphagia causes,<br>- Eating Assessment Tool (EAT-10) and Secretion Severity Score (SSS) and Dysphagia Handicap Index (DHI) to assess severity of swallowing,<br>- SLT[4]-management plan and execution,<br>- Measurement of the time and number of attempts to insert the stimulation catheter and ease of catheter positioning,<br>- Time point of PES treatment delivery in relation to dysphagia causal event and changes in DSRS,<br>- Treatment optimisation parameters (threshold, tolerance and intensity of the electrical stimulation), |

[1] DSRS = Dysphagia Severity Rating Scale
[2] FOIS = Functional Oral Intake Scale
[3] PAS= Penetration Aspiration Score
[4] SLT = Speech and Language Therapist

| | |
|---|---|
| | - Recording of subjective and personal appreciation of PES prior to and at the end of the study,<br>- Feeding status: FOIS-score, PEG[5]-status,<br>- Artificial ventilation status: type, purpose, duration of ventilation and cannulation (tracheotomy) and timing of decannulation, impact on feeding status,<br>- Quality of Life (QoL): EURO-Qol-5D,<br>- Disease severity and functional ability: Barthel Index, National Institutes of Health Stroke Severity (NIHSS) scale, modified Rankin Scale (mRS), Glasgow Coma Scale (GCS),<br>- Adverse Events (AEs) and device deficiencies: focus on chest infection rate secondary to aspiration and on device deficiencies as part of the post-market product surveillance program,<br>- Health economics: duration of ICU-stay/hospitalisation/ stay at different care giving units/discharge to home and of the total duration of ventilation/cannulation,<br>- Rehabilitation practice: patient flow within the hospital, to a rehabilitation centre and/or to home or another treatment unit/institute. |
| | |
| **Indication:** | Patients with neurogenic dysphagia:<br>a) As a result of stroke but not requiring mechanical ventilation or tracheotomy;<br>b) As a result of stroke and requiring mechanical ventilation or tracheotomy;<br>c) Associated with mechanical ventilation but not related to stroke or traumatic brain (TBI);<br>d) As a result of TBI or spinal cord injury with or without the need of mechanical ventilation or tracheotomy;<br>e) As a result from any other cause not associated with any element of groups a-e;<br>are specifically indicated to participate in this registry. |
| | |
| **Investigation Design:** | Prospective single-arm, observational clinical follow-up study. |
| | |
| **Number of Patients:** | A total number of 300 patients will be enrolled in the study with an attempt to enrol equal patient numbers into each of the 5 main patient indication groups (a-e). |
| | |
| **Target Population:** | The target population are patients with neurogenic dysphagia in the 5 main indications groups (a-e) that meet all the inclusion and exclusion criteria and are considered eligible to be entered into this clinical investigation. |
| | |

[5] PEG = Percutaneous Endoscopic Gastrostomy

| Inclusion/Exclusion Criteria | **Inclusion Criteria**<br>Patients are eligible for study participation if they:<br>- Are suspected to have oropharyngeal dysphagia with a DSRS score of 6 or higher; OR (when eating independency would be jeopardised by partial or total paralysation of upper extremities and when DSRS-assessment becomes invalidated) have a FOIS-score equal to or lower than 5; OR (when no oral food intake is possible and DSRS score is 12 and FOIS-score is 1) have a PAS-score of 4 or higher; AND<br>- Have a well identified dysphagia causing neurological event such as, but not limited to, stroke or brain injury, potentially but not necessarily leading to the need of long-term[6] mechanical ventilation or a tracheotomy; AND<br>- Are over 18 years old; AND<br>- Give themselves or have legal relatives/authorities representing themselves to give voluntary written informed consent;<br>And if they do not meet any of the exclusion criteria listed hereunder.<br><br>**Exclusion Criteria**<br>Patients are excluded from study participation if they;<br>- Have an undefined date of medical event causing the dysphagia; or<br>- Suffer from non-neurogenic dysphagia (e.g. cancer); or<br>- Participate in any other study potentially influencing the outcome of PES, both medicinal or medical device product related and for which the patient signed a consent form for his/her study participation; or<br>- Receive or have received within one month prior to the intended PES treatment any other type of standard cranial or percutaneous electrical stimulation therapy to treat dysphagia; or<br>- Have a cardiac pacemaker or a cardioverter defibrillator implanted unless the device can be switched off completely at the time of treatment delivery; or<br>- Have experienced an oesophageal perforation, or have an oesophageal stricture or pouch; or<br>- Have an unstable cardiopulmonary status; or<br>- Receive continuous oxygen treatment or have the equipment for such treatment permanently in place preventing the positioning of the Phagenyx Catheter (this does not exclude patients that can have the oxygen |
|---|---|

[6] 'Long-term' is defined as 20 consecutive days with a minimum of 6 hrs of ventilation daily.

| | |
|---|---|
| | treatment temporarily stopped and equipment removed during PES-treatment (See footnotes 25 and 26)); or<br>- Are pregnant or nursing women; or<br>- Require emergency treatment, preventing appropriate conduct of the patient informed consent process; or<br>- Have a life expectancy less than the duration of the patient's follow up period, i.e. less than three months. |
| | |
| **Length of Investigation:** | The enrolment period is anticipated to take 18 months. All patients will be followed up for 3 months. |
| | |
| **Device:** | The medical device (Phagenyx base station and intraluminal treatment catheter) is intended for professional use and is designed for the treatment of oropharyngeal dysphagia of neurogenic origin. The device functions by using electrical stimulation of the pharynx to effect sensorimotor reorganisation of control of the swallowing function within the brain. All patients will receive the standard treatment of 10 minutes PES for three consecutive days at an optimised intensity level set for each treatment (at 75 % of the tolerance limit and set above the threshold). PES for treatment of neurogenic dysphagia was granted a CE-mark in 2012. |
| | |
| **Comparator Clinical Investigations:** | In this study, there is no control group or other comparator involved. Each patient serves as his/her own control. |
| | |
| **Statistical Analysis:** | Quantitative clinical data will be represented as Mean $\pm$ Standard Deviation and/or median values. Categorical variables will be described as percentages. Primary end-points measured post-PES and prior hospital discharge will be compared within the respective group indications with the assessment prior-PES, i.e. at baseline. Adjustments for appropriate stratification variables (identified as secondary end-points) will be implemented as reasonable and justifiable. |
| | |

# 4. Background and Rationale

Dysphagia might appear as the result of different indications and one can differentiate 3 groups:

1. Oropharyngeal dysphagia;
2. Oesophageal dysphagia;
3. Functional dysphagia without organic cause.

Neurogenic dysphagia can be caused by multiple events:

- Stroke,
- Alzheimer’s disease,
- Parkinson’s Disease,
- Multiple sclerosis,
- Amyotrophic lateral sclerosis/Motor Neurone Disease,
- Huntington’s disease,
- Head injury,
- Guillain-Barré syndrome,
- Meningitis,
- Poliomyelitis,
- Critical illness neuropathy.

From this list, ‘stroke’ is known to cause the highest incidence of neurogenic dysphagia and was the subject of investigation in the STEPS study.

The diagnosis of dysphagia is most often based upon:

- decreased gag reflex,
- decreased ability to cough,
- malnutrition or dehydration,
- fever caused by infection of the lungs as a result of food entering the lungs (aspiration),
- depression.

but additional diagnostic tests may be needed to identify the neurogenic basis. Diagnosis of dysphagia is most often confirmed by means of VFS[7] or FEES (Kelly, Hydes, McLaughlin, & Wallace, 2007)[8], although other practices can lead to the same valid conclusion: oesophagoscopy, laryngoscopy, ultrasonography, CT-scan, MRI, chest X-ray.

In addition the neurological condition may be worsened by certain medication (anaesthetics, sedatives, neuroleptica etc.).

The severity of dysphagia can be altered by medication which is most often linked to the underlying disease, but medication also can cause dysphagia. Often alternative diets or special

[7] VFS = videofluoroscopic swallow study also known as the modified barium swallow
[8] FEES = Fiberoptic endoscopic evaluation of swallowing

exercises facilitate swallowing. In worst cases, enteral feeding is required or even surgical interventions can be applied to help prevent unintentional aspiration.

SLT[9] therapy is commenced upon diagnosis of dysphagia. In addition to the implementation of a special diet regimen, compensatory strategies and postural changes are applied separately or in tandem with a variety of stimulation strategies to treat dysphagia. Therapies are often applied at the discretion of the SLT and little or no scientific evidence is available to predict the outcome of the therapy nor the effect of the SLT therapy on the outcome of PES (Bath, Bath-Hextall, & Smithard, 1999) (Greeganage, Beavan, Ellender, & Bath, 2012).

In stroke-patients, PES at specific frequencies has been shown to enhance brain plasticity (Fraser, et al., 2002). This concept led to the development of the (Phagenesis sponsored) STEPS study (AHE01 - A multi-centre, double blind, randomised controlled Clinical Investigation to validate the EPS1 device as a treatment for stroke-induced dysphagia: A Study of Swallowing Treatment using Electrical Pharyngeal Stimulation). Electrical stimulation can be applied transcutaneously, intramuscularly or intrapharyngeally, however the therapeutic benefit as well as the underlying mechanism of action is not yet fully elucidated and the incorporation of electrical stimulation as part of the dysphagia therapy is left to the discretion of the physician.

The nature of neuro/muscular electrical stimulation to treat dysphagia is prone to research. Several clinical studies are currently on going (most often in acute or post-stroke patients) to demonstrate the benefit for dysphagia of direct or percutaneous PES in association or not with standard SLT-therapies (not restrictive list from ClinicalTrial.gov):

- NCT01971320: Evaluation of transcutaneous electrical stimulation in post-stroke dysphagia (TENSDEG)
- NCT01970384: Transcranial direct current stimulation for dysphagia therapy in acute stroke patients.
- NCT01956175: Electrical pharyngeal stimulation for dysphagia therapy in tracheostomised stroke patients.
- NCT01777672: Effect of afferent oropharyngeal pharmacological and electrical stimulation on swallow response and on activation of human cortex in stroke patients with oropharyngeal dysphagia.
- NCT01723358: Neuromuscular electrical stimulation (NMES) treatment technique therapy in the management of young infants with severe dysphagia.
- NCT02007759: Neuromuscular electrical stimulation (NMES) for dysphagia in neonates.
- NCT01697891: A pilot study of ALTENS in improving dysphagia induced by IMRT for head and neck cancers.
- NCNT01731847: Combined NMES, FEES and traditional swallowing rehabilitation in the treatment of stroke-related dysphagia.

Frequency and current amplitude of electrical stimulation, frequency of treatments and assessment methodologies vary strongly between studies and obscure interpretation of clinical outcome results. The conduct of these prospective, often randomised and blinded studies is justified to elucidate the benefit of a given therapy for a given patient. However, many questions

[9] SLT = Speech and Language Therapist

remain unanswered at this stage, while the therapy is easy and safe to apply and initial clinical findings sound a very promising therapy for dysphagia; to name a few:

- What is the best timing to start applying the PES after an event?
- Is repeated treatment effective? What interval between treatment is optimal?
- What is the effect of standard SLT-therapy in conjunction with PES?
- How to best evaluate the clinical outcome of PES in each patient sub-group?
- Can PES influence the timing of extubation in patients supported with mechanical ventilation or the decannulation in tracheotomised patients?

Commercial devices are however available on the market and are used outside of the controlled boundaries of a clinical study. They are applied to the indicated patient populations, however with less stringent “exclusion criteria”. It is important to know and understand the clinical outcome of the use of these commercially available devices in a ‘real world’ environment as they may differ slightly from the outcomes observed under the strict conditions of a (randomised and blinded) clinical study. This post-market clinical follow-up study is designed to document, in a prospective manner, the use and clinical outcome of the Phagenyx devices while supporting the product surveillance activities of Phagenesis Limited as recommended by the Medical Device Directive (93/42/EEC amended by directive 2007/47/EC).

A large variety of parameters exist to quantify the clinical outcome of the treatment of dysphagia, i.e. of the function of swallowing. Unfortunately, these quantification tools (instruments, questionnaire etc.) can also be applied with a certain degree of subjectivity or variability, and so are not always absolute measures. To reduce this variability, some guidance is given as to how to apply these tools best. The variability also illustrates the need to use multiple tools to assess the clinical outcome best.

# 5. Device Used in the Clinical Investigation

## 5.1. The Base Station, it’s Stimulation Catheters and Electrical Stimulation

The device used as part of this clinical investigation is the CE-marked Phagenyx Base Station (EPS1) and Phagenyx Catheter. These are commercially available and apply a standard electrical stimulation treatment at 5 Hz via the Phagenyx Catheter which is in essence a standard nasogastric feeding tube with built-in stimulation electrodes. The intensity of stimulation is optimised for each treatment by the Base Station software and operator input by setting the intensity at 75% of the tolerable limit above sensory threshold. The catheter houses an integrated circuit that allows the application of the therapy three times (during consecutive days). It is recommended to execute the calibration and 10 minutes stimulation **at the same time point** during the three consecutive days.

The CE-mark indicates the Phagenyx products are for the treatment of neurogenic dysphagia by means of the above-described PES-method.

This observational study applies to the products within the boundaries of its labelling.

The investigator brochure (IB) – amendment 4 dd 21 January 2014 – which is provided to each of the investigators of this study, contains detailed information pertaining to the manufacturing, instructions for use, packaging and labelling etc.

### 5.2. Device Accountability

The devices used in this clinical investigation are non-investigational devices; they are CE-labelled, are commercially available and will be used within the intentions of the label. As these devices are owned by and are part of the hospital inventory Phagenesis will not maintain overviews of local device stock as part of the clinical investigation management, yet each device used will be identified by a serial number and recorded on the appropriate data forms.

### 5.3. Risks and Benefits of the Product and Clinical Investigation

The investigator brochure (IB) – amendment 4 dd 21 January 2014 – which is provided to each of the investigators of this study, lists all considerations that were made to reduce and limit risks to the patient and users. This assessment, together with the current knowledge from the Phagenesis STEPS study (AHE01), has created the basis for the listing of anticipated adverse device effects (ADEs) which are provided in Appendix A.1.[10]

## 6. Objectives

### 6.1. Objectives and Endpoints of the Post-Market Release Follow-Up Study.

As per the Medical Device Directive (93/42/EEC, amended by Directive 2007/47/EC) the manufacturer can perform post-market clinical follow-up studies to comply with the directive's product surveillance requirements (post-market surveillance). For this purpose an observational study without any requirements to apply medical treatment beyond standard medical practice is judged appropriate. The prospective documentation of device related adverse events (ADEs) would suffice for such, yet given the novelty of the PES treatment, the medical community recommended that additional scientific background information be searched allowing analysis of the clinical data on a scientific basis and formulation of conclusions on correlations between different assessment tools. The current study approach justifies the extent of clinical data collection, yet there is a two-tier approach incorporated for the data collection.

### 6.2. Hypothesis

There is no specific hypothesis to be tested in this registry-type of clinical investigation: the purpose is to obtain more evidence on the use and clinical outcome of the Phagenyx

[10] Appendices indicated with "A" refer to study specific documents, forms etc., while appendices referring to "B" sections refer to standard, public domain documents, forms etc.

products when used to treat neurogenic dysphagia in daily practice (a 'real world' environment).

## 6.3. Primary Objective

The primary objective of this observational registry is to demonstrate the 'real world' clinical outcome of PES treatment for neurogenic dysphagia resulting from different causes post-PES, i.e. just prior hospital discharge. The clinical outcome is defined by means of standard functional scoring systems: DSRS, FOIS and PAS-scores (Appendix B.1, B.2 and B.4)

It is believed that these functional scores represent appropriate tools to assess the clinical outcome, yet other scoring systems might be as valuable. For the sake of simplicity these are referred to as secondary objectives/end-points.

## 6.4. Secondary Objectives

Secondary objectives of the registry are:

- To obtain additional evidence for the continued product safety and performance profile;
- To verify the product's safety and performance profile when exposed to a larger and more varied population of clinical users than studied thus far;
- To describe the demographics, symptoms and underlying causes of neurogenic dysphagia in treated patients;
- To assess the severity of dysphagia by means of standard scoring systems;
- To assess the severity of secretion by means of a standard scoring system;
- To describe the ease and appreciation by SLT and physicians delivering PES;
- To document the time point of PES therapy delivery in relation to the causal event;
- To describe the SLT management plan prior-PES and it's execution in conjunction with PES;
- To describe the SLT therapy between causal event, prior- and after PES-treatment;
- To measure threshold, tolerance and treatment current of PES on different days of delivery;
- To assess the effectiveness of PES at different time points during the day;
- To document the feeding status at baseline and post-PES at the time of pre-hospital discharge;
- To document the artificial ventilation status at baseline and post-PES at the time of pre-hospital discharge;
- To document the duration of cannulation (intubation/tracheotomy) and timing of decannulation with respect to the timing of PES-treatment;
- To document the patient's Quality of Life (QoL) using a variety of assessment methods at baseline and post-PES at hospital discharge and at three months post-PES;
- To document serious adverse events (SAEs) and device deficiencies during the observational study period;

- To document the health economic aspects of treatment by means of measurement of duration of hospital stay and duration of mechanical ventilation;
- To document the rehabilitation practices by means of defining the patient flow from one care given unit to another or to their own home over a time period of three months.

## 6.5. Primary Endpoint

The primary objective is assessed by means of two standard functional scoring systems and one instrumental scoring system:

- DSRS: Dysphagia Severity Rating Scale
- FOIS: Functional Oral Intake Scale
- PAS: Penetration Aspiration Scale[11]

These scores will be documented after the causal event and within one-week prior-PES (baseline), between 24 and 72 hrs post-PES and at pre-hospital discharge[12]. All values will be compared with the baseline assessment to establish the therapy benefit.

## 6.6. Secondary Endpoints

The secondary objectives are assessed by means of the following clinical data at one or more time point: baseline, at time intervals of 24-72 hrs, at pre-hospital discharge and at three months.

- Demographics described in terms of weight, age, sex, BMI, and assessment of distance between nostrils of the nose and the pharynx based on some external facial anatomical measures;
- Recording of the symptoms and underlying causes of dysphagia in treated patients;
- Assessment of the severity of swallowing using the Eating Assessment Tool (EAT-10) score - see Appendix B.4;
- Assessment of the severity of secretion by applying the Secretion Severity Scale (Murray, Langmore, Ginsberg, & Dostie, 1996) – see Appendix B.5;
- Measurement of the time and number of attempts to insert the stimulation catheter;
- Assessment of the easiness to position the catheter;
- Recording of subjective and personal appreciation of PES prior and at the end of the study;
- Recording of the different SLT therapies in addition to the management plan at baseline;

---

[11] For PAS-score determination either VFS or FEES methods can be applied. The choice is left to the discretion of the physician and should be in accordance with local practice and regulations.
[12] Pre-hospital discharge is defined as the time point where the physician decides that the patient could leave the ward/hospital when only considering their dysphagia; if this time point extends beyond 3weeks post- PES, then the follow-up assessments will be conducted at the 3 week-time point.

- Measurement of the time period between PES therapy delivery and the dysphagia causal event;
- The outcome of DSRS and other standard scores related to the time of the day PES is first delivered;
- Recording of the treatment optimisation parameters prior to every treatment delivery (sensory threshold, upper tolerance limit of the treatment current and the determination of the (75%) optimised treatment level);
- Assessment of feeding status by means of the FOIS-score and the PEG-status;
- Recording of the type, purpose and duration of artificial ventilation;
- Recording of the timing of extubation or decannulation (in tracheotomy patients);
- Assessment of the patient's QoL using the EURO-Qol-5D questionnaire – see Appendix B.7;
- Assessment of disease severity and functional ability using multiple questionnaires such as: Barthel Index, mRS, NIHSS scale and GCS – see Appendices B.6-11 respectively;
- Recording of any device related AEs and device deficiencies[13] continuously during the observational study period;
- Recording of any health economics by assessment of the duration of the Intensive Care Unit (ICU)-stay/hospitalisation/stay at different care giving units/discharge to home and of the total duration of mechanical ventilation/cannulation for a given purpose;
- Recording of the rehabilitation practice by the hospital discharge disposition at discharge from ICU, or from another care giving unit to home or another treatment unit/institute.

# 7. Patient Selection

## 7.1. Target Population

Patients with neurogenic dysphagia following stroke, traumatic brain or spinal cord injury, neurogenic dysphagia causing long-term ventilation, or patients suffering from other neurogenic dysphagia are indicated to participate in this registry. Patients can be classified into five mutually exclusive groups:

a. Neurogenic dysphagia as a result of following stroke not requiring mechanical ventilation or tracheotomy;
b. Neurogenic dysphagia as a result of stroke and requiring mechanical ventilation or tracheotomy;

[13] Definitions used for AEs and device deficiencies are those that are described in the ISO 14155 standard (version 2011): Clinical Investigation of Medical Devices for Human Subjects: Good Clinical Practice.

c. Neurogenic dysphagia associated with mechanical ventilation but not related to stroke or Traumatic Brain Injury (TBI);
d. Neurogenic dysphagia as a result of TBI or spinal cord injury with or without the need of mechanical ventilation or tracheotomy;
e. Neurogenic dysphagia as a result from any other cause not associated with any element of groups a-d.

Patients (or their legal representatives) must be willing to sign a standard Informed Consent Form explaining the conditions of study participation. Elected patients must be compliant with the inclusion and exclusion criteria before they can be considered enrolled in the study. It is emphasised that the patients are not exposed to any other treatment or therapy than what is standard of care and that this study only collects and analyses clinical data which otherwise are documented in the patient records.

## 7.2. Inclusion Criteria

Patients are eligible for study participation if they:

- Are suspected to have oropharyngeal dysphagia with a DSRS score of 6 or higher; OR (when eating independency would be jeopardised by partial or total paralysation of upper extremities and the DSRS-assessment becomes invalidated) have a FOIS-score equal to or lower than 5; OR (when no oral food intake can take place and DSRS score is 12 and FOIS score is 1) have a PAS score of 4 or higher; AND
- Have a well identified dysphagia causing neurological event such as, but not limited to, stroke or brain injury, potentially but not necessarily leading to the need of long-term[14] mechanical ventilation or a tracheotomy; AND
- Are over 18 years old; AND
- Give themselves or have legal relatives/authorities representing themselves to give voluntary written informed consent;

And if they do not meet any of the exclusion criteria listed hereunder.

## 7.3. Exclusion Criteria

Patients are excluded from study participation if they;

- Have an undefined date of medical event causing the dysphagia; or
- Suffer from non-neurogenic dysphagia (e.g. cancer); or
- Participate in any other study potentially influencing the outcome of PES, both medicinal or medical device product related and for which the patient signed an informed consent for his/her study participation; or
- Receive or have received within one month prior to the intended PES treatment any other type of standard cranial or percutaneous electrical stimulation therapy to treat dysphagia; or

[14] 'Long term' is defined as 20 consecutive days with a minimum of 6 hrs of ventilation daily.

- Have a cardiac pacemaker or a cardioverter defibrillator implanted unless the device can be switched off completely at the time of treatment delivery; or
- Have experienced an oesophageal perforation, or have an oesophageal stricture or pouch; or
- Have an unstable cardiopulmonary status; or
- Receive continuous oxygen treatment or have the equipment for such treatment permanently in place preventing the positioning of the Phagenyx Catheter (this does not exclude patients that can have the oxygen treatment temporarily stopped and equipment removed during PES-treatment (See footnotes 25 and 26)); or
- Are pregnant or nursing women; or
- Require emergency treatment, preventing appropriate conduct of the patient informed consent process; or
- Have a life expectancy less than the duration of the patient's follow up period, i.e. less than three months.

## 7.4. Sample size

A total of 300 patients with a first PES applied after a well-identified causal neurological event will be enrolled in the study over a time period of up to 36 months (estimated enrolment period). Each patient will be followed-up for a period of three months.

Patients are enrolled according to the five main distinct patient groups; it will be attempted to enrol an equal number of patients in each of these groups (n=60). This might cause closure of enrolment of patients in one or more groups before the end of the study enrolment period. If this attempt does not seem feasible for one or more groups this strategy will be abandoned and additional enrolment in other groups reopened or continued until a total of 300 patients are enrolled in the study.

Enrolment of patients in one specific subgroup but coming from one single study centre will be limited to a maximum of 50% of that patient group, i.e. to a maximum of 30 patients in that subgroup. If enrolment would be limited by this, and completion of the study would become jeopardised, this approach can be overruled.

## 7.5. Patient Enrolment and Withdrawal Criteria

A signed (by the patient or a legal representative) Informed Consent Form is required prior to performing any study screening procedure. Patients are considered as enrolled in the study once a first attempt to deliver PES-treatment is initiated (i.e. a Phagenyx Catheter is inserted). Patients may be replaced if PES-treatment is not deliverable (i.e. Phagenyx catheter insertion is not possible). Patients may withdraw from the investigation for any reason, at any time without their standard of care being affected. Patients that withdraw after receiving the PES-treatment are considered as being enrolled in the study; they will not be replaced upon voluntary withdrawal. All discontinuations will be documented along with the reason for withdrawal. Patient data collected up to the point of voluntary withdrawal will still be used.

### 7.6. Patient transfer from one treatment unit to another

A patient who is enrolled in the study in one study centre e.g. treatment unit and who is subsequently discharged to another centre/unit might get ‘lost-to-follow-up’. To limit the number of patients lost-to-follow-up, their follow-up is continued within the same centre/different unit under the responsibility of the original investigator; if the patient is discharged to another centre (or to home) efforts shall be undertaken to collect the required clinical data, possibly requiring local Ethics Committee approval to continue the study follow-up and transfer of investigator responsibilities to the new centre’s investigator. Although the patient is being treated and/or followed-up in more than one investigational centre, he/she is counted as one patient enrolled in the study irrespective of the additional therapies that are applied. All discontinuations will be documented along with the reason for withdrawal.

## 8. Study Design

This post-market clinical follow-up study is a prospective, multi-centre, European, observational[15] single-arm, clinical investigation to evaluate the clinical outcome of PES delivered at a given time after a neurological event causing dysphagia by means of the Phagenyx treatment (10 minutes of stimulation at an intensity level of 75% of the difference between the upper tolerable and lower threshold intensity level and set above this lower threshold at treatments during three consecutive days). All elements of this clinical study are observational, i.e. no specific medical interventions or clinical procedures are required to be applied – only “standard” or “routine” medical care will be applied, and available (‘real world’) clinical data are requested to be documented into the Registry for the purposes of post-market surveillance.

Patients meeting all of the study inclusion and who do not meet any of the exclusion criteria can be considered for study participation by physicians from selected investigational centres where the local Ethics Committee have approved the conduct of the study.

### 8.1. Investigational Sites

Only investigational sites that are acquainted with PES procedures are allowed to participate in this study. Each site will have dedicated medical personnel to apply the Phagenyx treatment and to use the appropriate treatment equipment.

[15] In an observational study, investigators assess health outcomes in groups of participants according to a protocol or research plan. Participants may receive interventions, which can include medical products, such as drugs or devices, or procedures as part of their routine medical care, but participants are not assigned to specific interventions by the investigator (as in a clinical trial). (Definition from ClinicalTrial.gov: https://clinicaltrials.gov/ct2/about-studies/learn).

Investigational sites participating in this registry will have demonstrated upfront the ability to use the Phagenyx treatment devices in an appropriate way after having received product training from the company.

Maximally, 30 different investigational sites can participate in the study and enrol patients. This excludes centres where a patient might be discharged to after having received PES in one of the selected investigational centres.

The centres are located in Europe, Middle-East, Africa and Canada (EMEAC-region). A centre performing only follow-up of a patient (e.g. a rehabilitation centre) is not considered one of the 30 investigational 'and enrolling' sites.

## 8.2. Identification and Selection

A list of investigational sites is available at the sponsor's site. The selection criteria are based on experience and a willingness to adequately support the study execution and its progress. Criteria include:

- Centres that have participated in the STEPS study;
- Centres that provide evidence of experience by having used the Phagenyx devices as part of their standard/routine medical care (as demonstrated by using the products in at least three patients);
- Centres that provide confirmation that a minimum of five patients will be enrolled in the registry over a maximum period of 36 months;
- Centres that are willing to comply with the requirements of the CIP such as, but not limited to, accurate and complete data collection on applied standard clinical processes using CE labelled product within it's labelling intention, obtaining Ethics Committee approval for the study, signing a CDA and CA[16], complying with the requirements for patient enrolment and follow-up, knowing and applying ISO 14155 requirements, complying with monitoring and auditing requirements etc.
- Centres having a 'clinical study team' available that is able to support the conduct of the study as confirmed upfront by the investigator.

## 8.3. Ethics Committee Approval

Each centre must obtain formal Ethics Committee approval prior to the start of the study and thus prior to enrolment of any patient. This approval must be forwarded to the sponsor who will provide at that time, access to the electronic data capture system. Submission documents from the sponsor are provided and additional support to apply for Ethics Committee approval can be provided upon request. The documents that are delivered to the investigator for this purpose are:

- Clinical Investigational Plan (CIP),
- Investigator Brochure (IB) – Amendment 4 dd. 21 January 2014,

[16] CDA and CA: Confidentiality Disclosure Agreement and Clinical Agreement.

- Participant (or personal/nominated consultee) Information Sheet and Informed Consent (declaration) Form provided in local language (see Appendix A.2. for English template versions),
- Clinical Agreement specifying the proposed compensation for study execution (see Appendix A.3. for an explanation on the standard template document – a template provided and accepted by the local investigation site might be added to the dossier for Ethic Committee submission, if available),
- Evidence of clinical investigation insurance (see Appendix A.4.),
- The structure of the database and data collection priority (see Appendix A.5.) and case report forms (CRF), i.e. indication of data collection blocks, to be used to document the clinical information (see Appendix A.5.),
- An overview of the data collection time points and a 'patient data flow chart' (see Appendix A.7)
- A "Patient Card", informing the reader about the study participation and data collection requirements, especially in case the patient is transferred to a rehabilitation centre to allow continued data collection (see Appendix A.8).

## 8.4. Site Activation

A clinical investigation centre can only start enrolling patients in the study after having obtained the required approvals and after the study staff have been appropriately trained on the different aspects of the CIP by the sponsor. Training records will be appropriately maintained both at the sponsor's and investigational site.

A formal approval for site activation is provided by the sponsor to the investigator after which access to the data collection forms (case report forms) will be granted.

## 8.5. Patient Discharge to Another Treatment Unit or to Home

Patient treatment and data collection do not pose a problem as long as a patient is treated within one care giving centre; transfer from one unit to another can be documented while the patient remains in the clinical study under supervision of the original investigator.

When a patient is discharged to another care giving centre at a time point earlier than three months following the first PES treatment, efforts must be undertaken to continue the data collection as part of this study. The sponsor should be informed about the discharge as soon as possible to allow implementation of appropriate actions to possibly include the new centre as an investigational 'follow-up' site if necessary. A Patient Card (see Appendix A.8.) is created informing the reader about the nature, status and requirements of the clinical investigation in order to reduce the number of patients who are considered lost-to-follow-up. Provisions should also be made so that the required clinical data are collected by appropriate personnel, after obtaining formal approval from the referral centre. A similar provision should be made in instances when a patient is discharged to their home prior to the three months (after the first PES treatment) study duration. This can be done via a telephone visit or by a personal visit of a hospital staff member upfront granted with an

approval from the sponsor; alternatively the patient can return to the hospital for a scheduled follow-up visit.

# 9. Investigation Procedures

This is an observational clinical study, i.e. no specific medical interventions or clinical procedures are required to be applied. Only “standard” or “routine” medical care will be applied, and available (‘real world’) clinical data are requested to be documented into the Registry study. The study is executed in compliance with the ISO 14155 (version 2011) and must be approved by the local Ethics Committee prior to commencement in the investigational centre. The voluntary participation of each patient, after being well informed, must be documented in writing.

The following table gives an overview of the clinical investigation.

| | **Screening** | **Baseline** | **Last day of PES Treatment** | **Follow Up-assessment 1** | **Follow Up - assessment 2** | **Follow Up - assessment 3** |
|---|---|---|---|---|---|---|
| *Timing (Period of data collection)* | *Any time* | *<14 days after screening & prior to first treatment* | *DAY 0*[17] | *>24 <72 hrs* | *Day 7 → day of discharge from unit and ≤ 21 days* | *Day 60→ day 120 days* |
| Informed Consent | X | | | | | |
| Inclusion/exclusion criteria assessment | X | | | | | |
| Demographic data | | X | | | | |
| Dysphagia causal event documentation (and medical history) | | X | | | | |
| Dysphagia severity assessment (DSRS) | **X** | (§)[18] | | X | X | X |
| FOIS-score | **X** | (§)[18] | | X | X | X |
| Dysphagia severity assessment (PAS) | **X** | (§)[18] | | X | X | (X)[19] |
| Feeding status documentation | | X | X | X | X | X |
| Ventilation and decannulation status documentation | | X | X | X | X | X |
| Other functional assessments | | X | | X | X | X |
| Quality of Life assessment (EQ5D) | | X | | X | X | X |
| SLT-management PLAN | | X | | | | |
| SLT-management ACTUAL | | | X | X | X | (X)[19] |
| PES treatment | | (§)[18] | X | | | |
| Care giving unit arrival/disposition | | | | X | X | X |
| Adverse Device Events (ADE) Severe Adverse Events (SAE) & Patient Deaths | | X | X | X | X | X |

[17] ‘DAY 0’: last day of first PES treatment delivered during the conduct of this investigation.
[18] (§): If time period between Screening and Baseline exceeds 14 days, dysphagia severity assessment should be repeated to re-establish dysphagia severity at baseline. Baseline and treatment day 1 can be the same day.
[19] (X): Indicates data collection only when patient is in the hospital.

## 9.1. Informed Consent

Every patient who is considered for enrolment in the study must be informed appropriately about the study intentions and their rights prior any further study related activity being executed. The procedures, study objectives and assessment tools/timing should be explained by the investigator (or designee) in layman's terms. The sponsor provides a (Patient) Participant (or personal/nominated consultee) Information Sheet providing all the relevant information (see Appendix A.2 for a template that can be used for local translation and adaptation[20]). All questions from the patient (or legally authorised representative) in relation to the study should be appropriately addressed. After having sufficient time to decide whether they wish to participate, the formal Informed Consent (declaration) Form (provided by the sponsor to the investigator in the local language and to be approved by the local Ethics Committee prior use by the investigator – see Appendix A.2) will be signed by the patient or by his/her legally authorised representative, as specified by local regulations and practice.

Only patients with an appropriately signed Informed Consent/Declaration Form can be considered for screening prior study participation. Upon signing the Informed Consent/Declaration Form, the patient will be issued a unique screening number.

The investigator will retain the original signed Informed Consent/Declaration Form in the study site file.

## 9.2. Patient Eligibility

The eligibility for study participation will be assessed after the patient or his/her legally authorised representative) has signed the Informed Consent/Declaration Form and prior to any other study related activity being executed. The patient screening should take place no later than one, and maximally two weeks after Informed Consent/Declaration is obtained. All inclusion and none of the exclusion criteria must be fulfilled in order to assign a patient as eligible for study inclusion, otherwise the patient will not advance any further into this clinical investigation and will exit the study.

## 9.3. Assessment Prior to Treatment

### *9.3.1.* ***Baseline assessment***

At baseline, a series of clinical data will be recorded which – all together – completes the baseline assessment. Baseline assessment should be completed within one week after the patient screening was successfully executed. The following baseline data will be collected (please also refer to the Investigation Procedures table, section 9):

**Demographic data**

- Age,

[20] Local Ethics Committee's may impose specific forms or specifications into the Information Sheets. If so, this will be documented and implemented accordingly.

- sex,
- weight,
- distance from the nasal septum to the ear lobe and from the ear lobe to the laryngeal prominence (Adam's apple).

### Dysphagia causal event documentation

The underlying cause of the neurogenic dysphagia and time of occurrence of the causal event should be documented. Experience with Phagenyx devices in specific patient groups will be studied. These (mutually excluding) groups are identified for patients suffering from neurogenic dysphagia associated with one or more of following symptoms and underlying causes:

- Stroke without need for mechanical ventilation support or tracheotomy;
- Stroke with need for mechanical ventilation support or tracheotomy;
- Any (non-stroke and non-Traumatic Brain Injury (TBI) related) cause with a need of mechanical ventilation or tracheotomy;
- TBI or spinal cord injury with or without the need for mechanical ventilation support or tracheotomy;
- Other causes leading to the diagnosis of neurogenic dysphagia and not associated with any element of groups A-E, i.e. similar to group C but without the need for mechanical ventilation or tracheotomy:
    - Alzheimer's disease,
    - Parkinson's Disease,
    - Multiple sclerosis,
    - Amyotrophic lateral sclerosis/Motor Neuron Disease,
    - Huntington's disease,
    - Guillain-Barré syndrome,
    - Meningitis,
    - Poliomyelitis
    - Critical illness neuropathy.

For each of these causes, relevant additional information on diagnostic methods, signs and symptoms, severity of disease etc. might be documented. For this e.g. the 'modified Rankin Scale' (Appendix B.9) and 'National Institutes of Health Stroke Scale – NIHSS' (Appendix B.10) are used in stroke patients, while the 'Glasgow Coma Scale' (Appendix B.11) is used in the TBI patients. Other assessment tools belonging to the standard local practice can also be applied. Any relevant current medication used to treat dysphagia will also be recorded, such as but not limited to dysphagia related medications etc.

### Dysphagia assessment

The level of severity of dysphagia will be assessed by means of:

- Dysphagia Severity Rating Scale (DSRS) as the primary end-point;

- Penetration Aspiration Scale (PAS) score using either VFS or FEES as the secondary end-point.

The Eating Assessment Tool – EAT-10 (Belafsky, et al., 2008) should also be used to further assess the severity of the swallowing problems as a secondary end-point. Also, the Secretion Severity Scale (SSS) rating (Murray, Langmore, Ginsberg, & Dostie, 1996) is assessed due to its linkage to dysphagia.

The applicable questionnaires for DSRS, EAT-10, PAS and SSS are listed respectively in Appendix B.1, B.3, B.4 and B.5.

#### Feeding status assessment

Feeding status – as a primary end-point – should be assessed by means of the Functional Oral Intake Scale – FOIS (Crary, Carnaby-Mann, & Gorher, 2005). The applicable questionnaire is listed in Appendix B.2. In addition, the use of a nasogastric feeding tube, PEG, radiologically inserted gastrostomy (RIG) or potentially the execution of other surgical interventions (e.g. to avoid dehydration) should be documented. Consistency of fluids, types of diets, amounts of food intake, duration of feeding sessions and need for supervision during feeding should also be documented.

#### Ventilation status assessment

Type, purpose and duration of artificial ventilation and/or tracheotomy should be documented. Timing of and reason for removal of ventilation tube should also be recorded.

#### Quality of Life (QoL) and functional assessments

QoL and functional assessment should be documented by means of three types of questionnaires:

- Barthel Index [21] (Mahoney & Barthel, 1965) – Appendix B.6
- Euro-QoL-5D (5 level answers) – Appendix B.7
- Dysphagia Handicap Index – DHI (Silbergleit, Schultz, Jacobson, Beardsley, & Johnson, 2012) – Appendix B.8

These questionnaires will be provided to the investigational sites in local language so that the patient can read and complete them, if possible, but it is anticipated that for some patients the completion of some questionnaires might not be possible. Should this be the case, the clinical site team will be asked to document in the appropriate data forms the reason for non-compliance of these assessments and/or if persons other than the patient him/herself completed the questionnaire.

[21] http://www.strokecenter.org/wp-content/uploads/2011/08/barthel.pdf - Mahoney FI, Barthel D. "Functional evaluation: the Barthel Index." Maryland State Med Journal 1965;14:56-61. Used with permission.

Many of the questionnaires used to assess dysphagia are not fully validated for the proper translation into local languages. Where possible the validated questionnaires will be used. An example is the Dysphagia Handicap Index (Appendix B.8); currently a translation validation study is on-going under the leadership of Dr J.E. Bohlender (see ClinicalTrial.gov ref: NCT01958268). The latest version of this translated questionnaire will be used in the study. EAT-10 is similarly only validated in English.

## 9.4. Treatment Procedure

### *9.4.1.* ***PES Treatment***

When a patient is successfully screened, meets all the inclusion and none of the exclusion criteria, and when the patient has received his/her first PES treatment (or when at least a first attempt is made to insert the Phagenyx catheter), then the 'screening number' will become a unique 'study enrolment number', and the patient is considered 'enrolled' in the study.

The standard PES treatment as described in the 'Instructions For Use' provided with the Phagenyx products is as follows:

- A pharyngeal nasogastric catheter (Phagenyx Catheter) is inserted at the bedside of the patient by a nominated person;
- Optimisation of the treatment parameters is conducted; sensory threshold and maximum tolerable limits are assessed by using the Phagenyx Base Station by which the difference is calculated between the tolerable stimulation intensity and the lower threshold and where stimulation levels are set at 75% of this difference above the lower threshold;
- The standard treatment regime is delivered; this is fixed to three consecutive days of 10 minutes of PES at 5 Hz at the determined stimulation level.

Typically, upon diagnosis of dysphagia, the SLT will help determine the best therapy for a given patient to recover the swallowing functionality. This might take place early or later after the dysphagia causing event. As part of this investigation, it is important to document the planned and actual SLT treatments/therapies (see below) in addition to the planned and actual PES-treatment.

If indicated, PES treatment should be planned upon diagnosis of dysphagia and become an integral part of the SLT management plan. Timing of PES treatment vis-à-vis the dysphagia causing event will be an important parameter to document alongside the treatment outcome as assessed by means of DSRS, PAS, FOIS and EAT-10. The optimal timing to start PES after the event, as well as the timing during the day to apply the PES treatment is left to the discretion of the investigator (Treatment Day 1 and Baseline can be the same day) but should be as constant as possible for a given patient. For reasons of consistency it is important to document the first application meticulously. Ideally no other therapy is delivered during the

observational period to elucidate optimally the effects of PES, however the decision to deliver in tandem other medication or other therapies is left to the discretion of the investigator. It is the objective of this study to document 'real world' medical therapy, thus concomitant application of multiple routine medical care activities is allowed and should be documented.

A special situation presents for patients who are orally intubated or tracheotomised and might require mechanical ventilation (also see footnotes 25 and 26). The use of PES in such patients is addressed in this study to understand if PES has any beneficial effect above and beyond the use of standard SLT-therapies on the advanced timing of extubation or decannulation. Reference is made to the practice of Professor R. Dziewas on this assessment (Warnecke, et al., 2013) and for which the Fiberoptic endoscopic evaluation of swallowing (FEES) protocol for tracheotomy decannulation is provided in Appendix B.12. This decannulation protocol should be followed on a voluntary basis but will allow assessment of this goal objectively.

The Phagenyx Catheter is intended to be for single patient use. It is possible that during use, the catheter is accidentally or intentionally removed. Repositioning, but eventually use of a new catheter, might be appropriate. The number of catheters used, the timing of placement and duration of use should be recorded as part of this registry. In addition, the technical settings of stimulation levels and effective application of the PES treatment, the easiness to introduce and position the catheter either as a first placement or as a replacement of an already inserted (feeding) catheter, and the number of attempts to complete insertion will be assessed and documented. In instances where certain study data are not routinely documented in the medical notes (e.g. individual treatment thresholds obtained during treatment optimisation, DHI, EQ5D etc.), these study data may be documented directly on to the provided study CRFs/questionnaires and will form part of the study's source documentation and thus should be handled accordingly.

#### *9.4.2.* ***SLT (Speech & Language Therapist) Treatment***

The SLT management plan is similar to the one applied in the STEPS study. As such results can be more easily compared.

SLT management plans are typically conducted by SLTs and are based upon a bedside swallow assessment, an objective (instrumental) swallow assessment such as a clinical VFS or FEES, or another local procedure that must be specified.

For reasons of consistency management, the type of clinical assessment used to inform the SLT management plan should remain consistent at all time points for each patient throughout the study, i.e. the swallow assessment type may vary between two different patients in the same hospital, but should not vary at different time points for the same patient during the study. Similarly, the procedure applied to

assess the PAS, e.g. number of swallows considered and amount of fluid for each swallow test, should be documented and remain consistent for a single patient. Similarly, more than one SLT may take part in this investigation but each SLT must be assigned consistently to a single patient throughout.

The SLT management plan at baseline (prospective planning), and the actuals, should be documented at the time of PES and at distinct time points throughout the three-month follow-up period. The SLT management plan will be recorded in the provided case report forms.

The applicable questions are listed in Appendix A.9.

## 9.5. Follow-up Evaluations

The follow-up period will start when the last PES treatment is delivered (please also refer to the Investigation Procedures table, section 9). This might be after the first day, when PES treatment is discontinued, or might be after a series of several days when repeated planned daily PES treatments are delivered. When additional PES treatments are delivered after a first treatment was considered completed, the original follow-up time schedule remains applicable. Yet, data from the additional PES treatments must be recorded as well. The last day of the first planned and delivered PES treatment constitutes "DAY O" and starts the clock for the follow-up assessments.

### Follow-Up Assessment 1 (Period > 24 hrs and ≤ 72 hrs)

A first follow-up assessment will be made in the time period between "24 hrs" and "72 hrs". The appropriate clinical data of DSRS, feeding and ventilation status, QoL, SLT management plan etc. will be assessed and documented. If, for whatever reason, additional PES treatments are applied or are scheduled for application, this shall be documented. Similarly, if an SAE, an ADE or patient death occurs, this shall be appropriately documented on the respective data forms and reported to Phagenesis. Also, if there are changes in the SLT-therapy (e.g. changes in diets for the better or worse) this shall be documented on the respective data form (Unscheduled Follow-Up Form).

If the patient is discharged in the specified time window (< 72 hrs) to another care giving institution, or to their home, then this assessment may be skipped in favour of completion of "Follow-up assessment 2".

### Follow-Up Assessment 2 (Day 7-Discharge or ≤ 21 days, whichever comes first)

A second follow-up assessment will be made in the time period between "DAY 7" and "DAY X", where "X" is determined by the day that the patient is discharged to another treatment unit because the patient no longer not needs to stay in the current unit for reasons of

dysphagia[22], within the same hospital, or to another care giving institution, or to their home, but at the latest at 21 days post Day 0. If the disposition takes place before “DAY 7” then this will also be documented on the ‘Follow-up assessment form 2”.

The appropriate clinical data of DSRS, FOIS, PAS, feeding and ventilation status, QoL, SLT management plan etc. will be assessed and documented (please also refer to the Investigation Procedures table, section 9).

Discharge disposition information will be collected to map out the geographically different practices and to help assure a minimum number of patients becoming lost-to-follow-up.

When a patient arrives at a treatment unit within the same hospital in the specified time window “Follow-up assessment 2” is repeated within the foreseen time window, however this cannot be prior to “DAY 7”, irrespective of the time of arrival of the patient on the treatment unit. If, for whatever reason, additional PES treatments are applied, this shall be documented. Similarly, if an ADE, SAE or patient death occurs, this shall be appropriately documented on the respective data forms.

When a patient arrives at a treatment institution (or at their home) different from the initial investigational centre, all attempts will be made to further follow-up the patient until the three-month follow-up session. For this, contact with the sponsor will be made to allow the new institution to identify a ‘follow-up investigator’, to obtain local Ethics Committee approval, if necessary, and to help assure continued data collection. If a patient is discharged to their home, similar attempts will be made to obtain the three-month follow-up data.

### Follow-Up Assessment 3 (Month 2-4 after Day 0)

A final follow-up assessment is scheduled within 2-4 months after “DAY 0”, either at the original investigational site, or at the treatment unit where the patient was discharged to, or from a third treatment unit, or from their home. Eventually, the assessment is performed via a telephone contact to retrieve as much clinical data as possible, yet the PAS-score requires the patient to be assessed in a hospital environment and therefore this assessment is only scheduled for patients residing in such an environment. If the follow-up session is to be done by telephone, and the patient is unable to communicate verbally, it will be permissible, with approval from the sponsor, for a member of the investigation site team to attend the patient’s place of residence to perform these assessments. At such an occasion as much clinical data collection as reasonably possible will be done.

The appropriate clinical data of DSRS, FOIS, PAS, feeding and ventilation status, QoL, SLT management plan etc. are assessed and documented (please also refer to the Investigation Procedures table, section 9).

[22] This time point might differ from the physical discharge of a patient from one care-giving unit to another. The investigator should identify the time point of ‘discharge’ exclusively on the status of the dysphagia, not on any other medical condition of the patient.

If, for whatever reason, additional PES treatments were applied in the period from the hospital discharge documented with the last "Follow-up assessment 2" until the current three-month follow-up session, this shall be documented. Similarly, if an ADE, SAE or patient death occurred, this shall be retrospectively and appropriately documented on the respective data forms, if not already done.

**Unscheduled Follow-Up Assessment (any time between Day 0 and hospital discharge)**
If there are considerable changes observed in the patient status requiring adjustment of the feeding regime and/or the ventilation and/or cannulation, then this is documented using an 'Unscheduled follow-up form'. This documentation serves primarily the purpose to reconstruct at the end of the study the changes that were applied in patient treatment, especially during the initial follow-up period after PES treatment and prior hospital discharge.

# 10. Adverse Events (AEs)

This clinical investigation is executed in accordance with the ISO 14155 (2011). The definitions of AEs listed in this standard apply to the study (see Appendix A.1). Post-market surveillance is required for CE-labelled products; this study lends itself to prospectively document any device related AE or any observed device deficiency and will be reported to regulatory authorities as appropriate.

Reporting requirements as part of the study are:

- All serious adverse device effects (SADEs) and non-device related AEs observed in a patient enrolled in the study;
- All unexpected adverse device effects (UADEs) observed in a patient enrolled in the study;
- All device deficiencies observed during the clinical study period;
- Any patient death, whatever the cause might be.

For clarity, general non-serious, non-device related AEs are not documented in the study. From the reportable AEs, sufficient information will be obtained so as to permit 1) an adequate determination of the outcome of the event (i.e. whether the effect should be classified as an SAE) and; 2) an assessment of the causal relationship between the AE and the Phagenyx devices. The following information will be collected for those AEs that require reporting:

- Title of Event
- Start date of event
- Intensity of event
- Frequency
- Outcome
- Relationship to Device/Procedure
- Seriousness Criteria

- Action Taken

Instructions will be given to the local study team with respect to the contact person at Phagenesis in case of observation of SAEs, SADEs and/or unanticipated AEs (see also Appendix A.1).

AEs occurring in any patient, who signed the informed consent form, will need to be documented during a subsequent period of three months as part of this study, whether or not the patient was ultimately enrolled in the study. If the patient was enrolled in the study, AE's will be documented during a three-month follow-up period starting at DAY 0 of the PES-treatment.

# 11.Data Management

## *11.1. Data collection system*

Data collection for this study will be completed via paper CRFs. These will constitute part of the original source documents that need to be maintained. Access to the blank CRFs will only be granted to the investigation site by the sponsor after: (i) appropriate training of involved staff members is completed, (ii) the site have obtained and provided the sponsor with proof of the local Ethics Committee approval and (iii) the clinical agreement is fully executed.

Data collected at investigational sites on paper CRFs will be anonymised at sites at the time of collection (e.g. site number/patient number; 01/001) and will not contain any patient identifiable information. Completed CRFs will be scanned and emailed securely to the study email account (AHE02@Phagenesis.com) between the site(s) and the sponsor with secure encryption in place. Access to this account is limited to only authorised personnel. Data from the CRFs will subsequently be transferred into an electronic data collection system by Phagenesis personnel or a suitably qualified designee. All electronic data will be stored securely on the Phagenesis server. Paper CRFs re-printed for data correction or data entry purposes will be filed and stored in a locked cabinet in the Phagenesis office. Only authorised personnel from Phagenesis or a suitably qualified designee will be granted access to the study data.

## *11.2. Data Collection: Two Tiers*

For every identified section of data collection there are a series of mandatory questions to be completed and a series of additional questions that can be completed on a voluntary basis. For this purpose, the investigator will sign-up upfront for either the basic (mandatory) or for both the mandatory and the advanced (optional) level of data collection. The basic tier (1) of data collection refers to the essential data that allow assessment of the primary objectives by collection of parameters listed as 'end-points'-parameters. The advanced (optional) tier (2) of data collection refers to additional relevant details related to a given parameter listed as

'end-point'-parameter, merely related to secondary objectives. Investigational centres will continue to collect their chosen level of data collection (tier 1 vs. tier 2), unless the change of preference of choice is requested and approved by Phagenesis.

Data collection on patient questionnaires (e.g. QoL) will be done via paper forms. These constitute the original source documents that need to be maintained; however the data collected will be transferred into an electronic data collection system by Phagenesis personnel or a suitably qualified designee.

The investigator is responsible to ensure that data collected is timely, is accurate and is as complete as possible.

### 11.3. *Monitoring, Auditing and Inspection*

A clinical monitoring plan, based on risk assessments and focussed on the adverse device related events, on the primary and – as much as possible – on the secondary objectives, is in place at the sponsor's site. This will be executed during the conduct of the study. At regular time intervals, monitoring will be executed either via electronic review of data completion, via electronic automated or manual queries, or at the site via source document verification during personal site visits.

Site visits will be scheduled at the time of site initiation, in the time period between the first and fifth patient enrolment and at study closure. Additional site visits will be planned based upon a risk assessment approach. Monitoring will be conducted by qualified personnel as assigned by the sponsor.

The sponsor plans to also execute a number of audits in selected centres to further help assure complete and accurate data collection, but also to help assure adherence to regulatory requirements.

Inspections can also be executed unexpectedly by regulatory authorities. In such an event, the investigator will explicitly allow these authorities to have access to the (patient) source data to allow review of the clinical study conduct per the current CIP. The patient Informed Consent Form will also refer to this access modality and the originals will be kept at hand during the total duration of the clinical study.

### 11.4. *Data Review, Cleaning and Queries*

Data will be reviewed by either sponsor's personnel or by assigned people from qualified clinical research organisations. Based on the review of the mandatory clinical data, queries will be generated to correct or further request any missing information. This will help to complete (clean) the database prior to any analysis being performed.

### 11.5. *Data Retention*

Clinical data collected in this investigation are not for the purpose to obtain regulatory approval to bring the product to market; they are from a (post-market)

product surveillance and scientific nature. In that perspective clinical data should be retained at the site for a minimum of five (5) years after completion of the clinical study; prior this date, no study related clinical data may be destroyed without prior written approval from the sponsor. A formal indication of the study closure date will be provided by the sponsor at the end of the investigation.

In case the investigator moves/retires, or otherwise leaves their post, they will provide Phagenesis with the name and address of the person assuming responsibility for records relating to this clinical investigation.

# 12. Suspension, Termination and Close-out of Clinical Investigation

Phagenesis may suspend or prematurely terminate either the clinical investigation in an individual investigation site or the entire clinical investigation for which the reasons will be documented. Reasons for suspension or premature termination at an investigation site may include incidences where monitoring or auditing identifies serious or repeated deviations from the protocol on the part of an investigator, but also unexpected low enrolment rates jeopardising the proper conduct of the study within the foreseen time windows. Phagenesis will ensure that the Ethics Committee (and if applicable, regulatory agency) is notified of any suspension or early termination of the clinical investigation, it will also notify all other principal investigators in the event that the suspension or termination was due to safety issues.

A principal investigator, Ethics Committee, or regulatory authority may suspend or prematurely terminate participation in the clinical investigation at the investigation sites for which they are responsible.

If suspicion of an unacceptable risk to patients arises during the clinical investigation, or when so instructed by the Ethics Committee or regulatory authorities, Phagenesis will suspend the clinical investigation while the associated risk is assessed. The sponsor will terminate the clinical investigation if an unacceptable risk is confirmed. Should the risk not be confirmed, Phagenesis will, in accordance with regulations, supply relevant persons with justification and data supporting the decision to resume the clinical investigation.

Routine close-out procedures will be conducted ensuring that the Investigator's records are complete, all documents needed for the sponsor files are retrieved, and previously identified issues have been resolved.

Enrolment of patients in a given patient group might be suspended when the target number of 60 is reached and until it is clear that enrolment in other groups might create an issue to reach the target number in a timely manner; at that time point enrolment in the first patient group might be re-opened.

Enrolment of patients (with a given indication) in a single centre might be suspended if the centre contributes 50% of the target patients in that specific patient group.

# 13.Statistics and Data Analysis

## *13.1. Hypothesis Testing*

There is no hypothesis postulated, hence there is no hypothesis testing planned.

## *13.2. Sample Size Determination*

Mutually excluding subgroups of patients suffering from neurogenic dysphagia have been identified upfront:

a. Neurogenic dysphagia as a result of stroke but not requiring mechanical ventilation or tracheotomy;
b. Neurogenic dysphagia as a result of stroke and requiring mechanical ventilation or tracheotomy;
c. Neurogenic dysphagia associated with mechanical ventilation but not related to stroke or TBI;
d. Neurogenic dysphagia as a result of TBI or spinal cord injury with or without the need of mechanical ventilation or tracheotomy;
e. Neurogenic dysphagia as a result from any other cause not associated with any element of groups a-e.

This study attempts to populate each subgroup with 60 patients, which is a relevant sample to determine absence of occurrence of an event (e.g. device deficiency) in less than 5 % of the population with a confidence level of 80%. It is anticipated that in the fifth group (e. neurogenic dysphagia as a result from any other cause but not associated with any element of groups a-e) only about 50% of the intended number of patients can be enrolled within the foreseen time period because of unavailability of patients in the study centres. Redistribution of the number of patients in short in this group to other subgroups will be done during the study when this issue becomes apparent. In order to count for lost-to-follow-up patients, an additional 10% of patients will be enrolled.

As such it is intended to enrol ((4 x 60) + (1 x 30)) + 10% = 297 patients. The target number is thus set at 300 patients for the total study population.

## *13.3. Data Analysis and Presentation*

Statistical analysis is restricted to descriptions of the different parameters (mean, median, standard deviation). In addition, for some parameters addressing the primary and secondary objectives, additional analyses will be executed.

Categorical data will be represented as percentages.

## *13.4. Analysis of Primary and Secondary Endpoints*

### Primary end-point analysis

The DSRS score is measured in every patient where the movement of arms and hands is not restricted at baseline and post-PES treatment prior hospital discharge. The change of this score will be determined in the total patient population and in the defined subgroups as 'intention-to-treat' and 'as treated' according to the PES-treatment applied. The statistical significance of the difference calculated from the paired observations (baseline ←→ post-PES) will be assessed via paired t-test and analysis of covariance, assessing different independent variables.

Changes of the FOIS-score between the identified time points will be assessed for the total patient population and for the distinct subgroups per the intention or the actual delivery of a PES-treatment.

In case the patient is tube-fed, e.g. a PEG implanted, and therefore the DSRS score is 12 and the FOIS is 1, then the changes in PAS score will be assessed from baseline to post-PES.

### Secondary end-points analyses

The extended data collection option in the PHADER (especially tier 2 data collection) lends itself uniquely to provide the medical community with information to better understand and assess the effect of PES and SLT-therapies in the treatment of dysphagia.

- Quantitative assessments of different questionnaires (DSRS, PAS, Euro-Qual-5D[23], EAT-10, DHI, Barthel Index, SSS, FOIS) will be described at the different time points of data collection (range, mean, distribution) and the variation over time assessed (trend analysis) for the whole population and for the different identified subgroups. [Note: for analysis of the Barthel Index, an additional score of -5 might be used for patients who died during the study].
- Additional analysis of parameters, relevant for each of the different subgroups of the population on the collected data from identified parameters will be descriptive in nature e.g. such as described previously (Almirall, et al., 2013) where stratification according certain comorbidities identified the importance of some for the occurrence of pneumonia in dysphagic patients.

## 14.Reports and Publications

***Scientific Committee***

A Scientific Committee is established, chaired by Professor S. Hamdy. This committee will address the intended data collection, in the five different patient groups specified in this study. Each

[23] Analysis of Euro-Qual-5D data will take into account the original scores as well as the adjusted scores per the applicable Health Utility Status.

member of the Scientific Committee will assume responsibility for the data analysis, interpretation and reporting of data collected in a specific subgroup of the study population. The patient groups and the representing Scientific Committee member are:

| | Patient Group | Scientific Committee Member |
|---|---|---|
| a | Neurogenic dysphagia as a result of stroke, but not requiring mechanical ventilation or tracheotomy. | Professor P. Bath, Nottingham, United Kingdom |
| b | Neurogenic dysphagia as a result of stroke and requiring mechanical ventilation or tracheotomy. | Professor R. Dziewas, Münster, Germany |
| c | Neurogenic dysphagia associated with mechanical ventilation but not related to stroke or to TBI. | Professor R. Likar, Klagenfurt, Austria |
| d | Neurogenic dysphagia as a result of TBI or spinal cord injury with or without a need of mechanical ventilation or tracheotomy. | Professor L. Saltuari, Innsbruck, Austria |
| e | Neurogenic dysphagia as a result from any other cause not associated with any element of groups 1-4. | Professor S. Hamdy, Manchester, United Kingdom |

## 14.1. Interim Report

No formal interim reports are foreseen other than those describing the progress of the study (enrolment, data collection, monitoring etc.), unless identified ad-hoc by the Scientific Committee.

## 14.2. Final Report

A final report will be created per the ISO 14155 guidelines. This final report will be reviewed by the Scientific Committee-members and approved by all investigators.

## 14.3. Publications and Publication Policy

A publication policy will be created by the members of the Scientific Committee, defining the content and the timing of specific publications addressing the symptoms, diagnosis, therapy and treatment outcome in the total patient population and in the specific subgroup of patients studied in this registry. Authorship rules will also be defined in that policy and will consider the numerical and scientific contribution to the study (e.g. enrolment rates, applying first and/or second tier of data collection, compliance to data collection requirements etc.). No publications other than those specified and approved by the members of the Scientific Committee are allowed to be published or presented in any kind during the conduct of the clinical study until the final report is approved and the investigation formally closed. Each investigator will be informed about the policy prior first enrolment of a patient in the study at the investigator's site and agrees formally with this approach by signing the Investigator Agreement.

# 15.Ethical Considerations

## 15.1. Declaration of Helsinki

The study is executed in line with the relevant articles of the Declaration of Helsinki as adopted by the 18th World Medical Assembly in 1964 and as revised repeatedly and lately in Seoul (2008).

## 15.2. Ethics Committee Approval

At a minimum the local Ethics Committee must formally approve the conduct of the study in the investigator's centre, unless local regulations dictate a different procedure. For this, the appropriate documents must be submitted and the investigator must provide the sponsor with a signed and dated letter granting approval from the local Ethic Committee.

## 15.3. Informed Consent and Patient Information

Each patient and/or their legal representative will be appropriately informed about the study purpose and their voluntary participation will be documented in writing by signing the provided Informed Consent/Declaration Form (see Appendix A.2). This document also states that all clinical data collected from a patient will be remain confidential, irrespective of the fact that the sponsor, delegates of the sponsor, and/or regulatory authority personnel can have direct access to the source data.

## 15.4. Patient compensation for study participation

No compensation is provided to the patients for study participation. Travel costs, if any, are reimbursed via the investigator's study centre upon provision of the receipts.

## 15.5. Patient Insurance – Indemnity / Patient Pay-Outs

The sponsor recognises its liability in law to compensate for any injury sustained by a patient participating in this clinical investigation when the injury directly evolves from the malfunctioning of the medical device (device deficiency) when correctly applied and used per the Instructions For Use.

The insurance will comply with local and national guidelines.

# 16.Good Clinical Practice (GCP) Compliance

## 16.1. Professional Conduct of the Clinical Study

Only well identified study centres can participate in the study. The centre selection criteria are specified to assure that appropriate medical, technical and clinical investigator expertise is present prior approval of study participation.

Prior the start of the study, the investigator will sign the CIP demonstrating the willingness to conduct the study according the specifications of the CIP.

### 16.2. Training Provided by Sponsor

Specific technical training to use the Phagenyx devices is not part of this study, given that the device is CE-marked and that availability of technical expertise is a requirement to participate in the study. Training is however provided by the sponsor to the investigator on the GCP aspects and as outlined in the ISO 14155.

### 16.3. ISO 14155 (version 2011)

The ISO 14155 describes the good clinical practices for conduct of clinical investigations in humans with medical devices. The study is set-up and is implemented according to these guidelines. Each centre will be trained on or should demonstrate acquaintance on the relevant elements of this standard prior first enrolment of a patient.

## 17.References

Almirall, J., Rofes, L., Serra-Prat, M., Icart, R., Palomera, E., Arreola, V., et al. (2013). Oropharyngeal dysphagia is a risk factor for community-acquired pneumonia in the elderly. *Eur Respr J*, 41: 923-928.

Belafsky, J., Mouadeb, D., Rees, C., Pryor, J., Postma, G., Allen, J., et al. (2008). Validity and Reliability of the Eating Assessment Tool (EAT-10). *Annals of Otology, Rhinology & Laryngology*, 117 (12): 919-924.

Chris Fraser, M. P. (2002). Driving Plasticity in Human Adult Motor Cortex Is Associated with Improved Motor Function after Brain Injury. *Neuron*, 831-840.

Crary, M., Carnaby-Mann, G., & Gorher, M. (2005). Initial psychometric assessment of a functional oral intake scale for dysphagia in stroke patients. *Arch Phys Med Rehabil*, 1516-1520.

Fraser, C., Power, M., Hamdy, S., Rothwell, J., Hobday, D., Hollander, I., et al. (2002). Driving Plasticity in Human Adult Motor Cortex Is Associated with Improved Motor Function after Brain Injury. *Neuron*, 831-840.

Mahoney, F., & Barthel, D. (1965). Functional evaluation: the Barthel Index. *Maryland State Med Journal*, 14: 56-61.

Silbergleit, A., Schultz, L., Jacobson, B., Beardsley, T., & Johnson, A. (2012). The Dysphagia Handicap Index: development and validation. *Dysphagia*, 27 (1): 46-52.

Warnecke, T., Suntrup, S., Teismann, I., K, Hamacher, C., Oelenberg, S., et al. (2013). Standardized Endoscopic Swallowing Evaluation. *Crit Care Med*, (41): 1728-1732.

# Appendices:

Series A appendices are study specific, Series B appendices are general/standard in nature and can be used for multiple studies.

**Series A: Study Specific Documents.**

A.1. Adverse event definition and handling
A.2. Patient Information Sheet and Informed Consent Form
Personal Consultee Information Sheet and Consent Declaration Form
Nominated Consultee Information Sheet and Consent Declaration Form
A.3. Template Investigator Agreement
A.4. Insurance statement
A.5. Structure of database/CRF
A.6. Draft CRFs: data blocks
A.7. Patient data collection flow chart
A.8. Patient Card
A.9. Speech and Language Therapy management plan

**Series B: General Documents (Questionnaires).**

B.1 Dysphagia Severity Ranking Scale (DSRS)
B.2 Functional Oral Intake Score (FOIS)
B.3. Eating Assessment Tool (EAT-10)
B.4 Penetration/Aspiration Score (PAS) Scale
B.5 Severity of Secretion Score (SSS)
B.6 Barthel Index
B.7 Euro-Qol-5D (3L)
B.8 Dysphagia Handicap Index
B.9 Modified Rankin Scale
B.10 National Institute of Health Stroke Scale (NIHSS)
B.11 Glasgow Coma Scale
B.12 Decannulation protocol

If there are non-substantial changes to any of these appendices or required changes by a local authority (e.g. Ethics Committee or Research Office), this would not require a revision of the CIP.

## A.1: Adverse Event (AE) definitions and handling

***Definitions***

| | |
|---|---|
| **ADE** | Adverse Device Effect: AE related to the use of an investigational medical device. This includes:<br>A. any AE resulting from insufficiencies or inadequacies in the instructions for use, the deployment, the implantation, the installation, the operation, or any malfunction of the investigational medical device.<br>B. any event that is a result of a use error or intentional misuse. |
| **AE** | Adverse Event: Any untoward medical occurrence, unintended disease or injury or any untoward clinical signs (including an abnormal laboratory finding) in patients, users or other persons whether or not related to the investigational medical device.<br>This includes:<br>A. events related to the investigational device or the comparator.<br>B. events related to the procedures involved (any procedure in the clinical investigation plan).<br>C. events related to the investigational medical device by users. |
| **SAE** | Serious Adverse Event: AE that:<br>A. led to a death,<br>B. led to a serious deterioration in health that either:<br>1. resulted in a life-threatening illness or injury, or<br>2. resulted in a permanent impairment of a body structure or a body function, or<br>3. required in-patient hospitalization or prolongation of existing hospitalization, or<br>4. resulted in medical or surgical intervention to prevent life threatening illness or injury or permanent impairment to a body structure or a body function. |
| **SADE** | Serious Adverse Device Effect: ADE that has resulted in any of the consequences characteristic of a SAE. |
| **USADE** | Unanticipated serious adverse device effect: AE which by its nature, incidence, severity or outcome has not been identified in the current version of the risk analysis report. |

| | |
|---|---|
| **Anticipated serious adverse device effects** | An AE that is listed as a potential issue in the Risk Analysis of the company.<br><br>The identified anticipated SADES are listed in this Appendix. |
| **Device Deficiency** | An inadequacy of the medical device with respect to its identity, quality, durability, reliability, safety or performance. This includes malfunctions, use errors, and inadequate labelling. Device deficiencies that did not lead to an adverse event but could have led to a medical occurrence :<br><br>a) if either suitable action had not been taken,<br>b) if intervention had not been made, or<br>c) if circumstances had been less fortunate,<br><br>shall be reported to the Ethics Committee. |

***Handling***

During the period of the investigation the handling of adverse events will occur per the guidelines given by the European Commission on clinical investigations[24]. As such new findings/updates in relation to already reported events are considered reportable events.

All SAEs, SADEs, unanticipated adverse effects, device deficiencies (previously called 'incidents') and new findings/updates in relation to already reported events must be reported to the sponsor. In case of death or SAE's requiring urgent medical interventions: by telephone within 24 hours of the investigator becoming aware of the SAE.

The investigator should institute appropriate therapeutic and follow-up measures in accordance with good medical practice but should notify the monitor of such actions and record them in the patient's case report form. Each telephone reported event must be documented in writing to Phagenesis Limited within three (3) working days of the event.

Safety reporting to Ethics Committees, responsibilities and timelines are detailed hereunder. Where national law differs from this CIP, the national law for that country will take precedence.

All SAEs, SADEs and/or UADEs will be followed-up until they are resolved or for 30 days after the patient's participation in the clinical investigation ends.

[24] http://ec.europa.eu/health/medical-devices/files/meddev/2_7_3_en.pdf

| | Who | When | How | To Whom |
|---|---|---|---|---|
| SAE | Investigator<br><br><br><br>Sponsor | Within 3 days of the Investigator becoming aware of the event<br>Within 2 days of sponsor becoming aware of the event | AE report form | (Main) EC for the investigation<br><br><br>CA of country |
| | Sponsor | Within 30 days of sponsor becoming aware of the event. | | All Principal Investigators |
| Device deficiency | Investigator<br><br>Sponsor | Without undue delay<br>Within 7 days of sponsor becoming aware of the event | AE report form | (Main) EC for the investigation<br>CA of country |
| Urgent safety measures | Investigator<br><br><br><br><br><br><br>Sponsor | (i) immediately<br><br>(ii) within 3 days<br><br><br><br><br><br>Within 2 days of sponsor becoming aware of the event | (i) by telephone, followed in writing<br>(ii) notice in writing setting out reasons for the urgent safety measures and the plan for further action | Sponsor<br>(Main) EC for the investigation<br><br><br><br><br><br>CA of country |
| New finding: update of already reported event | Sponsor | Within 7 days of sponsor becoming aware of the event | AE report form | (Main) EC for the investigation<br><br>CA of country |

***Anticipated Adverse Device Effects***

Based on the Investigator Brochure, where the details of an extensive literature search are listed and based on the Instructions for Use, the following warnings and/or contra-indications are mentioned that potentially could lead to AEs which can be considered anticipated:

- difficulty or inability to position the Phagenyx Catheter in patients with anatomical abnormalities that preclude passage of the catheter (or any other feeding tube);

- use of the Phagenyx Catheter is contra-indicated to be used in patients with oral intubation[25], history of oesophageal perforation, stricture or pouch, as use of the device is those patients can lead to complications;

-use of the Phagenyx Catheter should be avoided in patients with cardiac or respiratory condition that might render the insertion of a catheter into the throat unsafe;

- use of the Phagenyx device should be avoided in patients implanted with a pacemaker or a cardioverter defibrillator as interference between electrical stimulation and dysfunction of the device might result;

- when the patient is to receive a magnetic resonance imaging (MRI) scan, the Phagenyx Catheter should not be left in place to prevent generation of electrical current by the magnetic field in the metal leads;

- pharyngeal electrical stimulation should not be carried out in patients receiving oxygen therapy[26] whilst the supply is in place or in operation and be in contact with the electrodes;

- pharyngeal electrical stimulation should not be carried out in female patients who are pregnant or who are nursing;

- when applying PES, distortions or anomalies in electrical encephalogram (EEG) or in electrocardiogram (ECG) recordings might occur when recorded in parallel to the PES by the applied current ;

- the Base Station should be used as indicated, no other devices should at any time be connected to the Base Station than Phagenesis approved USB sticks or catheters;

---

[25] The wording “oral intubation” is intended to indicate those conditions where the presence of equipment for mechanical ventilation would prevent the use of the Phagenyx Catheter and circumstances where electrical stimulation would occur in spaces with high oxygen concentration (please also refer to footnote 26).

[26] It is contraindicated to apply the PES treatment to a patient that is receiving continuous oxygen therapy. In order to treat these patients the supply must be discontinued and the equipment (e.g. a nasal cannula or mask etc.) removed for the duration of treatment. It is the treating physician’s responsibility to ensure that the continuous oxygen is stopped and equipment is removed prior to starting the PES treatment. Patients may be treated with Phagenyx whilst also simultaneously receiving oxygen therapy via a tracheotomy cannula, but the cuff on the cannula should be inflated to prevent oxygen flow near the catheter electrodes.

- damage to the packaging might lead to unsterile catheter products – in such a case the catheter should not be used;

- the catheter is indicated for single patient use – using the product in multiple patients is contraindicated and must be avoided;

- use of the catheter is indicated for a single patient for up to 30 days – thereafter it must be removed and disposed of in clinical waste;

- the electrodes of the catheter should never be handled/touched when the catheter is connected to Base Station – connection to the Base Station should only be done after the catheter has been inserted into the patient;

- the catheter should be checked before and after feeding to ensure there are no leaks;

- if changes in performance of the catheter are observed, it should not be used further;

- connection of the catheter to enteral feeding systems can occur but only via a stepped enteral connector and to enteral feeding sets and/or feeding pumps designed to deliver nutrition via an 8Fr feeding tube.

No additional device related AEs were currently observed in the STEPS study.

In case of emergency, the following contact details should be used:

Name: Jaak Minten
Telephone number : +32 (0) 475-923-149

In addition, the study is executed under the supervision of a physician, who also co-reviews each SAE and ADE. The outcome of each review is documented in the files of the clinical study at Phagenesis.

## A.2: Patient (Personal/Nominated Consultee) Information Sheet and Informed Consent (Declaration) Forms

(Version 1.2**,** UK 10Apr2015) – example serving as a template for local translation and adaption. Local Ethics Committee requirements must be further implemented, if requested.

**(To be printed on the letter headed paper of the hospital)**

**Participant Information Sheet**

*A prospective, single-arm, observational clinical follow-up study on the application of* ***PHA****ryngeal electrical stimulation for treatment of neurogenic* ***D****ysphagia: a* ***E****uropean* ***R****egistry (PHADER).*

We would like to invite you to take part in our Registry also known as a 'study'. Before you decide, you need to understand why it is being done and what it would involve for you. Please take time to read this information carefully and talk to others about it if you wish. A member of our team will go through the information sheet with you and answer any questions you may have.

- **Part 1** tells you the purpose of this study and what will happen to you if you take part.
- **Part 2** gives you more detailed information about the conduct of the study.

Please ask us if anything is not clear or if you would like more information. **Thank you for reading this.**

**Part 1**

**What is the purpose of this study?**

You have been diagnosed with or are suffering from 'neurogenic dysphagia'. This is the medical term for swallowing difficulties that are caused by injury or diseases that affect the brain and nerves controlling swallowing. A variety of conditions such as Stroke, Parkinson's Disease or mechanical ventilation can lead to difficult swallowing. 'Pharyngeal Electrical Stimulation' (PES) may have been offered to you as a treatment for dysphagia. PES-treatment is delivered using a commercially available medical device.

Scientific research on PES shows that it can help improve swallowing function. This previous research has mainly been performed in Stroke patients, but other causes of 'neurogenic dysphagia' can also be treated by the device and may benefit from the treatment.

The purpose of this study, known as a Registry, is to document the regular use of the treatment devices in the 'real-world', so that we can judge the long-term success and safety of them. During four previous studies of PES, the treatment devices were found to have a good safety profile and good performance was achieved in over 200 patients with no device related adverse events observed; as such, the treatment can be described as "low risk". There are no specific or "experimental" parts to this study – all treatments you will receive are part of "standard care". Standard care is not considered "research" and therefore is not part of the study. The collection and entry of data into the Registry is considered "Research" and is why we are asking you to provide consent.

**Why have I been chosen?**

Your care team will/have recommended PES-treatment for your dysphagia and think you would be suitable to take part in this Study.

**Do I have to take part?**

No. You will still be offered the PES-treatment or any other suitable treatment if you decide not to take part. We will describe the study and go through this information sheet. If you agree to take part, we will ask you to sign a Consent Form. You are free to withdraw at any time, without giving a reason. This would not affect the standard of care you are entitled to receive.

**What will happen to me if I take part and what will I have to do?**

Your doctor/care team will carefully check if you are able to take part and if you might benefit from the PES-treatment. For this they will check your medical records in relation to PES and may ask a series of questions related to your condition. Some conditions may make it unsafe for you to receive PES such as having a pacemaker, being pregnant or requiring continuous oxygen therapy. The team will check all of this and let you know if you can take part.

A total of 300 patients will take part in this study, which will take about 36 months in total to be completed. Each patient that takes part in this study will receive the PES-treatment and will have information or 'data' about their condition and swallowing documented in the Registry from the point of consent for up to a maximum time period of three months after finishing the PES-treatment. These data can be collected while you are in the hospital, in a rehabilitation home or in your own home; in the latter case, someone from the medical staff may call or visit you to complete the study documents for that time period.

**Expenses and payments**

There are no expenses for you for taking part in this study, nor will you receive any compensation. You will not be charged for any study treatments.

**What are the alternative treatments available?**

All treatments offered to patients within this study are part of "standard care", only the data collection element of this study is considered "research" and requires your consent. Taking part in this study therefore may not make your health better. Instead of taking part in this study you may choose to continue to get your regular care from your doctor without your data being collected for the study.

**What side effects or risks can I expect from this study?**

In general, all procedures in this study are well tolerated by people. Although the PES-treatment is "low risk", it is not impossible that an unanticipated risk may occur during the clinical study, but the chances of this happening are small and we have taken steps to make sure it is as safe as possible for you to take part. We will watch everyone in the study for any side effects and keep a special close watch on any health event. Specifically the risks and unwanted events that can come about could be listed as follows;

1) *Insertion of tubes into the throat:* The insertion of the treatment tubes through the nose can cause mild but temporary irritation of the nose or throat. Experienced staff will carry out this

procedure to minimise the discomfort. There has been no incidence of harmful complications caused by the insertion of such tubes in previous studies.

2) *Electrical stimulation of the throat:* The electrical stimulation can sometimes cause a moderate warm sensation at the back of the throat but this sensation is not painful.
3) *Your Information:* All data collected from (or about) you during the study will remain strictly confidential and will not identify you in any way. The data will however, be reviewed by people outside of the care team but only authorised people involved with the study will be given access. More details about who has access and how long the data are kept are provided in Part 2 of this information sheet.

**What are the possible benefits of taking part?**

There are no benefits to you in taking part. However, your swallowing function will be assessed in detail during the study period and your participation will contribute to scientific knowledge in this area. Your participation will also improve our understanding of how other patients with swallowing difficulties may be helped. Your swallowing function may improve after the study treatment. If we observe that your swallowing has improved during the study, we will suggest an updated care plan for you as we would routinely for all patients.

**What if something goes wrong?**

Any complaint about the way you have been dealt with during the study or any possible harm you might suffer will be addressed. The detailed information on this is given in Part 2.

**Will my taking part in the study be kept confidential?**

Yes. All the information about your taking part in this study will be kept strictly confidential. More details are included in Part 2.

**This completes Part 1 of the Information Sheet.**

If the information in Part 1 has interested you and you are considering taking part, please read the additional information in Part 2 before making any decision.

## Part 2

**What if relevant new information becomes available?**

You will be informed in case novel information becomes available through the conduct of the study, or if the study concept would change by which your study participation could be jeopardised.

**What will happen if I don't want to carry on with the study or the study stops?**

As your study participation is completely voluntary, you may withdraw from the study at any time without any consequence to your continued care. If you leave the study, no more information about you will be collected for the study. All the information collected before you left the study will still be used. The same also applies if the sponsor decides to stop the clinical investigation early.

**What if there is a problem?**

If you have a concern about any aspect of this study, you should first speak to the researchers who will do their best to answer your questions. If you remain unhappy and wish to make a formal complaint about the way you have been dealt with during the study, you can do this by contacting the Patient Advice & Liaison Service (PALS) at [Insert relevant details]:

- Telephone: [Insert relevant details]
- E-mail: [Insert relevant details]

There are no expenses or compensation for taking part in this study. NHS indemnity is in place during the conduct of this study and you will be insured by the sponsor in the event of an injury arising from taking part in this clinical investigation. For any injury caused by taking part in this study, we will provide compensation in accordance with the guidelines of the Association of the British Healthcare Industry (ABHI). We will pay compensation where the injury resulted from i) the device being tested or administered as part of the research or ii) any test or procedure you received as part of the study.

Compensation will be paid regardless of whether you are able to prove that the company has been negligent or that the product is defective (please ask if you would like more information on this). We would not be bound by these guidelines to pay compensation where i) the injury resulted from a device or procedure outside the study protocol and/or ii) the protocol was not followed.

**Will my information be kept private and confidential?**

Yes. All clinical data that is collected from you during the study will remain strictly confidential. In order to use and analyse your data, we will need to access to your clinical records. Only people involved with the study, such as the clinical staff, those assigned by the study sponsor, and potentially some from government regulatory authorities (Europe, U.S.A., Asia) who collect information to check that the study is being run properly will have access to your data. The data collected will be maintained for a long time (more than 2 years as required by regulation), but will remain strictly confidential and none of your personal data will be disclosed to inappropriate parties. By taking part in the study, and by signing the Informed Consent Form, you grant these persons/organisations access to your personal medical records and data. Please note that all your medical records are kept strictly confidential and that outside the hospital, only anonymised data is used and stored securely: this means your information will be labelled with a code/number and no information with your name or other identifiable information will leave the hospital.

**Who is funding this study?**

This study is set-up and sponsored by the manufacturer of the PES-treatment, Phagenesis Limited, located in Manchester, United Kingdom. The Chief Investigator, Professor Shaheen Hamdy, is not an employee of the company but is on the board as Chief Scientific Officer and is a shareholder. None of the research team members receive any payment beyond their normal salary for conducting the study.

**Who has reviewed this study?**

This study was given a favourable opinion by the East of England - Cambridge Central Research Ethics Committee (reference no: 15/EE/0047).

**Contact details**

The sponsor's representative in this hospital is:

_____________________________________________ (name of investigator, title)

_____________________________________________ (telephone number)

He/she is responsible for the correct conduct of the clinical study at this hospital. If you wish to take part or have any remaining questions about the study or about your rights, or in the case of an injury arising from taking part in this study, please contact this person.

Strictly Confidential

(Version 1.2, UK 10Apr2015) – example serving as a template for local translation and adaption. Local Ethics Committee requirements must be further implemented, if requested.

(To be printed on the letter headed paper of the hospital)

**Centre Number:** ______________________ **Study Number:** ______________________

**Patient Screening ID for Study:** ______________________

**PATIENT INFORMED CONSENT FORM**

**Study Title:** A prospective, single-arm, observational clinical follow-up study on the application of PHAryngeal electrical stimulation for treatment of neurogenic Dysphagia: a European Registry (PHADER)

**Name of Investigator:** [input name of PI] **Please initial Box**

| | |
|---|---|
| 1. I confirm that I have read and understand the information sheet dated 10Apr2015 (Version 1.2, UK) for the above study. I have had the opportunity to consider the information, ask questions and have had these answered satisfactorily. | |
| 2. I understand that my participation is voluntary and that I am free to withdraw at any time without giving any reason, without my medical care or legal rights being affected. | |
| 3. I understand that relevant sections of my medical notes and data collected during the study, may be looked at by individuals outside of the care team, from/on behalf of Phagenesis Limited, from regulatory authorities, the hospital, where it is relevant to my taking part in this research. I give permission for these individuals to have access to my records and data. | |
| 4. I understand that data collected about me in this study may be used as described in the information sheet, including transfer to countries outside the European Union for submission to regulatory bodies (for example the Food and Drug Administration of America). | |
| 5. I agree to take part in this study. | |

| **Name of Patient** | **Date** | **Signature** |
|---|---|---|

| **Name of Person taking consent** | **Date** | **Signature** |
|---|---|---|

(Version 1.2**,** UK 10Apr2015) – example serving as a template for local translation and adaption.
Local Ethics Committee requirements must be further implemented, if requested.

**(To be printed on the letter headed paper of the hospital)**

**Personal Consultee Information Sheet**

*A prospective, single-arm, observational clinical follow-up study on the application of **PHA**ryngeal electrical stimulation for treatment of neurogenic **D**ysphagia: a **E**uropean **R**egistry (PHADER).*

**Invitation paragraph**

You are being invited to act as a 'personal consultee' for a relative/friend who is unable to make a decision for themselves. You are being asked to advise the researcher about your relative/friend's wishes and feelings as to whether they themselves would have wished to join this research. It is important for you to understand what it means to be a personal consultee, as well as why the research is being done and what it will involve. Please take time to read this information carefully and talk to others about the study if you wish. Ask us if there is anything that is not clear or if you would like more information. Take time to decide whether or not you wish be a personal consultee.

**What does it mean to be a personal consultee?**

A personal consultee is someone (typically a relative/friend) who knows a person with a mental incapacity well and is willing and able to offer an opinion to the research/care team as to what that incapacitated person's wishes would have been if they did not have a mental incapacity. You do not have to act as the personal consultee if you do not want to. If you decide to act as the personal consultee, you will be asked to sign a Personal Consultee Declaration Form.

If you think that your relative/friend would not have wanted to take part, then the researchers will respect this. Please remember that you are not being asked for your personal views on the research but consider only what your relative/friend's wishes would have been were they being asked to take part in this research. Think about the broad aims of the research, the risks and benefits and what taking part will mean for your relative/friend. At any stage, you can advise the researcher that your opinion about their possible wishes about being in the study have changed and they may no longer wish to remain in the study.

**Why have I been asked to be a personal consultee?**

You may have been chosen because you know this person well and they would trust you to help with this decision.

Please take time to read this information carefully and talk to others about the study if you wish. Ask us if there is anything that is not clear or if you would like more information. Take time to decide whether you wish be a personal consultee.

- **Part 1** tells you the purpose of this study and what will happen to your relative/friend.
- **Part 2** gives you more detailed information about the conduct of the study.

Please ask us if anything is not clear or if you would like more information. **Thank you for reading this.**

## Part 1

**What is the purpose of this study?**

Your relative/friend has been diagnosed with or is suffering from 'neurogenic dysphagia'. This is the medical term for swallowing difficulties that are caused by injury or diseases that affect the brain and nerves controlling swallowing. A variety of conditions such as Stroke, Parkinson's Disease or nerve damage from mechanical ventilation can lead to difficult swallowing. 'Pharyngeal Electrical Stimulation' (PES) has been offered to your friend/relative as a treatment for dysphagia. PES-treatment is delivered using a commercially available medical device.

Scientific research on PES shows that it can help improve swallowing function. This previous research has mainly been performed in Stroke patients, but other causes of 'neurogenic dysphagia' can also be treated by the device and may benefit from the treatment. This study is designed to help document the potential benefit (or lack of benefit) in a larger variety of patients treated for swallowing difficulty than has been done thus far.

The purpose of this study, known as a Registry, is to document the regular use of the treatment devices in the 'real-world', so that we can judge the long-term success and safety of them. During four previous studies of PES, the treatment devices were found to have a good safety profile and good performance was achieved in over 200 patients with no device related adverse events observed; as such, the treatment can be described as "low risk". There are no specific or "experimental" parts to this study – all treatments your friend/relative will receive will be recommended by their care team and are part of "standard care". Standard care is not considered "research" and therefore is not part of the study. The collection and entry of data into the Registry is considered 'Research" and is why we are asking you to provide consent as the personal consultee.

**Why has my relative/friend been chosen?**

Your relative/friend has been recommended PES-treatment for their dysphagia and the research/care team have identified them as someone who would be suitable to take part in this Study.

**Does my relative/friend have to take part?**

No. Your friend/relative will still be offered the PES-treatment or any other suitable treatment if you decide they should not take part in this study. Because your friend/relative is unable to decide for themselves, we are asking for advice on their wishes from other people. If you want to help give advice, you can keep this information sheet. We will describe the study and go through this information sheet with you. If you agree that your friend/relative would wish to take part, we will record your advice on a Personal Consultee Declaration Form. At anytime, you or your relative/friend can decide to withdraw from study participation, without giving a reason. This will not affect the standard of care they are entitled to receive.

**What will happen to my relative/friend if they take part and what will they have to do?**

The research/care team will carefully check if they are able to take part and if they might benefit from the PES-treatment. For this they will check their medical records in relation to PES. Some conditions that may make it unsafe for your relative/friend to receive PES such as having a

pacemaker, being pregnant or requiring continuous oxygen therapy. The team will check all of this and let you know if your relative/friend can take part.

A total of 300 patients will take part in this Study, which will take about 36 months in total to be completed. Each patient that takes part in the study will receive the PES-treatment and will have information or 'data' about their condition and swallowing documented in the Registry from the point of consent for up to a maximum time period of three months after finishing the PES-treatment. These data can be collected while your relative/friend is in the hospital, in a rehabilitation home or in their own home; in the latter case, someone from the medical staff may call or visit your relative/friend to complete the study documents for that time period.

**Expenses and payments**

There are no expenses for taking part in this study, nor will your relative/friend receive any compensation. They will not be charged for any study treatments.

**What are the alternative treatments available?**

All treatments offered to patients within this study are part of "standard care", only the data collection element of this study is considered "research" and requires consent. Taking part in this study therefore may not make your relative/friends health better. Instead of taking part in this study they can continue to get receive regular care from their doctor without their data being collected for the study.

**What side effects or risks can my relative/friend expect from this study?**

In general, all procedures in this study are well tolerated by people. Although the PES-treatment is "low risk", it is not impossible that an unanticipated risk may occur during the clinical study, but the chances of this happening are small and we have taken steps to make sure it is as safe as possible for your relative/friend to take part. We will watch everyone in the study for any side effects and keep a special close watch on any health event. Specifically the risks and unwanted events that can come about could be listed as follows;

1) *Insertion of tubes into the throat:* The insertion of the treatment tubes through the nose can cause mild but temporary irritation of the nose or throat. Experienced staff will carry out this procedure to minimise the discomfort. There has been no incidence of harmful complications caused by the insertion of such tubes in previous studies.
2) *Electrical stimulation of the throat:* The electrical stimulation can sometimes cause a moderate warm sensation at the back of the throat but this sensation is not painful.
3) *Patient Information:* All data collected from (or about) patients during the study will remain strictly confidential and will not identify them in any way. The data will however, be reviewed by people outside of the care team but only authorised people involved with the study will be given access. More details about who has access and how long the data are kept are provided in Part 2 of this information sheet.

**What are the possible benefits of taking part?**

There are no benefits to your relative/friend in taking part. However, their swallowing function will be assessed in detail during the study period and their participation will contribute to scientific

knowledge in this area. Their participation will also improve our understanding of how other patients with swallowing difficulties may be helped. Their swallowing function may improve after the study treatment. If we observe that their swallowing has improved during the study, the research/care team will suggest updating their care plan as they would routinely for all patients.

**<u>What if something goes wrong?</u>**

Any complaint about the way your relative/friend has been dealt with during the study or any possible harm your relative/friend might suffer will be addressed. The detailed information on this is given in Part 2.

**<u>Will my relative/friend taking part in the study be kept confidential?</u>**

Yes. All the information about your relative/friend taking part in this study will be kept strictly confidential. More details are included in Part 2.

**This completes Part 1 of the Information Sheet.**

If, after reading the information in Part 1 you feel that your relative/friend would be interested in taking part in this study, please read the additional information in Part 2 before making any decision.

## Part 2

**<u>What if relevant new information becomes available?</u>**

You will be informed in case novel information becomes available through the conduct of the study, or if the study concept would change by which your relative/friend's study participation could be jeopardised.

**<u>What will happen if I don't want my relative/friend to carry on with the study or the study stops?</u>**

As study participation is completely voluntary, you may withdraw your relative/friend from the study at any time without any consequence to their continued care. If your relative/friend leaves the study, no more information about them will be collected for the study. All the information collected before they left the study will still be used. The same also applies if the sponsor decides to stop the clinical investigation early.

**<u>What if there is a problem?</u>**

If you have a concern about any aspect of this study, you should first speak to the researchers who will do their best to answer your questions. If you remain unhappy and wish to make a formal complaint about the way your relative/friend has been dealt with during the study, you can do this by contacting the Patient Advice & Liaison Service (PALS) at [Insert relevant details]:

- Telephone: [Insert relevant details]
- E-mail: [Insert relevant details]

There are no expenses or compensation for taking part in this study. NHS indemnity is in place during the conduct of this study and your relative/friend will be insured by the sponsor in the event of an injury arising from taking part in this clinical investigation. For any injury caused by taking part in this study, we will provide compensation in accordance with the guidelines of the Association of the

British Healthcare Industry (ABHI). We will pay compensation where the injury resulted from i) the device being tested or administered as part of the research or ii) any test or procedure they received as part of the study.

Compensation will be paid regardless of whether you are able to prove that the company has been negligent or that the product is defective (please ask if you would like more information on this). We would not be bound by these guidelines to pay compensation where i) the injury resulted from a device or procedure outside the study protocol and/or ii) the protocol was not followed.

**Will my relative/friend's information be kept private and confidential?**

Yes. All clinical data that is collected from your relative/friend during the study will remain strictly confidential. In order to use and analyse their data, we will need to access their clinical records. Only people involved with the study, such as the clinical staff, those assigned by the study sponsor, and potentially some from government regulatory authorities (Europe, U.S.A., Asia) who collect information to check that the study is being run properly will have access to the data. The data collected will be maintained for a long time (more than 2 years as required by regulation), but will remain strictly confidential and none of your their personal data will be disclosed to inappropriate parties. By taking part in the study, and by signing the Personal Consultee Declaration Form, you grant these persons/organisations access to your relative/friend's personal medical records and data. Please note that all medical records are kept strictly confidential and that outside the hospital, only anonymised data is used and stored securely: this means their information will be labelled with a code/number and no information with their name or other identifiable information will leave the hospital.

**Who is funding this study?**

This study is set-up and sponsored by the manufacturer of the PES-treatment, Phagenesis Limited, located in Manchester, United Kingdom. The Chief Investigator, Professor Shaheen Hamdy, is not an employee of the company but is on the board as Chief Scientific Officer and is a shareholder. None of the research team members receive any payment beyond their normal salary for conducting the study.

**Who has reviewed this study?**

This study was reviewed and given a favourable opinion by the East of England - Cambridge Central Research Ethics Committee (reference no: 15/EE/0047). **Contact details**

The sponsor's representative in this hospital is:

_____________________________________________ (name of investigator, title)

_____________________________________________ (telephone number)

He/she is responsible for the correct conduct of the clinical study at this hospital. If you agree that your relative/friend would wish to take part or have any remaining questions about the study or about your/their rights, or in the case of an injury arising from taking part in this study, please contact this person.

(Version 1.2, UK 10Apr2015) – example serving as a template for local translation and adaption. Local Ethics Committee requirements must be further implemented, if requested.

(To be printed on the letter headed paper of the hospital)

**Centre Number:** ______________________ **Study Number:** ______________

**Patient Screening ID for Study:** ______________________

**PERSONAL CONSULTEE DECLARATION FORM**

**Study Title:** A prospective, single-arm, observational clinical follow-up study on the application of PHAryngeal electrical stimulation for treatment of neurogenic Dysphagia: a European Registry (PHADER)

**Name of Investigator:** [input name of PI] **Please initial Box**

| | |
|---|---|
| 1. I confirm that I have read and understand the personal consultee information sheet dated 10Apr2015 (Version 1.2, UK) for the above study. I have had the opportunity to consider the information, ask questions and have had these answered satisfactorily. | |
| 2. I understand what participation involves, that participation is voluntary and that I am free to withdraw my relative/friend at any time without giving any reason, without their medical care or legal rights being affected. In my opinion they would not object to taking part in this study. | |
| 3. I understand that relevant sections of their medical notes and data collected during the study, may be looked at by individuals outside of the care team, from/on behalf of Phagenesis Limited, from regulatory authorities, the hospital, where it is relevant to their taking part in this research. I give permission for these individuals to have access to their records and data. | |
| 4. I understand that data collected from my relative/friend in this study may be used as described in the information sheet, including transfer to countries outside the European Union submission to regulatory bodies (for example the Food and Drug Administration of America). | |
| 5. I understand that the identified patient (my relative/friend) would wish to take part in this study. | |

**Name of Personal Consultee** **Date** **Signature**

**Relationship of consultee to patient** **Consultee contact number**

**Name of Person taking consent** **Date** **Signature**

(Version 1.2**,** UK 10Apr2015) – example serving as a template for local translation and adaption. Local Ethics Committee requirements must be further implemented, if requested.

**(To be printed on the letter headed paper of the hospital)**

**Nominated Consultee Information Sheet**

*A prospective, single-arm, observational clinical follow-up study on the application of* ***PHA****ryngeal electrical stimulation for treatment of neurogenic* ***D****ysphagia: a* ***E****uropean* ***R****egistry (PHADER)*

**Invitation paragraph**

You are being invited to act as a 'nominated consultee' for someone who is unable to make a decision for themselves. You are being asked to advise the researcher about this patient's wishes and feelings as to whether they themselves would have wished to join this research. It is important for you to understand what it means to be a nominated consultee, as well as why the research is being done and what it will involve. Please take time to read this information carefully and talk to others about the study if you wish. Ask us if there is anything that is not clear or if you would like more information. Take time to decide whether or not you wish be a nominated consultee.

**What does it mean to be a nominated consultee?**

A nominated consultee is someone (typically a Health Care Professional that is independent of the study) appointed by the research/care team who is able to offer an opinion, based on risk of harm, as to what that incapacitated patient's wishes would have been if they did not have a mental incapacity. You do not have to act as the nominated consultee if you do not want to. If you decide to act as the nominated consultee, you will be asked to sign a Nominated Consultee Declaration Form.

If you think that this patient would not have wanted to take part, then the researchers will respect this. Please remember that you are not being asked for your personal views on the research but consider only what the patient's wishes would have been were they being asked to take part in this research. Think about the broad aims of the research, the risks and benefits and what taking part will mean for this patient. At any stage, you can advise the researcher that your opinion about the patient's possible wishes about being in the study have changed and they may no longer wish to remain in the study.

**Why have I been asked to be a nominated consultee?**

You may have been chosen because you are a member of the care team who looks after this patient (such as member of care home staff, GP, health care professional), and you have the patient's best interests in mind. Or you may be a member of a 'Board of Nominated Consultees' who has received training and help for taking on this role.

Please take time to read this information carefully and talk to others about the study if you wish. Ask us if there is anything that is not clear or if you would like more information. Take time to decide whether you wish be a nominated consultee.

**Part 1** tells you the purpose of this study and what will happen to your patient.

**Part 2** gives you more detailed information about the conduct of the study.

Please ask us if anything is not clear or if you would like more information. **Thank you for reading this.**

## Part 1

### What is the purpose of this study?

The patient in question has been diagnosed with or is suffering from 'neurogenic dysphagia'. This is the medical term for swallowing difficulties that are caused by injury or diseases that affect the brain and nerves controlling swallowing. 'Pharyngeal Electrical Stimulation' (PES) has been offered to this patient as a treatment for dysphagia. PES-treatment is delivered using a commercially available medical device.

Scientific research on PES shows that it can help improve swallowing function. This previous research has mainly been performed in Stroke patients, but other causes of 'neurogenic dysphagia' can also be treated by the device and may benefit from the treatment. This study is designed to help document the potential benefit (or lack of benefit) in a larger variety of patients treated for swallowing difficulty than has been done thus far.

The purpose of this study, known as a Registry, is to document the regular use of the treatment devices in the 'real-world', so that we can judge the long-term success and safety of them. During four previous studies of PES, the treatment devices were found to have a good safety profile and good performance was achieved in over 200 patients with no device related adverse events observed; as such, the treatment can be described as "low risk". There are no specific or "experimental" parts to this study – all treatments the patient will receive will be recommended by their care team and are part of "standard care". Standard care is not considered "research" and therefore is not part of the study. The collection and entry of data into the Registry is considered "Research" and is why we are asking you to provide consent as the nominated consultee.

### Why has this patient been chosen?

This patient will/has been recommended PES-treatment for their dysphagia and the research/care team have identified them as someone who would be suitable to take part in this Study.

### Do they have to take part?

No. Patients will still be offered the PES-treatment or any other suitable treatment if you decide they should not take part in this study. Because this patient is unable to decide for themselves, we are asking for advice from other people. If you want to help give advice, you can keep this information sheet. If you agree that this patient should take part, we will record your advice on a Nominated Consultee Declaration Form. At anytime, you or the patient can decide to withdraw from study

participation, without giving a reason. This will not affect the standard of care they are entitled to receive.

**<u>What will happen if they take part and what will they have to do?</u>**

The research/care team will carefully check if the patient is able to take part in this study and if they might benefit from the PES-treatment. For this they will check the patient's medical records in relation to PES. Some conditions that may make it unsafe for patients to receive PES such as having a pacemaker, being pregnant or requiring continuous oxygen therapy. The research/care team will check all of this and let you know if the patient can take part.

A total of 300 patients will take part in this Study, which will take about 36 months in total to be completed. Each patient that takes part in the study will receive the PES-treatment and will have information or 'data' about their condition and swallowing documented in the Registry from the point of consent up to a maximum time period of three months after finishing the PES-treatment. These data can be collected while the patient is in the hospital, in a rehabilitation home or in their own home; in the latter case, someone from the medical staff may call or visit the patient to complete the study documents for that time period.

**<u>Expenses and payments</u>**

There are no expenses for taking part in this study, nor will the patient receive any compensation. They will not be charged for any study treatments.

**<u>What are the alternative treatments available?</u>**

All treatments offered to patients within this study are part of "standard care", only the data collection element of this study is considered "research" and requires consent. Taking part in this study therefore may not make the patient's health better. Instead of taking part in this study they can continue to get the regular care from their doctor without their data being collected for the study.

**<u>What side effects or risks can be expected from this study?</u>**

In general, all procedures in this study are well tolerated by patients. Although the PES-treatment is "low risk", it is not impossible that an unanticipated risk may occur during the clinical study, but the chances of this happening are small and we have taken steps to make sure it is as safe as possible for patients to take part. We will watch everyone in the study for any side effects and keep a special close watch on any health event. Specifically the risks and unwanted events that can come about could be listed as follows;

1) <u>*Insertion of tubes into the throat:*</u> The insertion of the treatment tubes through the nose can cause mild but temporary irritation of the nose or throat. Experienced staff will carry out this procedure to minimise the discomfort. There has been no incidence of harmful complications caused by the insertion of such tubes in previous studies.

2) *Electrical stimulation of the throat:* The electrical stimulation can sometimes cause a moderate warm sensation at the back of the throat but this sensation is not painful.
3) *Patient Information:* All data collected from (or about) patients during the study will remain strictly confidential and will not identify them in any way. The data will however, be reviewed by people outside of the care team but only authorised people involved with the study will be given access. More details about who has access and how long the data are kept are provided in Part 2 of this information sheet.

**What are the possible benefits of taking part?**

There are no benefits to the patient in taking part. However, their swallowing function will be assessed in detail during the study period and their participation will contribute to scientific knowledge in this area. Their participation will also improve our understanding of how other patients with swallowing difficulties may be helped. Their swallowing function may improve after the study treatment. If we observe that their swallowing has improved during the study, the research/care team will suggest updating the care plan as they would routinely for all patients.

**What if something goes wrong?**

Any complaint about the way the patient has been dealt with during the study or any possible harm they might suffer will be addressed. The detailed information on this is given in Part 2.

**Will their taking part in the study be kept confidential?**

Yes. All the patient's information about taking part in this study will be kept strictly confidential. More details are included in Part 2.

**This completes Part 1 of the Information Sheet.**

If, after reading the information in Part 1, you feel that participating in the study would be in the patient's best interest, please read the additional information in Part 2 before making any decision.

**Part 2**

**What if relevant new information becomes available?**

You will be informed in case novel information becomes available through the conduct of the study, or if the study concept would change by which the patient's study participation could be jeopardised.

**What will happen if I think the patient would not want to carry on with the study or the study stops?**

As study participation is completely voluntary, you may withdraw the patient from the study at any time without any consequence to their continued care. If they leave the study, no more information

about them will be collected for the study. All the information collected before they left the study will still be used. The same also applies if the sponsor decides to stop the clinical investigation early.

**What if there is a problem?**

If you have a concern about any aspect of this study, you should first speak to the researchers who will do their best to answer your questions. If you remain unhappy and wish to make a formal complaint about the way the patient has been dealt with during the study, you can do this by contacting the Patient Advice & Liaison Service (PALS) at [Insert relevant details]:

- Telephone: [Insert relevant details]
- E-mail: [Insert relevant details]

There are no expenses or compensation for taking part in this study. NHS indemnity is in place during the conduct of this study and patients will however be insured by the sponsor in the event of an injury arising from taking part in this clinical investigation. For any injury caused by taking part in this study, we will provide compensation in accordance with the guidelines of the Association of the British Healthcare Industry (ABHI). We will pay compensation where the injury resulted from i) the device being tested or administered as part of the research or ii) any test or procedure they received as part of the study.

Compensation will be paid regardless of whether you are able to prove that the company has been negligent or that the product is defective (please ask if you would like more information on this). We would not be bound by these guidelines to pay compensation where i) the injury resulted from a device or procedure outside the study protocol and/or ii) the protocol was not followed.

**Will their information be kept private and confidential?**

Yes. All clinical data that is collected from the patient during the study will remain strictly confidential. In order to use and analyse their data, we will need to access to their clinical records. Only people involved with the study, such as the clinical staff, those assigned by the study sponsor, and potentially some from government regulatory authorities (Europe, U.S.A., Asia) who collect information to check that the study is being run properly will have access to the data. The data collected will be maintained for a long time (more than 2 years as required by regulation), but will remain strictly confidential and none of their personal data will be disclosed to inappropriate parties. By taking part in the study, and by signing the Nominated Consultee Declaration Form, you grant these persons/organisations access to the patient's personal medical records and data. Please note that all medical records are kept strictly confidential and that outside the hospital, only anonymised data is used and stored securely: this means their information will be labelled with a code/number and no information with their name or other identifiable information will leave the hospital.

**Who is funding this study?**

This study is set-up and sponsored by the manufacturer of the PES-treatment, Phagenesis Limited, located in Manchester, United Kingdom. The Chief Investigator, Professor Shaheen Hamdy, is not an employee of the company but is on the board as Chief Scientific Officer and is a shareholder. None of the research team members receive any payment beyond their normal salary for conducting the study.

**Who has reviewed this study?**

This study was reviewed and given a favourable opinion by the East of England - Cambridge Central Research Ethics Committee (reference no: 15/EE/0047).

**Contact details**

The sponsor's representative in this hospital is:

_____________________________________________ (name of investigator, title)

_____________________________________________ (telephone number)

He/she is responsible for the correct conduct of the clinical study at this hospital. If you agree that it is in the best interest of the patient and that they would wish to take part or have any remaining questions about the study or about their your/rights, or in the case of an injury arising from taking part in this study, please contact this person.

**PHADER European Registry**

**Strictly Confidential**

(Version 1.2, UK 10Apr2015) – example serving as a template for local translation and adaption. Local Ethics Committee requirements must be further implemented, if requested.

(To be printed on the letter headed paper of the hospital)

**Centre Number:** ______________________ **Study Number:** ______________

**Patient Screening ID for Study:** ______________________

**NOMINATED CONSULTEE DECLARATION FORM**

**Study Title:** A prospective, single-arm, observational clinical follow-up study on the application of PHAryngeal electrical stimulation for treatment of neurogenic Dysphagia: a European Registry (PHADER)

**Name of Investigator:** [input name of PI] **Please initial Box**

| | |
|---|---|
| 1. I confirm that I have read and understand the nominated consultee information sheet dated 10Apr2015 (Version 1.2, UK) for the above study. I have had the opportunity to consider the information, ask questions and have had these answered satisfactorily. | |
| 2. I understand what participation involves, that participation is voluntary and that I am free to withdraw the patient at any time without giving any reason, without their medical care or legal rights being affected. In my opinion the patient would not object to taking part in this study. | |
| 3. I understand that relevant sections of the patient's medical notes and data collected during the study, may be looked at by individuals outside of the care team, from/on behalf of Phagenesis Limited, from regulatory authorities, the hospital, where it is relevant to their taking part in this research. I give permission for these individuals to have access to their records and data. | |
| 4. I understand that data collected from them in this study may be used as described in the information sheet, including transfer to countries outside the European Union submission to regulatory bodies (for example the Food and Drug Administration of America). | |
| 5. I understand that the identified patient would wish to take part in this study. | |

**Name of Nominated Consultee** **Date** **Signature**

**Relationship of consultee to patient** **Consultee contact number**

**Name of Person taking consent** **Date** **Signature**

## A.3: Investigator Agreement: Standard Template

Phagenesis has a standard UK version of the Investigator Agreement and has this version translated in different European languages. Together with the CIP, the appropriate template translation or the English version of the agreement is added to the dossier.

The Investigator Agreement comprises two parts:

- One part specifies the duties of sponsor and investigator
- The second part specifies the compensation that is due for completion of clinical study activities.

On the contrary, if a local hospital investigator agreement template is available that can be approved by Phagenesis Limited's legal department, then preferably that agreement will be used as part of the study.

## A.4: Evidence of Clinical Investigation Insurance

Phagenesis Limited has brought the Phagenyx devices onto the market: these bear the CE mark and the company is liable for the product's performance.

The PHADER is a post-market release follow-up study, without any specific study related procedure that is not part of standard practice. The only protocol requirement is to collect specific clinical data via questionnaires and without the conduct of any specific test. As such the patient is not exposed to any additional risk other than when treated according to regular practice.

Phagenesis Limited recognises its liability in law to compensate for any injury sustained by a patient as a result of his/her participation in this clinical investigation in accordance with the guidelines of the Association of the British Healthcare Industry (ABHI) for any patients entered into the clinical investigation in the United Kingdom.

Outside the United Kingdom, Phagenesis agrees to abide by the local guidelines in that country.

## A.5: Structure of Data Forms/Database

| CRF identification | First Tier - Obligatory | Second Tier - Optional | Use of standardized patient questionnaires |
|---|---|---|---|
| Pre-study site information | X | | |
| Patient Data Form<br>3 = general<br>3A = subgroup 1<br>3B = subgroup 2<br>3C = subgroup 3<br>3D = subgroup 4<br>3E = subgroup 5 | X | X | PAS, DSRS, FOIS, BI, EuroQol-5D,DHI, EAT-10, SSS<br><br>For subgroups, special forms are to be used to assess disease severity such as NIHSS, mRS, GCS etc. |
| First Treatment Form | X | | |
| Additional treatment forms | X | X | |
| First-Follow-up form 1 (24-72 hrs) | X | X | PAS, DSRS, FOIS |
| Pre-Hospital form = Follow-up form 2 (7 days → hospital discharge or ≤ 21 days whichever comes first) | X | | PAS, DSRS, FOIS, BI, EuroQol-5D, DHI, EAT-10, SSS |
| 3 month Follow-up form = follow-up form 3 | X | X | PAS, DSRS, FOIS, BI, EuroQol-5D, DHI, EAT-10, SSS |
| Adverse Event Form | X | | |
| Arrival at other ward Form | X | | |
| Unscheduled follow-up form | | X | |
| Lost-to-Follow-up Form | X | | |
| Post-study site information | X | | |

A tier 1 and tier 2 data collection system is installed. The first tier is obligatory and applies for all patients, the second tier is voluntary but the investigator will decide upfront which level of data collection they wish to implement at their investigation centre. Investigational centres will continue to collect their chosen level of data collection (tier 1 vs. tier 2), unless the change of preference of choice is requested and approved by Phagenesis.

# A.6: Case Report Form Construction

## *a. CRF 01 - Site information form (Tier 1)*

| Category | Tier one - Mandatory | Tier two - Optional | Query trigger |
|---|---|---|---|
| Investigator | Name<br>Contact details | | |
| Co-investigator | Name<br>Contact details | | |
| SLT | Name<br>Contact details | | |
| Co-SLT | Name<br>Contact details | | |
| Clinical coordinator | Name<br>Contact details | | |
| Other | Name<br>Contact details | | |
| Pre-study Appreciation of Dysphagia assessments and Rx[27]<br><br>Investigator<br><br>SLT | Diagnostic method: VFS[28] / FEES[29] / Bedside / DSRS[30] / other?) / Number of PES[31] Rx already delivered<br><br>Rx appreciation / Selection decisions<br><br>SLT[32]-Rx: VAS<br>E-Stim: VAS[33]<br>SLT-Rx: VAS<br>E-Stim: VAS | | Method should remain similar throughout the study. |
| Reimbursement for dysphagia Rx | Hospital own budget?<br>Department budget?<br>Health Insurance? | | |
| Reimbursement for PES Rx | Hospital own budget?<br>Department budget?<br>Health Insurance? | | |
| Ease of Use | Rx / IFU[34] / Catheter | | |
| Pre-study Appreciation | VFS / FEES / Bedside / DSRS / | | During conduct of study, |

[27] Rx: abbreviation for therapy/treatment.
[28] VFS = Videofluoroscopy.
[29] FEES = Fibreoptic endoscopic evaluation of swallowing.
[30] DSRS = Dysphagia Severity Ratings Scale.
[31] PES = Pharyngeal Electrical Stimulation.
[32] SLT = Speech and Language Therapist.
[33] VAS: visual analogue scale to assess appreciation of therapy.
[34] IFU = Instructions For Use.

| | | | |
|---|---|---|---|
| of Dysphagia assessments and Rx<br><br><br><br><br><br>Investigator<br><br>SLT | other? / Number of PES Rx already delivered<br><br>Rx appreciation / Selection decisions<br><br>SLT-Rx: VAS<br>E-Stim: VAS[35]<br>SLT-Rx: VAS<br>E-Stim: VAS | | data of pre-assessment are not visible till after closure of study to avoid bias. |

***b. CRF 02 - Patient data form (Tier 1)***

| **Category** | **Tier one – Mandatory** | **Tier two - Optional** | **Query trigger** |
|---|---|---|---|
| Patient Identification and Demographics | Patient ID / Year of Birth / Gender / Weight / Fat percentage | | |
| Enrolment criteria | Does patient meet all inclusion criteria?<br>Does patient fail to meet any of the exclusion criteria?<br><br><br>Informed consent form signed? | | Warning when criteria are not met appropriately.<br>Exclude from access to remaining of CRFs.<br>Automatic email to sponsor<br>If all OK → open access to CRFs. |
| Patient Group Classification | Group a / b / c / d / e? | | |

***CRF 03 - Patient Baseline form (Tier 1 & 2)***

| **Category** | **Tier one – Mandatory** | **Tier two - Optional** | **Query trigger** |
|---|---|---|---|
| Patient Identification and Demographics | Patient ID / Date of assessment / Weight | | |
| Facial anatomical data | Distance nose/ears/pharynx (cm/inches) | | |
| Medical history | Date of admission to hospital<br>Reason for admission | Aetiology and severity of underlying neurogenic disease<br>Disease specific treatments | |
| Dysphagia signs and symptoms | List of symptoms | | |
| Comorbidities | List of comorbidities | Extended list of comorbidities | |

[35] VAS: visual analogue scale to assess appreciation of therapy.

| | | | |
|---|---|---|---|
| General physical condition | Level of disability in arms / legs / capacity to walk / requirement of aids? | Current presentation (disease specific) | |
| Dysphagia Severity | Diagnostic method:<br>VFS / FEES / Bedside / DSRS / other?<br>PAS[36]-score<br>DSRS-score | | Warning if FOIS or DSRS score are not completed. |
| Questionnaires | DHI[37] / FOIS[38] / SSS[39] / EQ-5D[40] / EAT-10[41] | Additional disease specific questionnaires | |
| Feeding status | Eating Orally +/_ supervision?<br>Duration of feeding session<br>NG[42]<br>PEG[43]/RIG[44]<br>Awaiting PEG/RIG/NG<br>Awaiting surgical intervention | | |
| Ventilation status | Patient self-breathing?<br>Nasal Oxygen support? / Date of instalment?<br>Ventilation support? / Via? / Date of instalment?<br>Tracheotomy for dysphagia only?<br>Previously ventilated? / Start date/ Date weaning completed? | | Warning: EPS1 should not be used when $O^2$ is supplied nasally.<br><br>Check patient has been entered into correct group. |
| Diet | Patient follows a dietary pattern? | | |
| SLT Management | SLT and swallow assessment<br>Further SLT management<br>Other electrical stimulation treatments | Current management | |
| Medications | List of dysphagia medications | | |
| Physiological assessment | Blood pressure / Respiration rate / Body temperature | | |
| Location within hospital | ICU (High dependency/general) / Stroke unit / Trauma unit / Neurology unit / ENT Unit | | |

[36] PAS = Penetration-Aspiration Scale.
[37] DHI = Dysphagia Handicap Index.
[38] FOIS = Functional Oral Intake Scale.
[39] SSS = Secretion Severity Scale.
[40] EQ5D = Quality of Life (5 Levels).
[41] EAT-10 = Eating Assessment Tool.
[42] NG = Nasogastric tube.
[43] PEG = Percutaneous Endoscopic Gastrostomy.
[44] RIG = Radiologically Inserted Gastrostomy.

### *c. CRF 04 - Treatment (PES) Form*

| Category | Tier one - Mandatory | Tier two – Optional | Query trigger |
|---|---|---|---|
| Patient Identification and Demographics | Patient ID / Date of assessment / Weight | | |
| Day 1 | Rx set-up:<br>Adverse event?<br>Catheter ID<br>Catheter used for feeding?<br>Location within hospital (ICU (High dependency/general) / Stroke unit / Trauma unit / Neurology unit / ENT Unit)<br>Catheter positioning:<br>Start time<br>Duration of insertion procedure<br>Ease of insertion-VAS<br>Depth marker?<br>Electrode position verification?<br>Adverse event?<br>Repositioning required?<br>More than one catheter used?<br>Rx Calibration:<br>Date<br>Start time<br>Threshold values (1, 2, 3,)<br>Average Threshold<br>Tolerance values (1, 2, 3)<br>Average Tolerance<br>Optimised level<br>Test Cycle performed?<br>Rx:<br>Intensity delivered?<br>Start time<br>Full treatment?<br>Post Rx assessment:<br>Catheter removed?<br>Adverse event?<br>Tolerance appreciation – by treater and patient? - VAS | | |
| Day 2 | Date / start time of Rx<br>Catheter positioning:<br>New catheter required?<br>More than one catheter used?<br>Start time<br>Duration of insertion procedure | | If time of day differs with > 2 hrs from day 1<br><br>If day 2 is not completed within 24 |

| | | | |
|---|---|---|---|
| | Ease of insertion-VAS<br>Repositioning required?<br>Adverse event?<br>Depth marker?<br>Rx Calibration:<br>Start time<br>Threshold values (1, 2, 3,)<br>Average Threshold<br>Tolerance values (1, 2, 3)<br>Average Tolerance<br>Optimised level<br>Test Cycle performed?<br>Rx:<br>Intensity delivered?<br>Start time<br>Full treatment?<br>Post Rx assessment:<br>Catheter removed?<br>Adverse event?<br>Tolerance appreciation – by treater and patient? - VAS | | hrs of day 1 → automatic email to sponsor |
| Day 3 | Date / start time of Rx<br>Catheter positioning:<br>New catheter required?<br>More than one catheter used?<br>Start time<br>Duration of insertion procedure<br>Ease of insertion-VAS<br>Repositioning required?<br>Adverse event?<br>Depth marker?<br>Rx Calibration:<br>Start time<br>Threshold values (1, 2, 3,)<br>Average Threshold<br>Tolerance values (1, 2, 3)<br>Average Tolerance<br>Optimised level<br>Test Cycle performed?<br>Rx:<br>Intensity delivered?<br>Start time<br>Full treatment?<br>Post Rx assessment:<br>Catheter removed?<br>Adverse event?<br>Tolerance appreciation – by treater and patient? - VAS | | If time of day differs with > 2 hrs from day 1<br><br>Provide access to "Additional Rx form" |

| | | | |
|---|---|---|---|
| Day 4 and subsequent Rx's | Similar to Day 3 but only when applied<br>Material used?<br>Catheter ID | Why? | Warning Off Label use |

### *d. CRF 05 - Follow-up form (General form for all follow-up visits)*

| Category | Tier one – Mandatory | Tier two - Optional | Query trigger |
|---|---|---|---|
| Patient Identification and Demographics | Patient ID<br>Date of current follow-up:<br>- 24-72hrs<br>- Unscheduled (72hrs – 7 days)<br>- 7 days – discharge<br>- Unscheduled (pre-discharge up to 60 days)<br>- 60-120 days<br>Weight / Fat percentage<br>Adverse event? | | |
| Location within hospital | ICU (High dependency/general) / Stroke unit / Trauma unit / Neurology unit / ENT Unit<br>Discharge destination / date / destination / contact details | | |
| Dysphagia Severity | Diagnostic method (VFS / FEES / Bedside / DSRS / other?)<br>Signs and symptoms<br>PAS-score<br>DSRS-score | | Warning if FOIS or DSRS score are not completed. |
| Questionnaires | DHI / FOIS / SSS / EQ-5D / EAT-10 | Additional disease specific questionnaires | |
| Feeding status | Eating Orally +/_ supervision?<br>Duration of feeding session<br>Nasogastric tube (NG)<br>PEG/RIG<br>Awaiting PEG/RIG/NG<br>Awaiting surgical intervention | | |
| Ventilation status | Patient self-breathing?<br>Nasal Oxygen support? / Date of instalment?<br>Ventilation support? / Via? / Date of instalment?<br>Tracheotomy for dysphagia only?<br>Previously ventilated? / Start date/ Date weaning completed? | | Warning: EPS1 should not be used when $O^2$ is supplied nasally.<br><br>Check patient has been entered into correct group. |
| Decannulation efforts | Patient still requires ventilation?<br>Date of weaning<br>Duration of cannulation | | |

| | | | |
|---|---|---|---|
| | Decannulation protocol/assessment used? | | |
| Feeding / Ventilation interaction | Ventilation prevents oral feeding?<br>Feeding route (NG / PEG/Pyoenteral) | | |
| Diet | Patient follows a dietary pattern? | | |
| SLT Management | SLT and swallow assessment<br>Further SLT management<br>Other electrical stimulation treatments | Current management | |
| Medications | List of dysphagia medications | | |
| Physiological assessment | Blood pressure / Respiration rate / Body temperature | | |
| General physical condition | Level of disability in arms / legs / capacity to walk / requirement of aids | Comorbidities<br>Current presentation (disease specific) | |

***e. CRF 06 - Adverse Event Form***

| **Category** | **Tier one – Mandatory** | **Tier two - Optional** | **Query trigger** |
|---|---|---|---|
| Patient Identification | Patient ID<br>Date of reporting Adverse event? | | |
| Report Status | Initial / Additional / Final | | |
| Adverse event | Date of observation / onset<br>Description<br>Classification and Relationship to device<br>Remedial treatment<br>Outcome | | |

***f. CRF 07 - Site information form (Tier 1)***

| **Category** | **Tier one - Mandatory** | **Tier two - Optional** | **Query trigger** |
|---|---|---|---|
| Investigator | Name<br>Contact details | | |
| Co-investigator | Name<br>Contact details | | |
| SLT | Name<br>Contact details | | |
| Co-SLT | Name<br>Contact details | | |
| Clinical coordinator | Name<br>Contact details | | |
| Other | Name<br>Contact details | | |
| Pre-study Appreciation of Dysphagia | Diagnostic method:<br>VFS / FEES / Bedside / | | Method should remain similar throughout the |

| | | | |
|---|---|---|---|
| assessments and Rx<br><br><br><br><br>Investigator<br><br>SLT | DSRS / other?) /<br>Number of PES Rx<br>already delivered<br><br>Rx appreciation / Selection decisions<br><br>SLT-Rx: VAS<br>E-Stim: VAS<br>SLT-Rx: VAS<br>E-Stim: VAS | | study. |
| Reimbursement for dysphagia Rx | Hospital own budget?<br>Department budget?<br>Health Insurance? | | |
| Reimbursement for PES Rx | Hospital own budget?<br>Department budget?<br>Health Insurance? | | |
| Ease of Use | Rx / IFU / Catheter | | |
| Pre-study Appreciation of Dysphagia assessments and Rx<br><br><br><br><br><br>Investigator<br><br>SLT | VFS / FEES / Bedside / DSRS / other? / Number of PES Rx already delivered<br><br>Rx appreciation / Selection decisions<br><br>SLT-Rx: VAS<br>E-Stim: VAS<br>SLT-Rx: VAS<br>E-Stim: VAS | | During conduct of study, data of pre-assessment are not visible till after closure of study to avoid bias. |

***g. CRF 08 - Lost-to-FU & withdrawal from study form***

| **Category** | **Tier one – Mandatory** | **Tier two - Optional** | **Query trigger** |
|---|---|---|---|
| LTFU / Withdrawal | Patient ID:<br>Attempts made to FU<br>Outcome of FU<br>Withdrawal of consent<br>Date of last FU<br>Date of exit | | |

## A.7: Patient Data Collection Flow Chart

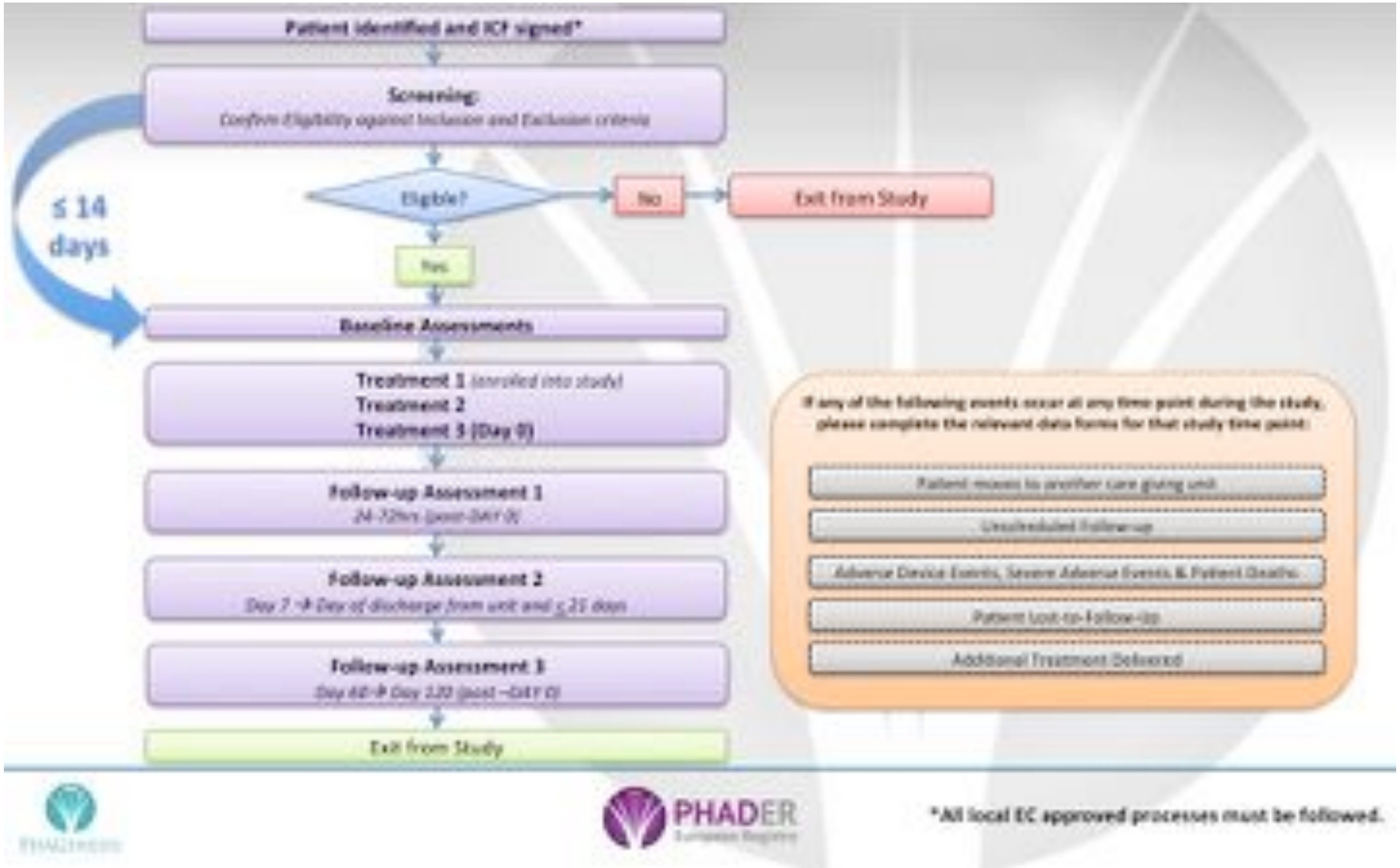

## A.8: Patient Card

### PHADER

**Prospective single arm observational post-market follow-up study on PHAryngeal electrical stimulation to treat neurogenic Dysphagia: a European Registry.**

**The patient carrying this card participates in the above listed study. At the time of registration the patient confirmed his/her voluntary participation in the study and has not withdrawn since. This study involves a pharyngeal electrical stimulation (PES) treatment to improve the swallowing function. Changes in swallowing function are observed in the study over a period of 3 months, thereafter the patient exits from the study.**

**Please help assure the appropriate clinical data are continuously recorded. To know the study requirements please contact the investigator:**

**Name investigator: ___________________________**

**Contact details: ______________________________**

**Registration date in the study: _________________ Exit date for the patient: ___________**

## A.9: Speech and Language Therapy Management

N.B: this assessment must be consistently done at all time points by the same person for this patient.
Patient's Speech and Language Therapist's Name:……………………………………………………………….

Date of swallow assessment: (dd/mmm/yyyy) ……………………………………… _ _ / _ _ _ / _ _ _ _

Indicate on the following scale the severity of swallowing as assessed by the SLT:
Where 1 = no problem at all to swallow fluids or any food;
Where 7 = unable to swallow and requires instrumentation.

| | | | | | | | | |
|---|---|---|---|---|---|---|---|---|
| - Retrospective assessment at the time of start of SLT-management (this hospitalisation period): | 1 | 2 | 3 | 4 | 5 | 6 | 7 | N/A |
| - Current assessment at the time of baseline: | 1 | 2 | 3 | 4 | 5 | 6 | 7 | N/A |

**Current Management** (tick all options that apply):

**YES**

1. The current SLT Management plan is based on information from:
   a. SLT led bedside swallow assessment? ……………………………………………………… ☐
   b. Clinical videofluoroscopy (i.e. local SLT practitioner protocol or radiology led; VFS)? ……………………………………………………………………………………………… ☐
   c. Fibreoptic Endoscopic Evaluation of Swallowing (FEES)? …………………………… ☐
   d. Other (Please specify): ……………………………………………………………………… ☐
   ……………………………………………………………………………………………………………
2. Diet recommendations (tick as appropriate):
   a. None? ……………………………………………………………………………………………… ☐
   b. Normal? …………………………………………………………………………………………… ☐
   c. Normal avoiding challenging textures? ……………………………………………… ☐
   d. Soft? ………………………………………………………………………………………………… ☐
   e. Soft-mashed? …………………………………………………………………………………… ☐
   f. Puree? ……………………………………………………………………………………………… ☐
   g. Nil oral diet? …………………………………………………………………………………… ☐
   h. Other (e.g. puree trials, please specify): …………………………………………………
   ……………………………………………………………………………………………………………
3. Current fluid recommendations (tick as appropriate):
   a. None? ……………………………………………………………………………………………… ☐
   b. Normal? …………………………………………………………………………………………… ☐
   c. Syrup? ……………………………………………………………………………………………… ☐
   d. Custard? …………………………………………………………………………………………… ☐
   e. Pudding? …………………………………………………………………………………………… ☐
   f. Nil oral fluids?…………………………………………………………………………………… ☐
   g. Other (Please specify): ………………………………………………………………………
   ……………………………………………………………………………………………………………

4. Specific instrumentation that have currently been installed to manage the patients dysphagia: (tick as appropriate):
   a. None, no supplementary or alternative feeding is being given? ………………… ☐
   b. PEG? ……………………………………………………………………………………………….. ☐
   c. Nasogastric (NG) tube? ……………………………………………………………………… ☐
   d. Other (Please specify): ……………………………………………………………………….
   ……………………………………………………………………………………………………………
5. Additional advice given to the patient in relation to feeding: (tick as appropriate):
   a. None? ……………………………………………………………………………………………… ☐
   b. Assistance to feed? ………………………………………………………………………… ☐
   c. Supervision & prompts re: bolus size or speed? ………………………………………… ☐
   d. Restrictions in quantity of oral intake per sitting? …………………………………….. ☐
   e. Altered frequency of oral intake? ……………………………………………………………… ☐
   f. Specified body position during oral intake? ……………………………………………….. ☐
   g. Use of specialist equipment or utensils? …………………………………………………… ☐
   h. Specified regime or oral hygiene? …………………………………………………………… ☐
   i. Specified patient/care education? ……………………………………………………………. ☐
   j. Other (Please specify): ……………………………………………………………………….
   ……………………………………………………………………………………………………………
6. Use of any compensatory strategies: (tick as appropriate):
   a. None? ……………………………………………………………………………………………….. ☐
   b. Chin tuck? ………………………………………………………………………………………….. ☐
   c. Head turn? …………………………………………………………………………………………. ☐
   d. Supraglottic swallow? …………………………………………………………………………… ☐
   e. Effortful swallow? ……………………………………………………………………………….. ☐
   f. Other (Please specify): ……………………………………………………………………….
   ……………………………………………………………………………………………………………
7. Current dysphagia therapy being applied: (tick as appropriate):
   a. None? ………………………………………………………………………………………………. ☐
   b. Oromotor exercises? …………………………………………………………………………… ☐
      IF applied: Number of sessions a day? ………………………………………………… __
      Only when SLT is present? ……………………………………………………………………. ☐
   c. SLT led rehabilitative exercise (e.g. Shaker, Mendelsohn)? ……………………….. ☐
   d. Biofeedback led rehabilitative exercise (e.g. EMG)? ………………………………… ☐
   e. Thermal / tactile stimulation? ………………………………………………………………… ☐
   f. Other (Please specify): ……………………………………………………………………….
   ……………………………………………………………………………………………………………
8. Identify any other SLT swallow related management input:
   ……………………………………………………………………………………………………………………
   ……………………………………………………………………………………………………………………

**Further management** (answer all questions):

| | YES | NO | UNK |
|---|---|---|---|
| 1. Was an SLT management plan set-up since the dysphagia causing event? ………… | ☐ | ☐ | ☐ |
| If YES, | | | |
| a. When was it started up? (dd/mmm/yyyy) ………… __ / ___ / ____ | | | |
| 2. Was the SLT management plan considered successful? ………… | ☐ | ☐ | ☐ |
| If YES, | | | |
| a. Did swallowing ability improve considerably? ………… | ☐ | ☐ | ☐ |
| b. Was diet adjusted towards a more normal diet? ………… | ☐ | ☐ | ☐ |
| c. Was fluid intake adjusted towards a more regular pattern? ………… | ☐ | ☐ | ☐ |
| 3. Was the SLT management plan considered completed with no further follow-up treatments scheduled? ………… | ☐ | ☐ | ☐ |

**Other dysphagia related treatments:**

| | YES | NO |
|---|---|---|
| Is there any plan at the time of baseline assessment to administer other dysphagia treatments to the patient in the near future? ………… | ☐ | ☐ |
| IF YES, please indicate if any of the following are to be applied: | | |
| a. Transcranial Direct Current Stimulation? ………… | ☐ | ☐ |
| b. Transcranial Electrical Stimulation? ………… | ☐ | ☐ |
| c. Transcutaneous Neuromuscular Electrical Stimulation? ………… | ☐ | ☐ |
| d. Pharyngeal Electrical Stimulation? ………… | ☐ | ☐ |
| e. Other? (Please specify) ………… | | |

## B.1: Dysphagia Severity Rating Scale (DSRS)

A score from 0 to 4 is given for three different elements (fluids, diet, and supervision):

FLUIDS:

0. Normal fluids
1. Syrup consistency
2. Custard consistency
3. Pudding consistency
4. No oral fluids

DIET:

0. Normal
1. Selected textures
2. Soft, moist diet
3. Puree
4. Non-oral feeding

SUPERVISION

0. Eating orally completely independently
1. Eating with supervision
2. Feeding by third party (untrained)
3. Therapeutic feeding (SLT / trained staff)
4. No oral feeding

Note: A score of 4 or higher is considered unsafe; a score between 9 and 12 is considered severe.

## B.2: Functional Oral Intake Scale (FOIS)[45]

TUBE DEPENDENT (levels 1-3)

1 No oral intake

2 Tube dependent with minimal/inconsistent oral intake

3 Tube supplements with consistent oral intake

TOTAL ORAL INTAKE (levels 4-7)

4 Total oral intake of a single consistency

5 Total oral intake of multiple consistencies requiring special preparation

6 Total oral intake with no special preparation, but must avoid specific foods or liquid items

7 Total oral intake with no restrictions

[45] Crary MA, Carnaby-Mann GD, Groher ME. Initial psychometric assessment of a functional oral intake scale for dysphagia in stroke patients. Arch Phys Med Rehabil 2005;86:1516-1520.

## B.3: Eating Assessment Tool (EAT-10)

A score from 0 to 4 is given on each of the following 10 questions, according to the following severity scale:

0 = no problem

4 = severe problem

A total score is calculated by making the sum of the values collected.

QUESTIONS: To what extent do you experience the following problem:

1. My swallowing problem has caused me to lose weight
2. My swallowing problem interferes with my ability to go out for meals
3. Swallowing liquids takes extra effort
4. Swallowing solids takes extra effort
5. Swallowing pills takes extra effort
6. Swallowing is painful
7. The pleasure of eating is affected by my swallowing
8. When I swallow food sticks in my throat
9. I cough when I eat
10. Swallowing is stressful

## B.4: Penetration and Aspiration Scale (PAS)

A score from 1-8 is obtained according to the following scale:

1. Material does not enter the airway
2. Material enters the airway, but remains above vocal cords & is ejected from airway
3. Material enters the airway, but remains above vocal cords & is NOT ejected from airway
4. Material enters the airway, contacts vocal cords & is ejected from airway
5. Material enters the airway, contacts vocal cords & is NOT ejected from airway
6. Material enters the airway, passes below vocal cords & is ejected into the larynx or out of the airway
7. Material enters the airway, passes below vocal cords & is NOT ejected from the trachea despite effort
8. Material enters the airway, passes below vocal cords & no effort is made to eject the material

## B.5: Secretion Severity Rating Scale (SSS)[46]

Based on a laryngoscopic or FEES evaluation of swallowing the SSS-score can be delineated:

| | | |
|---|---|---|
| 0 | Normal rating | Ranges from no visible secretions anywhere in the hypopharynx, to some transient secretions visible in the valleculae and pyriform sinuses. These secretions are not bilateral or deeply pooled. |
| 1 | Mild rating | Any secretion evident upon entry or following a dry swallow in the protective structures surrounding the laryngeal vestibule that are bilaterally represented or deeply pooled. This rating would include cases in which there is a transition in the accumulation of secretions during observation segment. |
| 2 | Moderate rating | Any secretion that change from “1” rating to a “3” rating during the observation period. |
| 3 | Most severe rating | Any secretion seen in the area defined as laryngeal vestibule. Pulmonary secretions are included if they are not cleared by swallowing or coughing by the close of the segment. |

[46] Murray et al. 1996: "The significance of accumulated oropharyngeal secretions and swallowing frequency in predicting aspiration. Dysphagia 11: 99-103.

## B.6: Barthel Index (BI)

**THE BARTHEL INDEX**

**Patient Name:** ____________________

**Rater Name:** ____________________

**Date:** ____________________

| Activity | Score |
|---|---|
| **FEEDING**<br>0 = unable<br>5 = needs help cutting, spreading butter, etc., or requires modified diet<br>10 = independent | ____ |
| **BATHING**<br>0 = dependent<br>5 = independent (or in shower) | ____ |
| **GROOMING**<br>0 = needs to help with personal care<br>5 = independent face/hair/teeth/shaving (implements provided) | |
| **DRESSING**<br>0 = dependent<br>5 = needs help but can do about half unaided<br>10 = independent (including buttons, zips, laces, etc.) | ____ |
| **BOWELS**<br>0 = incontinent (or needs to be given enemas)<br>5 = occasional accident<br>10 = continent | ____ |
| **BLADDER**<br>0 = incontinent, or catheterized and unable to manage alone<br>5 = occasional accident<br>10 = continent | ____ |
| **TOILET USE**<br>0 = dependent<br>5 = needs some help, but can do something alone<br>10 = independent (on and off, dressing, wiping) | ____ |
| **TRANSFERS (BED TO CHAIR AND BACK)**<br>0 = unable, no sitting balance<br>5 = major help (one or two people, physical), can sit<br>10 = minor help (verbal or physical)<br>15 = independent | ____ |
| **MOBILITY (ON LEVEL SURFACES)**<br>0 = immobile or < 50 yards<br>5 = wheelchair independent, including corners, > 50 yards<br>10 = walks with help of one person (verbal or physical) > 50 yards<br>15 = independent (but may use any aid; for example, stick) > 50 yards | ____ |
| **STAIRS**<br>0 = unable<br>5 = needs help (verbal, physical, carrying aid)<br>10 = independent | ____ |

**TOTAL (0–100):** ____

As mentioned in the section of Analysis of Primary and Secondary Objectives, a score of -5 might be given to a patient who died during the study.

**The Barthel Activities of Daily Living Index Guidelines**

1. The index should be used as a record of what a patient does, not as a record of what a patient could do.

2. The main aim is to establish degree of independence from any help, physical or verbal, however minor and for whatever reason.

3. The need for supervision renders the patient not independent.

4. A patient's performance should be established using the best available evidence. Asking the patient, friends/relatives and nurses are the usual sources, but direct observation and common sense are also important. However direct testing is not needed.

5. Usually the patient's performance over the preceding 24-48 hours is important, but occasionally longer periods will be relevant.

6. Middle categories imply that the patient supplies over 50 per cent of the effort.

7. Use of aids to be independent is allowed.

**References**

Mahoney FI, Barthel D. "Functional evaluation: the Barthel Index." Maryland State Medical Journal 1965;14:56-61. Used with permission.

Loewen SC, Anderson BA. "Predictors of stroke outcome using objective measurement scales." Stroke. 1990;21:78-81.

Gresham GE, Phillips TF, Labi ML. "ADL status in stroke: relative merits of three standard indexes." Arch Phys Med Rehabil. 1980;61:355-358.

Collin C, Wade DT, Davies S, Horne V. "The Barthel ADL Index: a reliability study." Int Disability Study.1988;10:61-63.

## B.7: Euro-Qol-5D (5 level answers)

Under each heading, please tick the ONE box that best describes your health TODAY.

**MOBILITY**

I have no problems in walking about ❑
I have slight problems in walking about ❑
I have moderate problems in walking about ❑
I have severe problems in walking about ❑
I am unable to walk about ❑

**SELF-CARE**

I have no problems washing or dressing myself ❑
I have slight problems washing or dressing myself ❑
I have moderate problems washing or dressing myself ❑
I have severe problems washing or dressing myself ❑
I am unable to wash or dress myself ❑

**USUAL ACTIVITIES** *(e.g. work, study, housework, family or leisure activities)*

I have no problems doing my usual activities ❑
I have slight problems doing my usual activities ❑
I have moderate problems doing my usual activities ❑
I have severe problems doing my usual activities ❑
I am unable to do my usual activities ❑

**PAIN / DISCOMFORT**

I have no pain or discomfort ❑
I have slight pain or discomfort ❑
I have moderate pain or discomfort ❑
I have severe pain or discomfort ❑
I have extreme pain or discomfort ❑

**ANXIETY / DEPRESSION**

I am not anxious or depressed ❑
I am slightly anxious or depressed ❑
I am moderately anxious or depressed ❑
I am severely anxious or depressed ❑
I am extremely anxious or depressed ❑

- We would like to know how good or bad your health is TODAY.
- This scale is numbered from 0 to 100.
- 100 means the <u>best</u> health you can imagine.
  0 means the <u>worst</u> health you can imagine.
- Mark an X on the scale to indicate how your health is TODAY.
- Now, please write the number you marked on the scale in the box below.

YOUR HEALTH TODAY = ☐

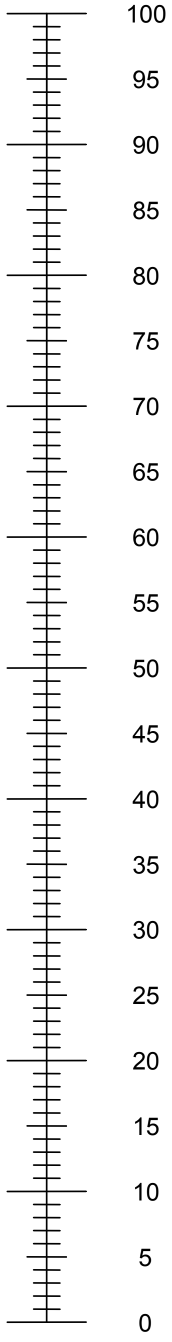

## B.8: Dysphagia Handicap Index (DHI)[47]

| Question | Subscale | Never (score=0) | Sometimes (score=2) | Always (score=4) |
|---|---|---|---|---|
| I cough when I drink liquids | P | | | |
| I cough when I eat solid food | P | | | |
| My mouth is dry | P | | | |
| I need to drink fluids to wash food down | P | | | |
| I've lost weight because of my swallowing problem | P | | | |
| I avoid some foods because of my swallowing problem | F | | | |
| I have changed the way I swallow to make it easier to eat | F | | | |
| I'm embarrassed to eat in public | E | | | |
| It take me longer to eat a meal than it used to | F | | | |
| I eat smaller meals more often due to my swallowing problem | F | | | |
| I have to swallow again before food will go down | P | | | |
| I feel depressed because I can't eat what I want | E | | | |
| I don't enjoy eating as much as I used to | E | | | |
| I don't socialize as much due to my swallowing problem | F | | | |
| I avoid eating because of my swallowing problem | F | | | |
| I eat less because of my swallowing problem | F | | | |
| I am nervous because of my swallowing problem | E | | | |
| I feel handicapped because of my swallowing problem | E | | | |
| I get angry at myself because of y swallowing problem | E | | | |
| I choke when I take medication | P | | | |
| I'm afraid that I'll choke and stop breathing because of my swallowing problem | E | | | |

[47] The DHI is currently under validation for the German translation version. See ClinicalTrial.gov, ref. NCT01958268

| | | | | |
|---|---|---|---|---|
| I must eat another way (e.g. feeding tube) because of my swallowing problem | F | | | |
| I've changed my diet due to my swallowing problem | F | | | |
| I feel a strangling sensation when I swallow | P | | | |
| I cough up food after I swallow | P | | | |

P = Physical, E = Emotional and F = Functional subscale. The sum of all scores is made for a given subscale and for the total DHI.

In addition, please indicate on the scale below by putting a circle around a number that matches the severity of your swallowing difficulty (1= no difficulty at all, 4= somewhat of a problem, 7 = the worse problem you could have).

1 2 3 4 5 6 7

Normal Moderate problem Severe Problem

## B.9: Modified Ranking Scale

(From'Van'Swieten'*et.al.* '1998)'

| Grade | Description |
|---|---|
| 0 | No symptoms at all |
| 1 | No significant disability despite symptoms: able to carry out all usual duties and activities |
| 2 | Slight disability: unable to carry out all previous activities but able to look after own affairs without assistance |
| 3 | Moderate disability: requiring some help, but able to walk without assistance |
| 4 | Moderately severe disability: unable to walk without assistance and unable to attend to own bodily needs without assistance |
| 5 | Severe disability: bedridden, incontinent and requiring constant nursing care and attention |
| 6 | Dead |

## B.10: National Institute Of Health Stroke Scale (NIHSS)

The National Institutes of Health Stroke Scale, or NIH Stroke Scale (NIHSS) is a tool used by healthcare providers to objectively quantify the impairment caused by a stroke. The NIHSS is composed of 11 items, each of which scores a specific ability between a 0 and 4. For each item, a score of 0 typically indicates normal function in that specific ability, while a higher score is indicative of some level of impairment. The individual scores from each item are summed in order to calculate a patient's total NIHSS score. The maximum possible score is 42, with the minimum score being a 0.

| Score | Stroke Severity |
|---|---|
| 0 | No Stroke Symptoms |
| 1-4 | Minor Stroke |
| 5-15 | Moderate Stroke |
| 16-20 | Moderate to Severe Stroke |
| 21-42 | Severe Stroke |

### *1. Level of Consciousness (LOC)*

Level of consciousness testing is divided into three sections. The first LOC items test for the patient's responsiveness. The second LOC item is based on the patient's ability to answer questions that are verbally presented by the examiner. The final LOC sub-section is based on the patient's ability to follow verbal commands to perform simple task. Although this item is broken into three parts, each sub-section is added to the final score as if it is its own item.

#### *A) LOC Responsiveness*

Scores for this item are assigned by a medical practitioner based on the stimuli required to arouse patient. The examiner should first assess if the patient is fully alert to his or her surroundings. If the patient is not completely alert, the examiner should attempt a verbal stimulus to arouse the patient. Failure of verbal stimuli indicates an attempt to arouse the patient via repeated physical stimuli. If none of these stimuli are successful in eliciting a response, the patient can be considered totally unresponsive.

| Score | Test Results |
|---|---|
| 0 | Alert; Responsive |
| 1 | Not alert; Verbally arousable or aroused by minor stimulation to obey, answer, or respond. |
| 2 | Not alert; Only responsive to repeated or strong and painful stimuli |
| 3 | Totally unresponsive; Responds only with reflexes or is a-reflexic |

***B) LOC Question***

Patient is verbally asked his or her age and for the name of the current month.

| Score | Test Results |
|---|---|
| 0 | Correctly answers both questions |
| 1 | Correctly answers one question |
| 2 | Does not correctly answer either question |

***C) LOC Commands***

The patient is instructed to first open and close his or her eyes and then grip and release his or her hand

| Score | Test Results |
|---|---|
| 0 | Correctly performs both tasks |
| 1 | Correctly performs 1 task |
| 2 | Does not correctly perform either task |

***2. Horizontal Eye Movement***

Assesses ability for patient to track a pen or finger from side to side only using his or her eyes. This is designed to assess motor ability to gaze towards the hemisphere opposite of injury. This item is tested because Conjugated eye deviation is present in approximately 20% of stroke cases. CED is more commons in right hemispheric strokes and typically in lesions effecting the basal ganglia and temporoparietal cortex. Damage to these areas can result in decreased spatial attention and reduced control of eye movements.

| Score | Test Results |
|---|---|
| 0 | Normal; Able to follow pen or finger to both sides |
| 1 | Partial gaze palsy; gaze is abnormal in one or both eyes, but gaze is not totally paralyzed. Patient can gaze towards hemisphere of infarct, but can't go past midline |
| 2 | Total gaze paresis; gaze is fixed to one side |

***3. Visual field test***

Assess the patient's vision in each visual fields. Each eye is tested individually, by covering one eye and then the other. Each upper and lower quadrant is tested by asking the patient to indicate how many fingers the investigator is presenting in each quadrant. The investigator should instruct the patient to maintain eye contact throughout this test, and not allow the patient to realign focus towards each stimulus. With the first eye covered, place a random number of fingers in each quadrant and ask the patient how many fingers are being presented. Repeat this testing for the opposite eye.

| Score | Test Results |
|---|---|
| 0 | No vision loss |
| 1 | Partial hemianopia or complete quadrantanopia; patient recognizes no visual stimulus in one specific quadrant |
| 2 | Complete hemianopia; patient recognizes no visual stimulus in one half of the visual field |
| 3 | Bilateral Blindness, including blindness from any cause |

***4. Facial Palsy***

Facial palsy is partial or complete paralysis of portions of the face. Typically this paralysis is most pronounced in the lower half of one facial side. However, depending on lesion location the paralysis may be present in other facial regions. While inspecting the symmetry of each facial expression the examiner should first instruct patient to show his or her teeth (or gums). Second, the patient should be asked to squeeze his or her eyes closed as hard as possible. After reopening his or her eyes, the patient is then instructed to raise his or her eyebrows.

| Score | Test Results |
|---|---|
| 0 | Normal and symmetrical movement |
| 1 | Minor paralysis; function is less than clearly normal, such as flattened nasolabial fold or minor asymmetry in smile |
| 2 | Partial paralysis; particularly paralysis in lower face |
| 3 | Complete facial Hemiparesis, total paralysis in upper and lower portions of one face side |

***5. Motor Arm***

With palm facing downwards, have the patient extend one arm 90 degrees out in front if the patient is sitting, and 45 degrees out in front if the patient is lying down. If necessary, help the patient get into the correct position. As soon as the patient's arm is in position the investigator should begin verbally counting down from 10 while simultaneously counting down on his or her fingers in full view of the patient. Observe to detect any downward arm drift prior to the end of the 10 seconds. Downward movement that occurs directly after the investigator places the patient's arm in position should not be considered downward drift. Repeat this test for the opposite arm. This item should be scored for the right and left arm individually, denoted as item 5a and 5b.

| Score | Test Results |
|---|---|
| 0 | No arm drift; the arm remains in the initial position for the full 10 seconds |
| 1 | Drift; the arm drifts to an intermediate position prior to the end of the full 10 seconds, but not at any point relies on a support |
| 2 | Limited effort against gravity; the arm is able to obtain the starting position, but drifts down from the initial position to a physical support prior to the end of the 10 seconds |
| 3 | No effort against gravity; the arm falls immediately after being helped to the initial position, however the patient is able to move the arm in some form (e.g. shoulder shrug) |
| 4 | No movement; patient has no ability to enact voluntary movement in this arm |

***6. Motor Leg***

With the patient in the supine position, one leg is placed 30 degrees above horizontal. As soon as the patient's leg is in position the investigator should begin verbally counting down from 5 while simultaneously counting down on his or her fingers in full view of the patient. Observe any downward leg drift prior to the end of the 5 seconds. Downward movement that occurs directly after the investigator places the patient's leg in position should not be considered downward drift. Repeat this test for the opposite leg. Scores for this section should be recorded separately as 6a and 6b for the left and right legs respectively.

| Score | Test Results |
|---|---|
| 0 | No leg drift; the leg remains in the initial position for the full 5 seconds |
| 1 | Drift; the leg drifts to an intermediate position prior to the end of the full 5 seconds, but at no point touches the bed for support |
| 2 | Limited effort against gravity; the leg is able to obtain the starting position, but drifts down from the initial position to a physical support prior to the end of the 5 seconds |
| 3 | No effort against gravity; the leg falls immediately after being helped to the initial position, however the patient is able to move the leg in some form (e.g. hip flex) |
| 4 | No movement; patient has no ability to enact voluntary movement in this leg |

***7. Limb Ataxia***

This test for the presence of a unilateral cerebellar lesion, and distinguishes a difference between general weakness and incoordination. The patient should be instructed to first touch his or her finger to the examiner's finger then move that finger back to his or her nose, repeat this movement 3-4 times for each hand. Next the patient should be instructed to move his or her heel up and down the shin of his or her opposite leg. This test should be repeated for the other leg as well.

| Score | Test Results |
|---|---|
| 0 | Normal coordination; smooth and accurate movement |
| 1 | Ataxia present in 1 limb; rigid and inaccurate movement in one limb |
| 2 | Ataxia present in 2 or more limbs: rigid and inaccurate movement in both limbs on one side |

***8. Sensory***

Sensory testing is performed via pinpricks in the proximal portion of all four limbs. While applying pinpricks, the investigator should ask whether or not the patient feels the pricks, and if he or she feels the pricks differently on one side when compared to the other side.

| Score | Test Results |
|---|---|
| 0 | No evidence of sensory loss |
| 1 | Mild-to-Moderate sensory loss; patient feels the pinprick, however he or she feels as if it is duller on one side |
| 2 | Severe to total sensory loss on one side; patient is not aware he or she is being touched in all unilateral extremities |

***9. Language***

This item measures the patient's language skills. After completing items 1-8 it is likely the investigator has gained an approximation of the patient's language skills; however it is important to confirm this measurement at this time. The stroke scale includes a picture of a picture of a scenario, a list of simple sentences, a figure of assorted random objects, and a list of words. The patient should be asked to explain the scenario depicted in the first figure. Next, he or she should read the list of sentences and name each of the objects depicted in the next figure. The scoring for this item should be based on both the results from the test performed in this item in addition to the language skills demonstrated up to this point in the stroke scale.

| Score | Test Results |
|---|---|
| 0 | Normal; no obvious speech deficit |
| 1 | Mild-to-moderate aphasia; detectable loss in fluency, however, the examiner should still be able to extract information from patient's speech |
| 2 | Severe aphasia; all speech is fragmented, and examiner is unable to extract the figure's content from the patients speech. |
| 3 | Unable to speak or understand speech |

***10. Speech***

Dysarthria is the lack of motor skills required to produce understandable speech. Dysarthria is strictly a motor problem, and is not related to the patient's ability to comprehend speech. Strokes that cause dysarthria typically affect areas such as the anterior opercular, medial prefrontal and premotor, and anterior cingulate regions. These brain regions are vital in coordinating motor control of the tongue, throat, lips, and lungs. To perform this item the patient is be asked to read from the list of words provided with the stroke scale while the examiner observes the patients articulation and clarity of speech.

| Score | Test Results |
|---|---|
| 0 | Normal; clear and smooth speech |
| 1 | Mild-to-moderate dysarthria; some slurring of speech, however the patient can be understood |
| 2 | Severe dysarthria; speech is so slurred that he or she cannot be understood, or patients that cannot produce any speech |

***11. Extinction and Inattention***

Sufficient information regarding this item may have been obtained by the examiner in items 1-10 to properly score the patient. However, if any ambiguity exist the examiner should test this item via a technique referred to as "double simultaneous stimulation". This is performed by having the patient close his or her eyes and asking him or her to identify the side on which they are being touched by the examiner. During this time the examiner is alternating between touching the patient on the right and left side. Next, the examiner touches the patient on both sides at the same time. This should be repeated on the patients face, arms, and legs. To test extinction in vision, the examiner should hold up one finger in front of each of the patient's eyes and ask the patient to determine which finger is wiggling or if both are wiggling. The examiner should then alternate between wiggling each finger and wiggling both fingers at the same time.

| Score | Test Results |
|---|---|
| 0 | Normal; patient correctly answers all questions |
| 1 | Inattention on one side in one modality; visual, tactile, auditory, or spatial |
| 2 | Hemi-inattention; does not recognize stimuli in more than one modality on the same side |

## B.11: Glasgow Coma Scale (GCS)

The scale is composed of three tests: eye, verbal and motor responses. The three values separately as well as their sum are considered. The lowest possible GCS (the sum) is 3 (deep coma or death), while the highest is 15 (fully awake person).

| | 1 | 2 | 3 | 4 | 5 | 6 |
|---|---|---|---|---|---|---|
| Eye | Does not open eyes | Opens eyes in response to painful stimuli | Opens eyes in response to voice | Opens eyes spontaneously | N/A | |
| Verbal | Makes no sounds | Incomprehensible sounds | Utters inappropriate words | Confused, disoriented | Oriented, converses normally | N/A |
| Motor | Makes no movements | Extension to painful stimuli (decerebrate response) | Abnormal flexion to painful stimuli (decorticate response) | Flexion / Withdrawal to painful stimuli | Localizes painful stimuli | Obeys commands |

Individual elements as well as the sum of the score are important. Hence, the score is expressed in the form "GCS 9 = E2 V4 M3 at 07:35".

Generally, brain injury is classified as:

- Severe, with GCS < 8-9
- Moderate, GCS 8 or 9–12 (controversial)
- Minor, GCS ≥ 13.

Generally when a patient is in a decline of their GCS score, the nurse or medical staff should assess the cranial nerves and determine which of the twelve have been affected.

Tracheal intubation and severe facial/eye swelling or damage make it impossible to test the verbal and eye responses. In these circumstances, the score is given as 1 with a modifier attached e.g. "E1c" where "c" = closed, or "V1t" where t = tube. A composite might be "GCS 5tc". This would mean, for example, eyes closed because of swelling = 1, intubated = 1, leaving a motor score of 3 for "abnormal flexion". Often the 1 is left out, so the scale reads Ec or Vt.

## B.12: Fiberoptic Endoscopic Evaluation of Swallowing Protocol for Tracheostomy Decannulation

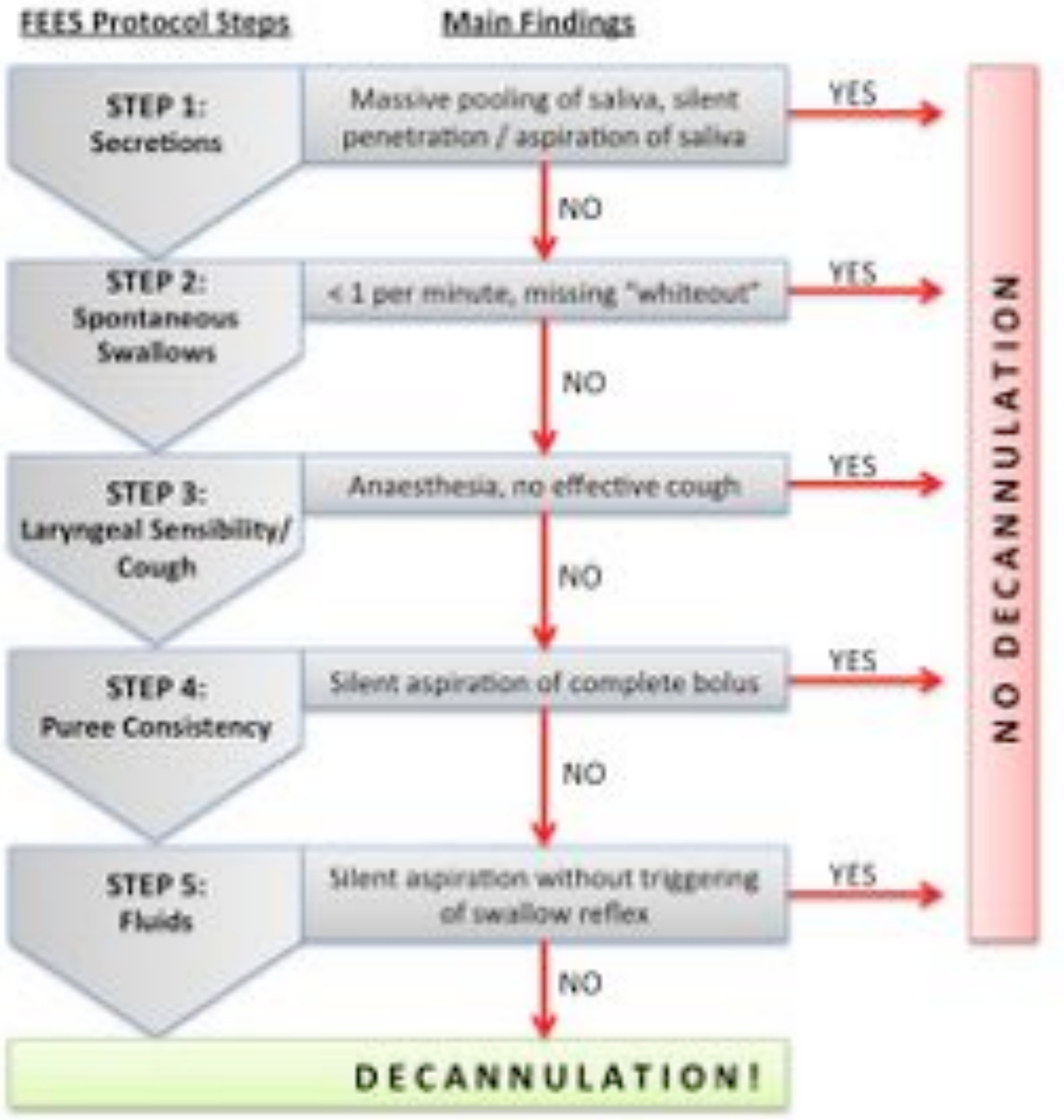

Supplement: Supplementary file 3 [file mmc3.pdf]
