# Supplementary material for: Pharyngeal electrical stimulation for neurogenic dysphagia following stroke, traumatic brain injury or other causes: Main results from the PHADER cohort study
Source: eClinicalMedicine. 2020 Nov 10;28:100608. doi: 10.1016/j.eclinm.2020.100608 (PMC7700977; doi:10.1016/j.eclinm.2020.100608)
Supplement: Supplementary file 4 [file mmc4.pdf]


STATISTICAL ANALYSIS PLAN
Protocol AHE-02
A prospective, single-arm, observational clinical follow-up study on the application of PHAryngeal electrical stimulation for treatment of neurogenic Dysphagia: A European Registry (PHADER)

**Protocol Number:**<br>**(Version Date)**	AHE-02<br>10 April 2015
**Name of Test Drug:**	CE-marked Phagenyx Base Station (EPS1) and Phagenyx Catheter
**Phase:**	4
**Methodology:**	Prospective, single-arm, observational registry
**Sponsor:**	Phagenesis Limited<br>Unit 18, Enterprise House, Pencroft Way<br>Manchester, United Kingdom. M15 6SE
**Sponsor Representative:**	Dr. Satish Mistry, Ph.D.<br>Clinical Project Manager
**Document Date:**	30 August 2019
**Document Version:**	1.0

Confidentiality

This document is confidential and proprietary property of Phagenesis Limited and to be used only as authorized by Phagenesis Limited. No part is to be reproduced, disclosed to others, or quoted without prior written authorization from Phagenesis Limited

SIGNATURE PAGE

Protocol Title: A prospective, single-arm, observational clinical follow-up study on the application of PHAryngeal electrical stimulation for treatment of neurogenic Dysphagia: A European Registry (PHADER)

Sponsor: Phagenesis Limited
Unit 18, Enterprise House, Pencroft Way
Manchester, United Kingdom. M15 6SE

Protocol Number: AHE-02

Document Date/Version: 30 August 2019/1.0

Cytel, Inc. Author:
Kelly P. Huang, MS
Cytel, Inc.
2200 Renaissance Blvd, Suite 370
King of Prussia, PA 19406

Signature: Kelly Huang

Electronically signed by: Kelly Huang
Reason: Approve
Date: 2019-08-30 09:44:06-04:00

Date: 30-Aug-2019

Sponsor Approval

By signing this document, I acknowledge that I have read the document and approve of the planned statistical analyses described herein. I agree that the planned statistical analyses are appropriate for this study, are in accordance with the study objectives, and are consistent with the statistical methodology described in the protocol, clinical development plan, and all applicable regulatory guidances and guidelines.

I have discussed any questions I have regarding the contents of this document with the biostatistical author.

I also understand that any subsequent changes to the planned statistical analyses, as described herein, may have a regulatory impact and/or result in timeline adjustments. All changes to the planned analyses will be described in the clinical study report (CSR).

Sponsor Signatory:
Dr. Satish Mistry, Ph.D.
Phagenesis Limited
Unit 18, Enterprise House, Pencroft Way
Manchester, United Kingdom. M15 6SE

Signature: Satish Mistry

Electronically signed by: Satish Mistry
Reason: Approve
Date: 2019-08-30 14:40:06+01:00

Date: 30-Aug-2019

TABLE OF CONTENTS

Section **Page**

1. INTRODUCTION AND OBJECTIVES OF ANALYSIS 7
1.1. Introduction 7
1.2. Objectives of Statistical Analysis 7
1.2.1. Primary Objective 7
1.2.2. Secondary Objectives 7
2. STUDY DESIGN 9
2.1. Synopsis of Study Design 9
2.2. Randomization Methodology 9
2.3. Study Procedures 9
2.4. Efficacy and Safety Variables 11
2.4.1. Efficacy Variables 11
2.4.2. Safety Variables 11
3. PATIENT POPULATIONS 12
3.1. Population Definitions 12
4. STATISTICAL METHODS 13
4.1. Sample Size Justification 13
4.2. General Statistical Methods and Data Handling 13
4.2.1. General Methods 13
4.2.2. Computing Environment 13
4.2.3. Withdrawals, Dropouts, Loss to Follow-up 13
4.2.4. Missing, Unused, and Spurious Data 14
4.3. Demographic and Baseline Characteristics 14
4.4. Efficacy Evaluation 14
4.4.1. PAS, DSRS, FOIS, Length of Stay in Hospital, and Death by Index Group 14
4.4.2. Effect of PES on Decannulation Status 15
4.4.3. User Experience of PES 16
4.4.4. PAS, DSRS, FOIS, Length of Stay in Hospital, and Death by Active and Sham Groups of PHAST-TRAC 16
4.4.5. Baseline Characteristics in Ventilated versus Non-ventilated Participants and Supratentorial/Anterior Circulation versus

Section **Page**

Infratentorial/Posterior Circulation Stroke Participants 16
4.4.6. Stimulation Levels 16
4.4.7. DSRS Subscales (Fluids, Diet, Supervision) by Index Group and Timing 16
4.4.8. PAS, DSRS, FOIS, Length of Stay in Hospital, and Death by Circulation Status (Supratentorial and Infratentorial) 16
4.4.9. PAS, DSRS, FOIS, Length of Stay in Hospital, and Death by Decannulation Status after PES 17
4.4.10. PAS, DSRS, FOIS, Length of Stay in Hospital, and Death by PHADER Stroke and STEPS sham Groups 17
4.5. Safety Analyses 17
4.5.1. Serious Adverse Events 17
5. CHANGES TO PLANNED ANALYSES 19
6. References 20
7. CLINICAL STUDY REPORT APPENDICES 21
7.1. Statistical Tables to be Generated 21
7.2. Figures to be Generated 22
7.3. Pseudo SAS Code 23
7.3.1. Fischer’s Exact Test 23
7.3.2. Chi-square Test 23
7.3.3. Kruskal-Wallis Test 23
7.3.4. Multiple Linear Regression 23
7.3.5. One-way ANOVA 24

LIST OF IN-TEXT TABLES

Table **Page**

Table 1 Schedule of Assessments 9

ABBREVIATIONS

Abbreviation	Definition
CIP	Clinical Investigation Plan
CSR	Clinical study report
DSRS	Dysphagia Severity Rating Scale
EAT-10	Eating Assessment Tool
FEES	Fiberoptic endoscopic evaluation of swallowing
FOIS	Functional Oral Intake Scale
GCS	Glasgow Coma Scale
mRS	Modified Rankin Scale
NIHSS	National Institutes of Health Stroke Severity
PAS	Penetration-Aspiration Scale
PES	Pharyngeal electrical stimulation
QoL	Quality of life
SAE	Serious adverse event
SAP	Statistical analysis plan
SLT	Speech and language therapist
SSS	Secretion Severity Scale
STEPS	A Study of Swallowing Treatment using Electrical Pharyngeal Stimulations
TBI	Traumatic brain injury
VFS	Videofluoroscopy

DOCUMENT HISTORY

Version Number	Author	Date	Change
1.0	Kelly Huang	30 August 2019	Issued

1. INTRODUCTION AND OBJECTIVES OF ANALYSIS

1.1. Introduction

Dysphagia might appear as the result of different indications and one can differentiate 3 groups:

1. Oropharyngeal dysphagia;
2. Oseophageal dysphagia;
3. Functional dysphagia without organic cause.

Stroke is known to cause the highest incidence of neurogenic dysphagia and was the subject of investigation in the Study of Swallowing Treatment using Electrical Pharyngeal Stimulations (STEPS) study. In stroke-patients, pharyngeal electrical stimulation (PES) at specific frequencies has been shown to enhance brain plasticity[1]. This concept led to the development of the (Phagenesis sponsored) STEPS study (AHE-01 – A multi-center, double-blind, randomized controlled Clinical Investigation to validate the EPS1 device as a treatment for stroke-induced dysphagia: A Study of Swallowing Treatment using Electrical Pharyngeal Stimulation).

As per the Medical Device Directive (93/42/EEC, amended by Directive 2007/47/EC) the manufacturer can perform post-market clinical follow-up studies to comply with the directive's product surveillance requirements (post-market surveillance). For this purpose, an observational study without any requirement to apply medical treatment beyond standard medical practice is judged appropriate. This study plans to obtain more evidence on the use and clinical outcome of the Phagenyx products when used to treat neurogenic dysphagia in daily practice (a 'real world' environment).

This statistical analysis plan (SAP) is designed to outline the methods to be used in the analysis of study data in order to answer the study objective(s). Populations for analysis, data handling rules, statistical methods, and formats for data presentation are provided. This SAP will also outline any differences in the currently planned analytical objectives relative to those planned in the study protocol.

1.2. Objectives of Statistical Analysis

1.2.1. Primary Objective

The primary objective of this observational registry is to demonstrate the 'real world' clinical outcomes of PES treatment for neurogenic dysphagia resulting from different causes post-PES, i.e. just prior to hospital discharge. The clinical outcome is defined by means of standard functional scoring systems: Dysphagia Severity Rating Scale (DSRS), Functional Oral Intake Scale (FOIS), and Penetration-Aspiration Scale (PAS)-scores.

1.2.2. Secondary Objectives

A number of secondary objectives listed in the Clinical Investigation Plan (CIP) will not be analyzed due to no data or insufficient data provided by participating centers.

- To obtain additional evidence for the continued product safety and performance profile;
- To verify the product's safety and performance profile when exposed to a larger and more varied population of clinical users than studies thus far;

- To describe the demographics, symptoms and underlying causes of neurogenic dysphagia in treated patients;
- To assess the severity of dysphagia by means of standard scoring systems (DSRS, FOIS, and PAS);
- To document the time point of PES therapy delivery in relation to the causal event;
- To measure threshold, tolerance, and treatment current of PES on different days of delivery
- To assess the effectiveness of PES at different time points during the day;
- To document the feeding status at baseline and post-PES at the time of pre-hospital discharge;
- To document the artificial ventilation status at baseline and post-PES at the time of pre-hospital discharge;
- To document the duration of cannulation (intubation/tracheotomy) and timing of decannulation with respect to the timing of PES-treatment;
- To document serious adverse events (SAEs) and device deficiencies during the observational study period;
- To document the health economic aspects of treatment by means of measurement of duration of hospital stay and duration of mechanical ventilation;
- To document the rehabilitation practices by means of defining the patient flow from one care given unit to another or to their own home over at time period of three months

2. STUDY DESIGN

2.1. Synopsis of Study Design

This post-market clinical follow-up study is a prospective, multi-center, European, observational single-arm, clinical investigation to evaluate the clinical outcome of PES delivered at a given time after a neurological event causing dysphagia by means of the Phagenyx treatment (10 minutes of stimulation at an intensity level of 75% of the difference between the upper tolerable and lower threshold intensity level and set above this lower threshold at treatment during three consecutive days). All elements of this clinical trial are observational, i.e. no specific medical interventions or clinical procedures are required to be applied – only "standard" or "routine" medical care will be applied, and available ('real world') clinical data are requested to be documented into the Registry for the purposes of post-market surveillance.

Patients meeting all of the study inclusion and who do not meet any of the exclusion criteria can be considered for study participation by physicians from selected investigational centers where the local Ethics Committee have approved the conduct of the study.

2.2. Randomization Methodology

Randomization will not be conducted for this single-arm study.

2.3. Study Procedures

The schedule of assessments, as outlined in the study protocol, is provided in Table 1~~Table 1~~.

Table 1 Schedule of Assessments

	**Screening**	**Baseline**	**Last day of PES Treatment**	**Follow Up-assessment 1**	**Follow Up-assessment 2**	**Follow Up-assessment 3**
Timing (Period of data collection)	*Any time*	*<14 days after screening & prior to first treatment*	*DAY 0*[1]	*>24 <72 hrs*	*Day 7 → day of discharge from unit and ≤21 days*	*Day 60 → day 120 days*
Informed Consent	X					
Inclusion/exclusion criteria assessment	X					
Demographic data		X				
Dysphagia causal event documentation (and medical history)		X				
Dysphagia severity assessment (DSRS)	**X**	(§)[2]		X	X	X
FOIS-score	**X**	(§)[2]		X	X	X
Dysphagia severity assessment (PAS)	**X**	(§)[2]		X	X	(X)[3]
Feeding status documentation		X	X	X	X	X
Ventilation and decannulation status documentation		X	X	X	X	X
Other functional assessments		X		X	X	X

Quality of Life assessments (EQ5D)		X		X	X	X
SLT-management		X	X	X	X	(X)[3]
PES treatment		(§)[2]	X			
Care giving unit arrival/ disposition				X	X	X
Adverse Device Events (ADE) Severe Adverse Events (SAE) & Patient Deaths		X	X	X	X	X

[1] 'DAY 0': last day of PES treatment delivered during the conduct of this investigation.

[2] (§): If time period between Screening and Baseline exceeds 14 days, dysphagia severity assessment should be repeated to re-establish dysphagia severity at baseline. Baseline and treatment day 1 can be the same day.

[3] (X): Indicates data collection only when patient is in the hospital.

2.4. Efficacy and Safety Variables

2.4.1. Efficacy Variables

A number of efficacy endpoints listed on the CIP will not be analyzed due to no data or insufficient data provided by participating centers.

The primary endpoints are comprised of two standard functional scoring systems and one instrumental scoring system. The scores will be documented after the causal event and within one-week prior-PES (baseline), between 24 and 72 hours post-PES, and at pre-hospital discharge:

- DSRS: Dysphagia Severity Rating Scale (a score of 0 to 4 is given for three different elements (fluids, diet, and supervision))
- FOIS: Functional Oral Intake Scale (a score of 0 to 7)
- PAS: Penetration Aspiration Scale (a score of 1 to 8)

For PAS-score determination, either videofluoroscopy (VFS) or fiberoptic endoscopic evaluation of swallowing (FEES) methods can be applied. The choice is left to the discretion of the physician and should be in accordance with local practice and regulations.

The following secondary endpoints will be evaluated at baseline, between 24 and 72 hours post-PES, at pre-hospital discharge, and at three months as applicable:

- Demographics (age and sex)
- Symptoms and underlying causes of dysphagia in treated patients
- Time period between PES therapy delivery and the dysphagia causal event (date of treatment on Day 1 – date of dysphagia causal event + 1)
- Treatment optimization parameters prior to every treatment delivery (sensory threshold, upper tolerance limit of the treatment current and the determination of the (75%) optimized treatment level)
- Feeding status by means of the FOIS-score and the PEG-status
- Type, purpose, and duration of artificial ventilation
- Timing of extubation and decannulation (in tracheotomy patients)
- Disease severity and functional ability using multiple questionnaires such as: modified Rankin Scale (mRS), National Institutes of Health Stroke Severity (NIHSS) scale, and Glasgow Coma Scale (GCS)
- Health economics by assessment of the duration of the Intensive Care Unit (ICU)-stay/hospitalization/stay at different care giving units/discharge to home and of the total duration of mechanical ventilation/cannulation for a given purpose

2.4.2. Safety Variables

Serious adverse events (SAEs) and device deficiencies that occur during the observational study period are secondary safety endpoints.

3. PATIENT POPULATIONS

3.1. Population Definitions

All enrolled patients will be evaluated and used for presentation and analysis of the data. However, group E patients will be excluded from statistical comparison analyses due to the limited numbers of subjects expected in this group. The definition for Group E patients can be found in section 4.1.

4. STATISTICAL METHODS

4.1. Sample Size Justification

Mutually exclusive subgroups of patients suffering from neurogenic dysphagia have been identified upfront:

A. Neurogenic dysphagia as a result of stroke but not requiring mechanical ventilation or tracheotomy
B. Neurogenic dysphagia as a result of stroke and requiring mechanical ventilation or tracheotomy
C. Neurogenic dysphagia associated with mechanical ventilation but not related to stroke or traumatic brain injury (TBI)
D. Neurogenic dysphagia as a result of TBI or spinal cord injury with or without the need of mechanical ventilation or tracheotomy
E. Neurogenic dysphagia as a result from any other cause not associated with any element of groups A-D

This study attempts to populate each subgroup with 60 patients, which is a relevant sample to determine absence of occurrence of an event (e.g. device deficiency) in less than 5% of the population with a confidence level of 80%. It is anticipated that in the fifth group, only about 50% of the intended number of patients can be enrolled within the foreseen time period because of unavailability of patients in the study centers. Redistribution of the number of patients in short in this group to other subgroups will be done during the study when this issue becomes apparent. In order to count for lost-to-follow-up patients, an additional 10% of patients will be enrolled.

As such it is intended to enroll ((4×60) + (1×30)) + 10% = 297 patients. The target number is thus set at 300 patients for the total study population.

4.2. General Statistical Methods and Data Handling

4.2.1. General Methods

All output will be incorporated into Microsoft Word files and formatted to the appropriate page size(s).

Tabulations will be produced for appropriate demographic, baseline, efficacy, and safety parameters. For categorical variables, summary tabulations of the number and percentage within each category (with a category for missing data) of the parameter will be presented. For continuous variables, the mean, median, standard deviation, minimum and maximum values will be presented. Time to event data will be presented using Kaplan-Meier curves.

4.2.2. Computing Environment

All descriptive statistical analyses will be performed using SAS statistical software (Version 9.4), unless otherwise noted.

4.2.3. Withdrawals, Dropouts, Loss to Follow-up

Patients may be replaced if PES treatment is not deliverable (i.e. Phagenyx catheter insertion is not possible). Patients may withdraw from the investigation for any reason at any time without

their standard of care being affected. Patients that withdraw after receiving the PES treatment are considered as being enrolled in the study; they will not be replaced upon voluntary withdrawal. All discontinuations will be documented along with the reason for withdrawal. Patient data collected up to the point of voluntary withdrawal will still be used.

4.2.4. Missing, Unused, and Spurious Data

Unless otherwise noted, efficacy analyses will be based on observed data only. NO missing data will be imputed.

For partial dates where the start day is missing, the first of the month will be imputed as the day.

4.3. Demographic and Baseline Characteristics

Demographic and baseline information will be summarized for the Total Patient Population by index group using descriptive statistics. Comparison will be performed between groups by Fischer's exact test, Chi-square test, Kruskal-Wallis test, or one-way analysis of variance (ANOVA). Pseudo SAS code for each statistical analysis can be found in section 7.3.

The endpoints to be summarized are age, sex (male), days from onset to treatment (OTT), feeding status, GCS, NIHSS, mRS, type of stroke (side of lesion (left), bilateral lesion, and infratentorial), tracheal cannula in situ, oxygen use in ICU, and duration of ventilation.

OTT is defined as (date of treatment on Day 1 – date of dysphagia causal event + 1).

Patients will be considered to have tracheal cannula in situ if patients answered 'Yes' to question 1 (Is patient still tracheotomised?) at baseline on the Decannulation Efforts form.

Patients will be considered to have oxygen use in ICU if patients in groups A and E answered 'b' or patients in groups B, C, or D answered 'b', 'c', or 'd' to question 1 (Patient currently requires) at baseline on the Ventilation Status form.

Duration of artificial or mechanical ventilation will be calculated for patients who received oxygen support via orotracheal or tracheotomy tube. Duration of ventilation is defined as (stop date of oxygen support via orotracheal tube – start date of oxygen support via orotracheal tube) + 1 if patient requires oxygen support via orotracheal tube and (stop date of oxygen support via tracheotomy tube – start date of oxygen support via tracheotomy tube) + 1 if patient requires oxygen support via tracheotomy tube.

The endpoints will be compared across indexes with the following statistical tests:

Statistical Comparison Test	Endpoint(s)
Fischer's Exact	Type of stroke (Side of lesion (left), bilateral lesion, infratentorial lesion)
Chi-square	Sex (male), feeding status, tracheal cannula in situ, oxygen use in ICU
Kruskal-Wallis	OTT
One-way ANOVA	Age, GCS, NIHSS

4.4. Efficacy Evaluation

4.4.1. PAS, DSRS, FOIS, Length of Stay in Hospital, and Death by Index Group

PAS, DSRS, FOIS, length of stay in hospital, and death will be summarized by index group and time point as applicable. Mean and standard deviation will be provided for PAS, DSRS, and

FOIS. Median and IQR will be provided for the length of hospital stay in days. Frequency and percent of death by index will also be provided. Mean difference (Day 92 – Baseline) and the associated 95% CI will be provided for DSRS and FOIS and the regression coefficient for the day effect and associated 95% CI will be provided for PAS. The mean difference and 95% CI will be calculated by using a multiple linear regression with covariates of baseline value, age, and time since ictus. Time since ictus will be defined similarly to OTT, (date of treatment on Day 1 – date of dysphagia casual event + 1). The regression coefficient for the day effect and associated 95% CI will also come from the same multiple linear regression model.

Comparisons between index groups for PAS, DSRS, and FOIS for mean difference after treatment will be conducted using ANOVA. The length of hospital stay in days will be compared across indexes with the Kruskal-Wallis test and death will be compared across indexes with the Chi-square test.

The following figures will be presented:

- Line plot of mean (±SD) DSRS over time (baseline, Day 5, Day 9, and Day 92) for all patients
- Line plot of mean (±SD) FOIS and PAS over time (baseline, Day 5, Day 9, and Day 92) for all patients
- Line plot of mean (±SD) DSRS and FOIS over time (baseline, Day 5, Day 9, and Day 92) by tracheotomy status
- Kaplan-Meier plot for time to discharge and/or start of feeding. Time to discharge is defined as (date of discharge – date of admission to hospital) + 1. Time to start of feeding is defined as (date of visit where feeding status is 'eats exclusively orally without any supervision', 'eats exclusively orally with supervision', or 'eats exclusively orally with support from staff' – date of admission to hospital) + 1. The shorter time between the two will be used as time to event.

4.4.2. Effect of PES on Decannulation Status

The effect of PES will be summarized overall and by decannulation status after PES. Mean and standard deviation will be provided for age, GCS, NIHSS, PES parameters (mean threshold, mean tolerance, mean stimulation intensity), DSRS, FOIS, PAS,

Median and IQR will be provided for OTT in days, mRS, and length of hospital stay in days.

Count and median will be provided for duration of ventilation in days and time from final PES to decannulation in days.

Frequency and percent will be provided for sex, type of stroke (ischaemic; haemorhaggic; side of lesion, left; bilateral lesion; and infratentorial), acute stroke treatments (thrombolytics; thrombectomy; surgery; anticoagulants; antiplatelets; or other), other causes of dysphagia (critical illness polyneuropathy; encephalitis; Guillain-Barre syndrome; meningitis; multiple sclerosis; TBI; or other), feeding status (oral, no supervision; oral, with supervision; NGT or NJT; PEG or RIG; other feeding route, or unknown), and death.

Comparison between decannulation status groups will be conducted for feeding status and death by the Chi-square test. Length of hospital stay in days will be compared between decannulation status groups by the Kruskal-Wallis test.

4.4.3. User Experience of PES

User experience of PES will be summarized across indexes with mean and standard deviation for time to insert catheter, number of insertion attempts, score of ease of positioning, and number of catheters used for 3-day treatment.

4.4.4. PAS, DSRS, FOIS, Length of Stay in Hospital, and Death by Active and Sham Groups of PHAST-TRAC

The same endpoints in section 4.4.2 will be analyzed by active and sham groups of PHAST-TRAC as well as ventilated and not ventilated. 'Ventilated' is comprised of groups B, C, and D if ventilated and 'Not Ventilated' is comprised of groups A and D if not ventilated. Group definitions can be found in section 4.1.

4.4.5. Baseline Characteristics in Ventilated versus Non-ventilated Participants and Supratentorial/Anterior Circulation versus Infratentorial/Posterior Circulation Stroke Participants

The same endpoints in section 4.4.2 will be analyzed by ventilated and non-ventilated patients as well as by anterior versus posterior stroke participants. 'Ventilated' is comprised of groups B, C, and D if ventilated and 'Not Ventilated' is comprised of groups A and D if not ventilated. Supratentorial/anterior and infratentorial/posterior stroke participants are from groups A and B. Supratentorial/anterior patients are patients who experienced a stroke that affected the right, left, or bilateral sides of the brain. Infratentorial/posterior stroke patients are identified directly on the CRF.

4.4.6. Stimulation Levels

Stimulation levels of threshold, tolerance, and stimulation will be summarized by day across indexes and decannulation status after PES separately. Mean and standard deviations will be provided, and p-values from a one-way ANOVA will be provided for comparison.

The following figures will be presented:

- Line plot of mean (±SD) over time (Day 1, Day 2, and Day 3) by stimulation level and index
- Line plot of mean (±SD) over time (Day 1, Day 2, and Day 3) by stimulation level and decannulation status

4.4.7. DSRS Subscales (Fluids, Diet, Supervision) by Index Group and Timing

DSRS subscales of fluids, diet, and supervision will be summarized by day across indexes and decannulation status after PES separately. Mean and standard deviations will be provided at each day. The mean difference after treatment, associated 95% CI, and p-value will also be provided from a multiple linear regression model adjusted for baseline value, age, and time since ictus.

4.4.8. PAS, DSRS, FOIS, Length of Stay in Hospital, and Death by Circulation Status (Supratentorial and Infratentorial)

PAS, DSRS, FOIS, length of stay in hospital, and death will be summarized across circulation status groups of supratentorial (anterior circulation) and infratentorial (posterior circulation).

Mean and standard deviations will be provided at each day for DSRS, FOIS, and PAS. The mean difference after treatment, associated 95% CI, and p-value will also be provided from a multiple linear regression model adjusted for baseline value, age, and time since ictus. T-tests will be performed at baseline to compare supratentorial versus infratentorial patients.

Median and IQR will be provided for the length of hospital stay in days. Frequency and percent of death by index will also be provided. Comparison between circulation status groups will be conducted for death by the Chi-square test. Length of hospital stay in days will be compared between circulation status groups by the Kruskal-Wallis test.

4.4.9. PAS, DSRS, FOIS, Length of Stay in Hospital, and Death by Decannulation Status after PES

PAS, DSRS, FOIS, length of stay in hospital, and death will be summarized across decannulation status groups. Mean and standard deviations will be provided at each day for DSRS, FOIS, and PAS. The mean difference after treatment, associated 95% CI, and p-value will also be provided from a multiple linear regression model adjusted for baseline value, age, and time since ictus. T-tests will be performed at baseline to compare decannulated after PES versus not decannulated after PES patients.

Median and IQR will be provided for the length of hospital stay in days. Frequency and percent of death by index will also be provided. Comparison between decannulation status groups will be conducted for death by the Chi-square test. Length of hospital stay in days will be compared between decannulation status groups by the Kruskal-Wallis test.

4.4.10. PAS, DSRS, FOIS, Length of Stay in Hospital, and Death by PHADER Stroke and STEPS sham Groups

PAS, DSRS, FOIS, length of stay in hospital, and death will be summarized across STEPS PES, STEPS sham, and PHADER PES groups. Mean and standard deviation will be provided at baseline, Day 9 (PHADER only), Day 14 (STEPS only), and Day 90 for PAS, DSRS, and FOIS. STEPS and PHADER will be compared at Day 14 and Day 9 respectively. Difference in means and 95% CI will be provided. P-value will be provided from a multiple linear regression of mean on treatment value with adjustment for age, sex, onset from stroke to treatment in days, average treatment current, and baseline values of PAS, DSRS, and FOIS. Average treatment current is defined as the sum of intensity of stimulation actually delivered across all treatment days, divided by the number of treatment days.

Median and IQR will be provided for length of hospital stay for all groups. Comparison between PHADER stroke and STEPS sham groups will be conducted with an odds ratio for the length of hospital stay and a hazard ratio for deaths. P-values will be obtained from an ordinal logistic regression for length of hospital stay and from the Cox proportional hazard regression for deaths.

4.5. Safety Analyses

4.5.1. Serious Adverse Events

Serious adverse events will be displayed in tables and listings using System Organ Class (SOC) and Preferred Term.

The total number and percentage of serious adverse events will be summarized by indexes and overall. Patient incidence rates, i.e. a patient contributes only once to the count for a given

specific adverse event (preferred term) will be summarized by relationship to device or treatment (yes or no), indexes, and overall. Median and IQR will be provided in days for treatment to event for each preferred term overall.

P-values will be provided for comparison between indexes for each preferred term from Chi-square tests.

5. CHANGES TO PLANNED ANALYSES

The following are changes between the protocol-defined statistical analyses and those presented in this statistical analysis plan:

- The following secondary objectives will not be analyzed due to insufficiency of data:
    - To assess the severity of secretion by means of a standard scoring system
    - To describe the ease and appreciation by speech and language therapists (SLTs) and physicians delivering PES
    - To describe the SLT management plan prior-PES and its execution in conjunction with PES
    - To describe the SLT therapy between causal event, prior- and after-PES treatment
- The following secondary efficacy endpoints will not be analyzed due to insufficiency of data:
    - Outcome of DSRS and other standard scores related to the time of the day PES is first delivered
    - Patient's QoL using the EURO-QoL-5D questionnaire
    - Disease severity using the Barthel Index

6. REFERENCES

1 Chris Fraser, M.P. (2002). Driving Plasticity in Human Motor Cortex is Associated with Improved Motor Function after Brain Injury. *Neuron*, 831-840.

7. CLINICAL STUDY REPORT APPENDICES

7.1. Statistical Tables to be Generated

Table 1	Baseline characteristics by index group. Data are number (%), median [interquartile range] or mean (standard deviation); comparison by Fisher's exact test, Chi-square test, Kruskal-Wallis test or one-way analysis of variance
Table 2	Penetration aspiration score, dysphagia severity rating scale, functional oral intake scale and length of stay in hospital by index group and timing. Data are mean (standard deviation). Analysis of mean difference after treatment with adjustment for baseline value, age and time since ictus using multiple linear regression, or Chi-Square test or Kruskal-Wallis test
Table 2a	Effect of PES on decannulation status. Data are number (%), median [interquartile range] or mean (standard deviation)
Table 3	User experience of PES
Table 4	Dysphagia severity rating scale, functional oral intake scale, penetration aspiration scale, and length of stay in hospital by index group and timing, and in active and sham groups of PHAST-TRAC. Data are mean (standard deviation). Analysis of mean difference after treatment with adjustment for baseline value, age and time since ictus using multiple linear regression, or Chi-Square test or Kruskal-Wallis test
Supplemental Table II	Baseline characteristics in ventilated versus non-ventilated participants (Groups A-D), and anterior versus posterior circulation stroke participants (Groups A, B). Data are number (%), median [interquartile range] or mean (standard deviation); comparison by Fisher's exact test, Chi-square test, Kruskal-Wallis test or one-way analysis of variance
Supplemental Table III	Stimulation levels by subgroup
Supplemental Table IIIa	Stimulation levels by decannulation status
Supplemental Table IV	Dysphagia severity rating scale subscales (fluids, diet, supervision) by index group and timing. Analysis of mean difference after treatment with adjustment for baseline value, age and time since ictus using multiple linear regression
Supplemental Table IVa	Dysphagia severity rating scale subscales (fluids, diet, supervision) by index group and timing. Analysis of mean difference after treatment with adjustment for baseline value, age and time since ictus using multiple linear regression
Supplemental	Dysphagia severity rating scale, functional oral intake scale,

Table V	penetration aspiration scale, and length of stay in hospital for participants with an index event of stroke (group A or B). Data are mean (standard deviation). Analysis of mean difference after treatment with adjustment for baseline value, age and time since ictus using multiple linear regression, or Chi-Square test or Kruskal-Wallis test
Supplemental Table Va	Dysphagia severity rating scale, functional oral intake scale, penetration aspiration scale, and length of stay in hospital for participants decannulated or not decannulated after PES. Data are mean (standard deviation). Analysis of mean difference after treatment with adjustment for baseline value, age and time since ictus using multiple linear regression, or Chi-Square test or Kruskal-Wallis test
Supplemental Table VI	Serious adverse events. Data are number (%); comparison by Chi-square test
Supplemental Table VIIa	Penetration aspiration score, dysphagia severity rating scale, functional oral intake scale and length of stay in hospital in PHADER stroke (non-ventilated, Group A) versus STEPS sham groups. Data are mean (standard deviation), difference in means and 95% confidence intervals. Comparison using Cox proportional hazard regression, ordinal logistic regression, multiple linear regression of mean on treatment value with adjustment for age, sex, onset from stroke to treatment, average treatment current, and baseline value.

7.2. Figures to be Generated

Figure 1	Dysphagia severity rating scale, all patients. Line plot with mean (+/-SD)
Figure 2	Occurrence of SAE's over time
Figure 3	PES treatment parameters (threshold, tolerance, stimulation), by subgroup. Line plot with mean (+/-SD)
Figure 4	PES treatment parameters (threshold, tolerance, stimulation), by decannulated or non-decannulated. Line plot with mean (+/-SD)
Figure 5	Kaplan-Meier; time to discharge and/or start of oral feeding
Supplemental Figure I	Line plot of i) FOIS and ii) PAS
Supplemental Figure II	Line plot of DSRS and FOIS in i) Tracheotomised patients (groups B, C and some of D) and ii) Non-tracheotomised patients (groups A, some of D)

7.3. Pseudo SAS Code

7.3.1. Fischer's Exact Test

```
proc freq data = qs;
	tables dependent*independent / fischer;
run;
```

7.3.2. Chi-square Test

```
proc freq data = qs;
	tables dependent*independent / chisq;
run;
```

7.3.3. Kruskal-Wallis Test

```
proc npar1way data = qs;
	class independent;
	var dependent;
run;
```

7.3.4. Multiple Linear Regression

```
proc glm data = qs;
	by subgroup;
	class visit;
	model score = visit age tti base;
	estimate 'day 92-baseline' visit -1 0 0 1;
run;
```

Where,
subgroup = categorical column headers
visit = categorical time point
score = continuous DSRS, FOIS, or PAS score
age = continuous age
tti = continuous time to ictus at each visit
base = baseline DSRS, FOIS, or PAS score

7.3.5. One-way ANOVA

```
proc glm data = qs;
    class independent;
    model dependent = independent;
    means independent;
run;
```

Phagenesis_PHADER_SAP_v1.0_20190830

Final Audit Report 2019-08-30

Created:	2019-08-30
By:	Kelly Huang (Kelly.Huang@cytel.com)
Status:	Signed
Transaction ID:	CBJCHBCAABAAnVYqyEYfVawZYN48YTA1xlcFWyGm9-p_

"Phagenesis_PHADER_SAP_v1.0_20190830" History

Document created by Kelly Huang (Kelly.Huang@cytel.com)
2019-08-30 - 1:22:33 PM GMT- IP address: 209.190.161.6

Document emailed to Satish Mistry (satish.mistry@phagenesis.com) for signature
2019-08-30 - 1:24:01 PM GMT

Email viewed by Satish Mistry (satish.mistry@phagenesis.com)
2019-08-30 - 1:25:16 PM GMT- IP address: 82.31.233.154

Satish Mistry (satish.mistry@phagenesis.com) verified identity with phone authentication using the phone number +XX XXXXXX9762
2019-08-30 - 1:40:04 PM GMT

Document e-signed by Satish Mistry (satish.mistry@phagenesis.com)
Signature Date: 2019-08-30 - 1:40:06 PM GMT - Time Source: server- IP address: 82.31.233.154

Document emailed to Kelly Huang (Kelly.Huang@cytel.com) for signature
2019-08-30 - 1:40:08 PM GMT

Email viewed by Kelly Huang (Kelly.Huang@cytel.com)
2019-08-30 - 1:41:59 PM GMT- IP address: 73.188.32.70

Kelly Huang (Kelly.Huang@cytel.com) verified identity with Adobe Sign authentication
2019-08-30 - 1:44:06 PM GMT

Document e-signed by Kelly Huang (Kelly.Huang@cytel.com)
Signature Date: 2019-08-30 - 1:44:06 PM GMT - Time Source: server- IP address: 73.188.32.70

Notification of signed document emailed to Satish Mistry (satish.mistry@phagenesis.com) and Kelly Huang (Kelly.Huang@cytel.com)
2019-08-30 - 1:44:06 PM GMT
